# Novel Morpholine-Bearing Quinoline Derivatives as Potential Cholinesterase Inhibitors: The Influence of Amine, Carbon Linkers and Phenylamino Groups

**DOI:** 10.3390/ijms231911231

**Published:** 2022-09-23

**Authors:** Cheng Liu, Li-Ning Wang, Yu-Ming Liu

**Affiliations:** 1 Department of Pharmacy Engineering, Tianjin University of Technology, Tianjin 300384, China; 2 College of Traditional Chinese Medicine, Tianjin University of Traditional Chinese Medicine, Tianjin 300193, China; 3 Tianjin Key Laboratory of Drug Targeting and Bioimaging, Tianjin University of Technology, Tianjin 300384, China

**Keywords:** Alzheimer’s disease, anti-cholinesterase activity, antioxidant, quinoline

## Abstract

A series of novel 4-*N*-phenylaminoquinoline derivatives containing a morpholine group were designed and synthesized, and their anti-cholinesterase activities and ABTS radical-scavenging activities were tested. Among them, compounds **11a, 11g**, **11h**, **11j**, **11l,** and **12a** had comparable inhibition activities to reference galantamine in AChE. Especially, compound **11g** revealed the most potent inhibition on AChE and BChE with IC_50_ values of 1.94 ± 0.13 μM and 28.37 ± 1.85 μM, respectively. The kinetic analysis demonstrated that both the compounds **11a** and **11g** acted as mixed-type AChE inhibitors. A further docking comparison between the **11a**- and **12a**-AChE complexes agreed with the different inhibitory potency observed in experiments. Besides, compounds **11f** and **11l** showed excellent ABTS radical-scavenging activities, with IC_50_ values of 9.07 ± 1.34 μM and 6.05 ± 1.17 μM, respectively, which were superior to the control, Trolox (IC_50_ = 11.03 ± 0.76 μM). It is worth noting that 3-aminoquinoline derivatives **12a**–**12d** exhibited better drug-like properties.

## 1. Introduction

Alzheimer’s disease (AD) is the most common neurodegenerative disease, which seriously endangers human health and life [1,2]. Its common symptoms are memory impairment, thinking loss, and cognitive decline [3]. It is estimated that there are more than 50 million people living with AD worldwide, and this number would increase to 152 million by 2050 [4].

The cause of AD is unclear, but the loss of cholinergic neurotransmission [5], deposition of β-amyloid (Aβ) plaques [6], accumulation of hyperphosphorylated tau-protein [7], and increased oxidative stress [8] have been considered as the main inducing factors of AD, and various therapeutic approaches have been proposed.

The currently more accepted hypothesis is the cholinergic hypothesis, in which clinical symptoms of senile cognitive impairment are due to the reduction of acetylcholine formation and release caused by cholinergic neuron damage. Cholinesterase (ChE), including acetylcholinesterase (AChE) and butylcholinesterase (BChE), can affect the function of cholinergic neurons by hydrolyzing acetylcholine (ACh) [9]. Normally, BChE functions as an auxiliary enzyme of AChE and plays a synergistic regulatory role in the regulation of ACh level. However, in advanced AD, AChE activity may be reduced to 55–67% of normal levels in specific areas of the brain, while BChE activity is increased. In affected cortical areas of AD, the ratio of BChE to AChE drastically changed from 0.5 to 11. Moreover, in an AD mouse model with BChE gene knocked out; there were up to 70% fewer fibrillar Aβ brain plaques, suggesting diminished BChE activity should be very necessary [10]. Therefore, inhibitors of both AChE and BChE may be attractive targets for combating AD [11,12].

Oxidative stress is another important cause of Alzheimer’s Disease [13]. Under normal physiological conditions, the level of oxidant generation is in balance with the antioxidant capacity, but the generation of oxidants could exceed the antioxidant capacity of cells in AD [14]. Oxidative stress or high levels of reactive oxygen species (ROS) are prevalent in brain regions of AD where Aβ is deposited. Antioxidant mechanisms have been thus considered as an effective therapeutic strategy in AD to increase the viability of surviving neurons [15].

**Figure 1:**
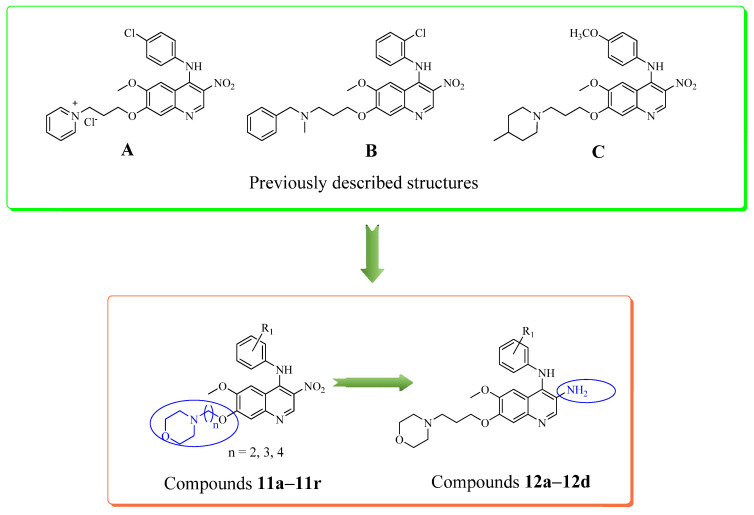
Design strategy for morpholine-bearing quinolone derivatives.

Considering the multifactorial pathogenesis of Alzheimer’s disease, we continuously exploit multifunctional agents that can simultaneously inhibit AChE and BChE and exert antioxidant effects. Three representative compounds (**A**–**C**) of three 4-*N*-phenylaminoquinoline series were reported in our previous study (Figure 1) [16,17,18], and some derivatives exhibited significant ChE inhibitions and antioxidant activities. In addition, a kinetic analysis of AChE inhibition and molecular docking study indicated that that these analogs could bind to both the catalytic active site (CAS) and peripheral anionic site (PAS) of AChE. As a part of our ongoing project, we remained the quinoline core as a privileged backbone, and introduced different substituents to its 4-*N*-phenyl ring. In contrast, in this study, we aimed to reduce the nitro group and alter the carbon linker lengths of the alkoxy side chain to explore their effect on ChE inhibitory activities. Our design is also based on bioisosteric replacement with a morpholine moiety (Figure 1), as it has a lower structural weight and relatively small volume for improving brain exposure, which was inspired by this pharmacophoric unit with strong binding interactions to ChE [19,20,21].

Now in the present study, a new series of 4-*N*-phenylaminoquinoline derivatives (**11a**–**11r** and **12a**–**12d**) with different lengths of carbon linker were designed, synthesized, and tested for their anti-ChE activities, enzyme kinetic analysis, and ABTS radical-scavenging activities.

## 2. Results and Discussion

### 2.1. Chemistry

According to Figure 2, compounds **11a**–**11r** and **12a**–**12d** were synthesized starting from the commercially available material vanillic acid. Compounds **2**–**9**, **10a**–**10r** (Figures S1–S6) were synthesized as our earliest reported procedures [16,17,18]. Compounds **11a**–**11r** were achieved upon the substitution of compound **10a**–**10r** with excess morpholine, respectively. Furthermore, compounds **11a**–**11d** were transformed into compounds **12a**–**12d** after nitro reduction. All target compounds were purified by column chromatography and characterized by ^1^H NMR, ^13^C NMR, and HR-ESI-MS (Figures S7–S28). Based on the NOESY spectra of typical compounds **11f**, **11l** and **11r**, the phenylamino groups were determined to be on the quinolone ring due to the NOESY correlations between NH and H-5; meanwhile, the morpholine groups were linked to the aliphatic moieties by the NOESY correlation of compound **11f** from H-2‴ and H-6‴ to H-2″ and H-3″, by the NOESY correlation of compound **11l** from H-2‴ and H-6‴ to H-2″, and by the NOESY correlation of compound **11r** from H-2‴ and H-6‴ to H-3″.

### 2.2. Biological Evaluation

The Ellman’s method was used to assess the ChE inhibitory activities of all synthesized quinoline derivatives (**11a**–**11r** and **12a**–**12d**) on ChE [22]. The inhibitory potencies are showed in Table 1. The results indicated that most derivatives were dual inhibitors of both AChE and BChE. Compound **11g** revealed the most potent inhibition to AChE and BChE with IC_50_ values of 1.94 ± 0.13 μM and 28.37 ± 1.85 μM, respectively, comparable to our reference, galantamine. Structure–activity relationship analysis of **11a**–**11r** showed that their ChE inhibitory potency was closely related to their methylene side chain length and substituted group of 4-*N*-phenyl ring. As shown in Table 1, the derivatives with a 2-methylene linker between quinoline and morpholine (**11g**–**11l**) showed better inhibition of AChE than those with 3- or 4-methylene (**11a**–**11f** or **11m**–**11r**). Interestingly, the obvious regularity was applicable to BChE inhibition as well. Thus, it could be speculated that in the compounds **11a**–**11f** or **11m**–**11r**, the length of the 3- or 4-methylene side chain isn’t suitable to bind to the CAS and PAS of the enzyme simultaneously. Besides, compounds **11b**, **11h**, and **11n**, substituted by a chlorine atom at the 2-position of the 4-*N*-phenyl ring, displayed better AChE inhibitory activities than the corresponding compounds **11c**, **11d**, **11i**, **11j**, **11o**, and **11p**, which had a chloro group at the 3- or 4-position. Meanwhile, for these derivatives bearing a *para*-substituted electron-donating group (**11e**, **11f**, **11k**, **11l**, **11q**, **11r**), the order of the AChE inhibitory potency was methoxy group > hydroxyl group, which was the same as the trend for BChE inhibitory activities. Unexpectedly, compounds **11a**, **11g**, and **11m** without any substituents on the 4-*N*-phenyl ring showed much better inhibitory potencies on AChE than the other compounds with the same chain length. Thus it seemed that the small volume of aromatic moieties would be positive for the inhibition of AChE.

As for the same side chain, compounds **12a**, **12b**, and **12c** with 3-amino groups revealed better BChE inhibitory activities than their corresponding 3-nitro derivatives **11a**, **11b**, and **11c**, while compound **12c** and **12d** with a 3-amino group showed better inhibition of AChE than their corresponding 3-nitro derivatives **12c** and **11d**, which indicated that the inhibition order of BChE was inconsistent with that of AChE after nitro reduction. Compound **12c**, bearing a meta-chloro substituent of a 4-N-phenyl ring, was more potent towards BChE inhibition than the ortho-chloro derivative **12b** and para-chloro derivative **12d**; meanwhile, **12c** and **12d** demonstrated similar potency against AChE, being more active than **12b**. Taken as a whole, nearly all SI values were greater than one, so most compounds were characterized by their stronger inhibitory activities on AChE than on BChE.

### 2.3. Enzyme Kinetic Analysis against AChE

**Figure 3:**
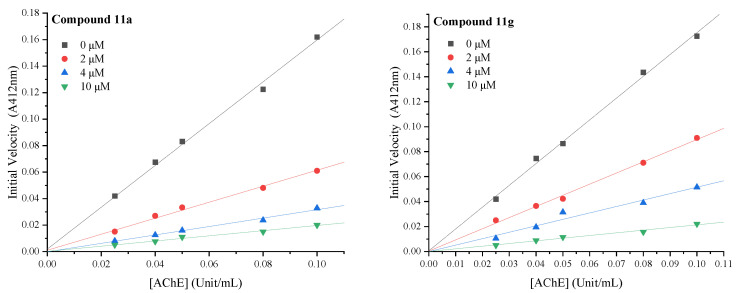
Plots of initial velocity versus enzyme concentration for the inhibition of compounds **11a** and **11g** on the hydrolysis activity of AChE.

The kinetics of the highly active compounds **11a** and **11g** were further studied to understand the nature of AChE inhibition. The results of Figure 3 showed that both the compounds **11a** and **11g** were reversible inhibitors, as in the presence of different concentrations of compounds; plots of the initial velocity versus enzyme concentration gave a series of straight lines, all of which passed through the origin.

**Figure 4:**
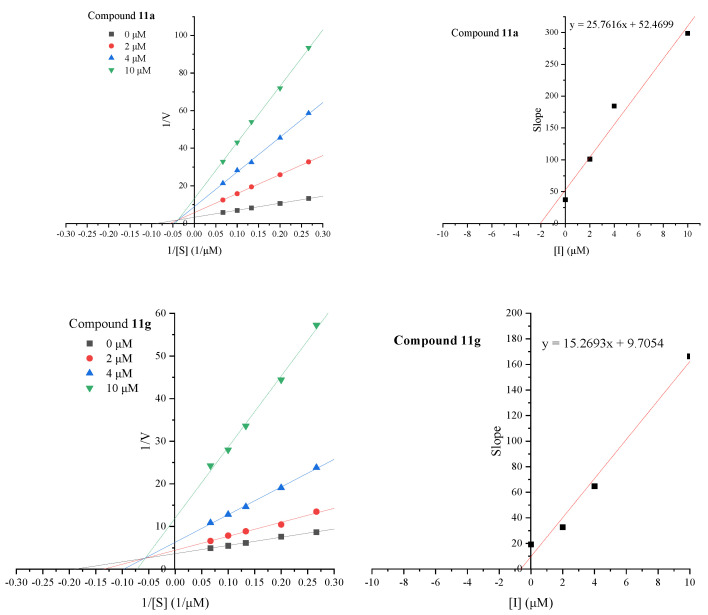
(**Right**): Lineweaver–Burk plots for the inhibition of AChE by compounds **11a** and **11g** at different concentrations of substrate (ATCI), (**Left**): Secondary plots for calculation of steady-state inhibition constants (*K*_i_) of compounds **11a** and **11g**.

The kinetic characteristics of two active compounds, **11a** and **11g**, against AChE was also studied to elucidate the type of inhibition and the inhibition constant. The analysis was carried out by measuring the enzyme’s activity at different concentrations of substrate (0 μM–10.0 μM). The acquired inhibition data were further depicted with the Lineweaver–Burk method followed by inhibition constant (*K*_i_) calculation using Lineweaver–Burk secondary plots. Results in Figure 4 show that compounds **11a** and **11g** had a family of lines with a common intercept on the left of the vertical axis and above the horizontal axis. All of these lines had no intersection on the horizontal or vertical axis, indicating that compounds **11a** and **11g** cause a mixed type of inhibition. This pattern of inhibition is usually the result of a combination of partially competitive and non-competitive inhibitors [9]. Then, the *K*_i_ values for compounds **11a** and **11g** were calculated as 2.03 μM and 0.64 μM, respectively.

### 2.4. ABTS Radical-Scavenging Activity

The ABTS assay was designed to determine the antioxidant capacities of compounds **11a**–**11r** and **12a**–**12d**. As shown in Table 2, compounds **11e**, **11f**, **11g**, **11k**, **11l**, **11q** and **11r** had good radical-scavenging activities. Interestingly, most of these compounds contain hydroxyl or methoxy groups. Amazingly, compound **11k** (IC_50_ = 13.55 ± 3.23 μM) possessed a comparable free radical-scavenging ability with the positive control Trolox (IC_50_ = 11.03 ± 0.76 μM), while both the compounds **11f** (IC_50_ = 9.07 ± 1.34 μM) and **11l** (IC_50_ = 6.05 ± 1.17 μM) exhibited higher free radical-scavenging activities than Trolox. It seemed that the 4-*N*-phenyl ring with an electron-donating group substitution at its 4-position could show distinct ABTS radical-scavenging abilities. Unexpectedly, shortening the length of the carbon linker may improve the ABTS radical-scavenging abilities.

### 2.5. Docking Studies

To explore possible interaction modes in the active site of AChE or BChE, we selected the typical compounds **11a**, **11g**, and **12a** that have a good performance of their combined activities and used CDOCKER in Discovery Studio 3.0 software for molecular modeling studies.

**Figure 5:**
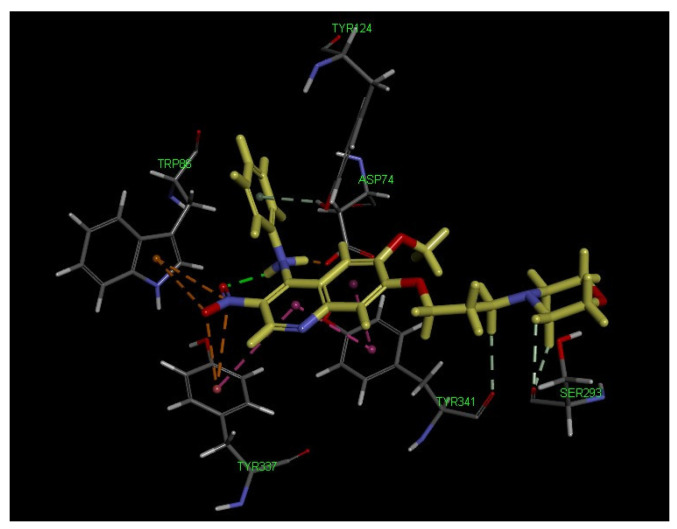
3D binding mode of compound **11a** with AChE (PDB code: 4EY7), highlighting the protein residues that participate in the main interactions with the inhibitor.

The crystal complex of AChE with donepezil (PDB: 4EY7) was used for docking studies. It was shown (Figure 5) that compound **11a** interacted with the CAS site of AChE by the following moieties: the nitro group interacts with Trp86 and Tyr337 residues to produce two π-anions. The quinoline ring formed one π–π interaction with Tyr337. It is worth mentioning that compound **11a** binds to the PAS site of AChE with four amino acids as the following: the amino acid residue Tyr341 established two π–π interactions with the quinoline ring and one carbon hydrogen bond with the methylene side chain; the morpholine moiety produces two carbon hydrogen bonds with Ser293; one salt bridge was created between the amino hydrogen on the 4-*N*-phenylamino group and Asp74, and one π-donor hydrogen bond was created between the benzene ring and Tyr124. All these interactions were observed within the radius of 5 Å. Interestingly, such residues as Asp74, Trp86, Tyr124, Tyr337, and Tyr341 have also been reported to involve the ligand–receptor complexes of tacrine, galantamine, huperzineA, and donepezil [24,25,26]. The interaction mode indicated that compound **11a** could behave as a dual binding site inhibitor of AChE, which is consistent with our kinetic analysis.

**Figure 6:**
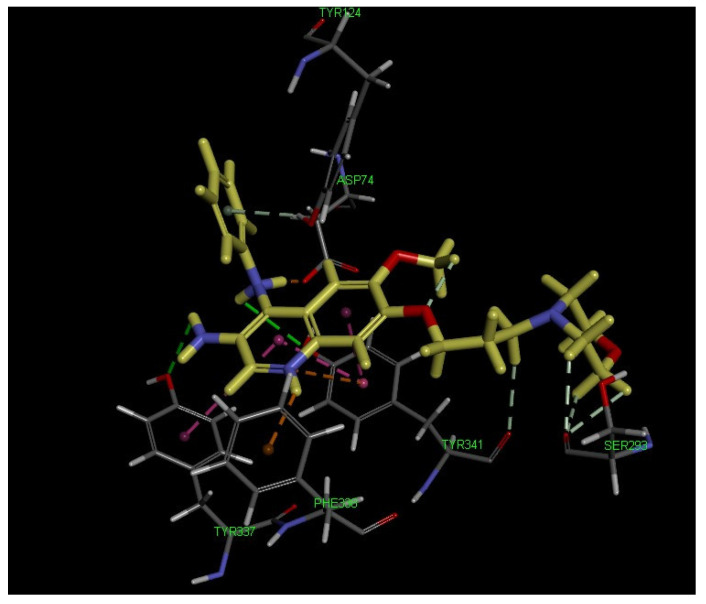
3D binding mode of compound **12a** with AChE (PDB code: 4EY7), highlighting the protein residues that participate in the main interactions with the inhibitor.

For comparison with **11a**, the docking study of another promising compound, **12a,** was performed. In the **12a**–AChE complex (Figure 6), two conventional hydrogen bonds were observed between the NH_2_ of the quinoline ring and Tyr337, and between the NH of the 4-*N*-phenylamino group and Tyr341. Three carbon hydrogen bonds were formed between the morpholine moiety and Ser293, and another carbon hydrogen bond was generated between the methylene side chain and Tyr341. One salt bridge was created between the NH of the 4-*N*-phenylamino group and Asp74. Meanwhile, compound **12a** formed three π–π interactions with Tyr337 and Tyr341 residues and also two π-cations with Phe338 and Tyr341 residues. From the above, as can be seen: the docking amino acid residues and orientation of two ligands were similar, but some binding patterns had changed with the CDOCKER energy of −25.58 and −19.22 kcal/mol for compounds **11a** and **12a**, respectively, which explained the higher AChE inhibitory potential of compound **11a** over **12a**.

**Figure 7:**
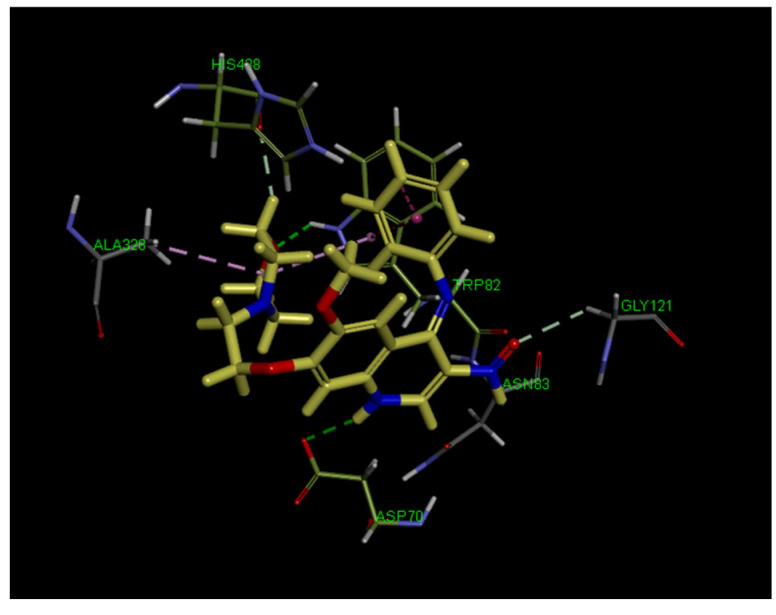
3D binding mode of compound **11g** with BChE (PDB code: 4BDS), highlighting the protein residues that participate in the main interactions with the inhibitor.

The interaction of target compound with BChE (PDB code: 4BDS) was also carried out. As seen in Figure 7, compound **11g** was bound to Gly121 from the oxyanion hole (OAH), Trp82 from the anionic substrate binding site (AS), and His438 from the catalytic triad; however, it also bound to Asn83 and Ala328 residues and one PAS amino acid residue, Asp70, via one π–alkyl, one alkyl–alkyl, one π–π stacked, two conventional hydrogen bonds, and three carbon hydrogen bonds. Briefly, it is noteworthy to say that compound **11g** interacted with important amino acid residues in the OAH, AS and PAS active sites of BChE, which makes it worthy of further study.

### 2.6. Blood–Brain Barrier Assay

For anti-Alzheimer drug candidates, the ability to cross the blood–brain barrier (BBB) and to enter the central nervous system (CNS) is crucial to achieve their pharmacological target binding and activity, and CNS drugs require more strict rules for a successful penetration. Some researchers [27,28] have limited CNS drug activity to compounds having lipophilicity (Log*P*) in a preferred range of two to four. Besides, for molecules to penetrate the BBB, a topological polar surface area (TPSA) value less than 90 Å ^2^ is usually needed and molecules with a TPSA of greater than 120 Å ^2^ tend to be poor in permeating cell membranes. Therefore, the molar mass (MW), Log*P*, and TPSA of all target compounds were simulated by ChemDraw 19.0 (Table 1). For comparison purposes, the previously described compounds (**A**–**C**) [16,17,18] were also included. It can be seen from Table 1 that both compounds **11a** and **11g**, with smaller molecular weights and suitable lipophilicity, have quite good drug-like properties. More importantly, the values of MW, Log*P*, and TPSA of compounds **12a**–**12d** revealed that the structural variation after nitro reduction led to much better drug-like results, in which their parameters are almost comparable to those of the approved AChE inhibitor galanthamine. Generally, the drug-like properties of other compounds also have obviously improved, by comparison with our previous compounds **B** [17] and **C** [16], which have higher MW and Log*P* values (compounds **A** [18] reveals the extra lower Log*P* [23] in Table 1). Because Log*P* was thought as an important physicochemical parameter to evaluate the ability to cross BBB, the Log*P* of some compounds was further assessed in experiments. The results were obtained as Log*P* values of 3.82, 3.55, 2.07, and 2.35 for the typical compounds **11a**, **11g**, **12a**, and **12c**, respectively, which indicated that the lipophilicities of these compounds were just appropriate to facilitate brain penetration.

Water solubility is regarded as another vital descriptor for BBB penetration, where high solubility is considered as a prerequisite for a rapid absorption and higher bioavailability of a drug candidate. Hence, we desired to determine the solubility data of some compounds in water using the shake-flask method. With this experiment, we have obtained their data as 0.62, 3.03, 12.9, and 10.5 mg/mL for the selected compounds **11a**, **11g**, **12a**, and **12c**, respectively. These data are much higher than the general criteria of 60 μg/mL, which would be considered “high” in drug discovery for predicting human oral absorption [29].

## 3. Materials and Methods

### 3.1. General

The chemical reactions were monitored by TLC using commercially available alumina plates coated with silica gel 60 F254 (Merck). Chromatographic separation was performed on self-packed columns with silica gel from Qingdao Haiyang Chemical Group Co., Ltd. (PR China), and MPLC was carried out on a BUCHI apparatus equipped with a C-605 pump. ^1^H and ^13^C NMR spectra were recorded on a Bruker Avance III 400 spectrometer with TMS as an internal standard. Coupling constant (*J*) values were presented in Hz, and spin multiplicities were given as s (singlet), d (doublet), t (triplet), q (quartet), br.s (broad singlet) and m (multiplet). A Waters Acquity UPLC Class I/Xevo G2Q-Tof mass spectrometer was used to obtain the high-resolution ESI-MS (HR-ESI-MS) data.

### 3.2. Synthesis of Compounds ***11a**–**11r***

A mixture of compounds **10a**–**10r** (0.8 mmol), NaI (0.223 g, 1.5mmol), K_2_CO_3_ (0.552 g, 4 mmol) was refluxed in CH_3_CN (50 mL) for 0.5 h, then we added morpholine (0.8 mmol) and refluxed the mixture for 24 h. Excess CH_3_CN was rotated off, and the residue was partitioned between CH_2_Cl_2_ and H_2_O. The organic layer was collected, filtered and then evaporated to dryness to give a crude product. The crude material was purified by silica gel column chromatography using CH_2_Cl_2_/MeOH (10:1) as eluent to give **11a**–**11r**.

#### 3.2.1. 6-Methoxy-7-(3-Morpholinopropoxy)-3-Nitro-4-Phenylamino-Quinoline (**11a**)

Yield: 40%, yellow solid. ^1^H-NMR (400 MHz, CDCl_3_) δ: 2.10 (m, 2H, CH_2_), 2.49 (br.s, 4H, 2 × NCH_2_), 2.56 (t, *J* = 7.8 Hz, 2H, NCH_2_), 3.34 (s, 3H, OCH_3_), 3.73 (t-like, *J* = 4.6 Hz, 4H, 2 × OCH_2_), 4.26 (t, *J* = 7.6 Hz, 2H, OCH_2_), 6.89 (s, 1H, ArH), 7.19 (d, *J* = 7.8 Hz, 2H, 2 × ArH), 7.26 (t, *J* = 7.8 Hz, 1H, ArH), 7.36 (s, 1H, ArH), 7.41 (t, *J* = 7.8 Hz, 2H, 2 × ArH), 9.36 (s, 1H, ArH), 10.42 (s, 1H, NH); ^13^C-NMR (100 MHz, CDCl_3_) δ: 153.6, 148.2, 148.0, 145.4, 145.1, 141.0, 129.5 (2C), 128.4, 126.9, 124.7 (2C), 112.7, 110.0, 106.6, 67.4, 66.9 (2C), 55.3, 55.2, 53.7 (2C), 25.8; HR-ESI-MS (positive mode) *m/z*: 439.1992 [M+H]^+^ (calculated for C_23_H_27_N_4_O_5_, 439.1981).

#### 3.2.2. 6-Methoxy-7-(3-Morpholinopropoxy)-3-Nitro-4-(2-Chlorophenylamino)-Quinoline (**11b**)

Yield: 45%, yellow solid. ^1^H-NMR (400 MHz, CDCl_3_) δ: 2.12 (m, 2H, CH_2_), 2.52 (br.s, 4H, 2 × NCH_2_), 2.59 (t, *J* = 7.0 Hz, 2H, NCH_2_), 3.41 (s, 3H, OCH_3_), 3.75 (t-like, *J* = 4.4, 4H, 2 × OCH_2_), 4.27 (t, *J* = 6.6 Hz, 2H, OCH_2_), 6.78 (s, 1H, ArH), 6.99 (d, *J* = 7.6 Hz, 1H, ArH), 7.18 (m, 2H, ArH), 7.39 (s, 1H, ArH), 7.55 (d, *J* = 7.6 Hz, 1H, ArH), 9.37 (s, 1H, ArH), 10.15 (s, 1H, NH); ^13^C-NMR (100 MHz, CDCl_3_) δ: 153.9, 148.9, 148.0, 145.0, 143.9, 138.4, 130.5, 129.5, 128.1, 127.4, 126.4, 124.4, 113.2, 110.0, 105.2, 67.4, 66.8 (2C), 55.4, 55.2, 53.6 (2C), 25.8; HR-ESI-MS (positive mode) *m/z*: 473.1604 [M+H]^+^ (calculated for C_23_H_26_ClN_4_O_5_, 473.1591).

#### 3.2.3. 6-Methoxy-7-(3-Morpholinopropoxy)-3-Nitro-4-(3-Chlorophenylamino)-Quinoline (**11c**)

Yield: 45%, yellow solid. ^1^H-NMR (400 MHz, CDCl_3_) δ: 2.08 (m, 2H, CH_2_), 2.47 (br.s, 4H, 2 × NCH_2_), 2.55 (t, *J* = 7.2 Hz, 2H, NCH_2_), 3.41 (s, 3H, OCH_3_), 3.70 (t-like, *J* = 4.5 Hz, 4H, 2 × OCH_2_), 4.23 (t, *J* = 6.6 Hz, 2H, OCH_2_), 6.82 (s, 1H, ArH), 6.98 (d, *J* = 8.0 Hz, 1H, ArH), 7.11 (s, 1H, ArH), 7.15 (d, *J* = 8.0 Hz, 1H, ArH), 7.27 (t, *J* = 8.0 Hz, 1H, ArH), 7.35 (s, 1H, ArH), 9.32 (s, 1H, ArH), 10.16 (s, 1H, NH); ^13^C-NMR (100 MHz, CD_3_OD) δ: 153.4, 152.0, 143.5, 137.3, 135.8, 134.5, 132.2, 131.5, 129.4, 123.8, 120.3, 119.3, 118.8, 103.1, 101.2, 68.1, 65.1 (2C), 56.7, 56.5, 53.4 (2C), 24.5; HR-ESI-MS (positive mode) *m/z*: 473.1647 [M+H]^+^ (calculated for C_23_H_26_ClN_4_O_5_, 473.1591).

#### 3.2.4. 6-Methoxy-7-(3-Morpholinopropoxy)-3-Nitro-4-(4-Chlorophenylamino)-Quinoline (**11d**)

Yield: 39%, yellow solid. ^1^H-NMR (400 MHz, CDCl_3_) δ: 2.12 (m, 2H, CH_2_), 2.51 (br.s, 4H, 2 × NCH_2_), 2.58 (t, *J* = 7.1 Hz, 2H, NCH_2_), 3.44 (s, 3H, OCH_3_), 3.75 (t-like, *J* = 3.9 Hz, 4H, 2 × OCH_2_), 4.27 (t, *J* = 6.6 Hz, 2H, OCH_2_), 6.83 (s, 1H, ArH), 7.09 (d, *J* = 8.4 Hz, 2H, 2 × ArH), 7.36 (d, *J* = 8.4 Hz, 2H, 2 × ArH), 7.38 (s, 1H, ArH), 9.36 (s, ArH), 10.24 (s, 1H, NH); ^13^C-NMR (100 MHz, CDCl_3_) δ: 153.9, 148.7, 148.2, 145.1, 144.3, 139.9, 131.1, 129.7 (2C), 129.1, 124.7 (2C), 112.7, 110.0, 105.9, 67,5, 66.9 (2C), 55.3, 55.2, 53.7 (2C), 25.8; HR-ESI-MS (positive mode) *m/z:* 473.1596 [M+H]^+^ (calculated for C_23_H_26_ClN_4_O_5_, 473.1591).

#### 3.2.5. 6-Methoxy-7-(3-Morpholinopropoxy)-3-Nitro-4-(4-Hydroxylphenylamino)-Quinoline (**11e**)

Yield: 50%, yellow solid. ^1^H-NMR (400 MHz, CDCl_3_) δ: 2.08 (m, 2H, CH_2_), 2.50 (m, 4H, 2 × NCH_2_), 2.56 (t, *J* = 7.3 Hz, 2H, NCH_2_), 3.38 (s, 3H, OCH_3_), 3.73 (m, 4H, 2 × OCH_2_), 4.21 (t, *J* = 6.3 Hz, 2H, OCH_2_), 6.87 (d, *J* = 8.4 Hz, 2H, 2 × ArH), 7.00 (s, 1H), 7.09 (d, *J* = 8.4 Hz, 2H, 2 × ArH), 7.37 (s, 1H, ArH), 9.27 (s, 1H, ArH); ^13^C-NMR (100 MHz, CDCl_3_) δ: 155.9, 153.4, 147.8, 147.3, 146.3, 145.4, 132.3, 127.1, 126.5 (2C), 116.3 (2C), 112.4, 109.3, 106.7, 67.2, 66.7 (2C), 55.3, 55.1, 53.5 (2C), 25.7; HR-ESI-MS (positive mode) *m/z*: 455.1948 [M+H]^+^ (calculated for C_23_H_27_N_4_O_6_, 455.1930).

#### 3.2.6. 6-Methoxy-7-(3-Morpholinopropoxy)-3-Nitro-4-(4-Methoxyphenylamino)-Quinoline (**11f**)

Yield: 45%, yellow solid. ^1^H-NMR (400 MHz, CDCl_3_) δ: 2.10 (m, 2H, H-2″), 2.49 (br. s, 4H, H-2‴ and H-6‴), 2.55 (t, *J* = 7.0 Hz, 2H, H-3″), 3.37 (s,3H, 6-OCH_3_), 3.73 (br. s, 4H, H-3‴ and H-5‴), 3.84 (s, 3H, 4′-OCH_3_), 4.25 (t, *J* = 6.6 Hz, 2H, H-1″), 6.94 (s, 1H, H-5), 6.95 (s, *J* = 8.6 Hz, 2H, H-3′ and H-5′), 7.16 (d, *J* = 8.6 Hz, 2H, H-2′ and H-6′), 7.34 (s, 1H, H-8), 9.35 (s, 1H, H-2), 10.53 (s, 1H, NH); ^13^C-NMR (100 MHz, CDCl_3_) δ: 158.0, 153.4, 147.93, 147.90, 145.9, 145.5, 133.9, 127.7, 126.1 (2C), 114.9 (2C), 112.4, 109.9, 106.4, 67.4, 66.9 (2C), 55.7, 55.3, 55.2, 53.7 (2C), 25.9; HR-ESI-MS (positive mode) *m/z*: 469.2089 [M+H]^+^ (calculated for C_24_H_29_N_4_O_6_, 469.2087).

#### 3.2.7. 6-Methoxy-7-(2-Morpholinoethoxy)-3-Nitro-4-Phenylamino-Quinoline (**11g**)

Yield: 38%, yellow solid. ^1^H-NMR (400 MHz, CDCl_3_) δ: 2.65 (br.s, 4H, 2 × NCH_2_), 2.95 (t-like, *J* = 5.6 Hz, 2H, NCH_2_), 3.33 (s, 3H, OCH_3_), 3.76 (t-like, *J* = 4.0 Hz, 4H, 2 × OCH_2_), 4.33 (t, *J* = 5.6 Hz, 2H, OCH_2_), 6.89 (s, 1H, ArH), 7.19 (d, *J* = 7.9 Hz, 2H, 2 × ArH), 7.28 (t, *J* = 7.9 Hz, 1H, ArH), 7.34 (s, 1H, ArH), 7.40 (t, *J* = 7.9 Hz, 2H, 2 × ArH), 9.37 (s, 1H, ArH), 10.44 (s, 1H, NH); ^13^C-NMR (100 MHz, CDCl_3_) δ: 153.3, 148.2, 147.9, 145.3, 145.0, 141.2, 129.7 (2C), 128.6, 126.1, 124.0 (2C), 112.9, 110.0, 106.5, 66.9, 66.8 (2C), 56.9, 55.2, 54.1 (2C); HR-ESI-MS (positive mode) *m/z*: 425.1845 [M+H]^+^ (calculated for C_22_H_25_N_4_O_5_, 425.1825).

#### 3.2.8. 6-Methoxy-7-(2-Morpholinoethoxy)-3-Nitro-4-(2-Chlorophenylamino)-Quinoline (**11h**)

Yield: 30%, yellow solid. ^1^H-NMR (400 MHz, CDCl_3_) δ: 2.64 (br.s, 4H, 2 × NCH_2_), 2.94 (t, *J* = 5.6 Hz, 2H, NCH_2_), 3.41 (s, 3H, OCH_3_), 3.75 (t-like, *J* = 4.2 Hz, 4H, 2 × OCH_2_), 4.33 (t, *J* = 5.6 Hz, 2H, OCH_2_), 6.77 (s, 1H, ArH), 6.99 (t, *J* = 6.0 Hz, 1H, ArH), 7.20 (m, 2H, ArH), 7.37 (s, 1H, ArH), 7.56 (dd, *J* = 7.7, 1.6 Hz, 1H, ArH), 9.38 (s, 1H, ArH), 10.17 (s, 1H, NH); ^13^C-NMR (100 MHz, CDCl_3_) δ: 153.6, 148.8, 147.9, 145.1, 143.9, 138.4, 130.5, 129.5, 128.1, 127.4, 126.5, 124.5, 113.3, 110.0, 105.3, 67.1, 66.9 (2C), 57.0, 55.4, 54.1 (2C); HR-ESI-MS (positive mode) *m/z*: 459.1445 [M+H]^+^ (calculated for C_22_H_24_ClN_4_O_5_, 459.1435).

#### 3.2.9. 6-Methoxy-7-(2-Morpholinoethoxy)-3-Nitro-4-(3-Chlorophenylamino)-Quinoline (**11i**)

Yield: 40%, yellow solid. ^1^H-NMR (400 MHz, CDCl_3_) δ: 2.62 (br.s, 4H, 2 × NCH_2_), 2.92 (br.s, 2H, NCH_2_), 3.40 (s, 3H, OCH_3_), 3.73 (br.s, 4H, 2 × OCH_2_), 4.31 (t, *J* = 5.4 Hz, 2H, OCH_2_), 6.82 (s, 1H, ArH), 6.97 (d, *J* = 8.0 Hz, 1H, ArH), 7.12 (s, 1H, ArH), 7.16 (d, *J* = 8.0 Hz, 1H, ArH), 7.27 (t, *J* = 8.0 Hz, 1H, ArH), 7.33 (s, 1H, ArH), 9.33 (s, 1H, ArH), 10.18 (s, 1H, NH); ^13^C-NMR (100 MHz, CDCl_3_) δ: 148.7, 148.1, 145.1, 143.9, 142.5, 135.3, 130.6, 129.4, 125.7, 123.2, 121.3, 114.7, 113.3, 110.1, 105.9, 66.9, 66.7 (2C), 56.9, 55.5, 54.1 (2C); HR-ESI-MS (positive mode) *m/z*: 459.1422 [M+H]^+^ (calculated for C_22_H_24_ClN_4_O_5_, 459.1435).

#### 3.2.10. 6-Methoxy-7-(2-Morpholinoethoxy)-3-Nitro-4-(4-Chlorophenylamino)-Quinoline (**11j**)

Yield: 38%, yellow solid. ^1^H-NMR (400 MHz, CDCl_3_) δ: 2.67 (br. s, 4H, 2 × NCH_2_), 2.97 (br.s, 2H, NCH_2_), 3.43 (s, 3H, OCH_3_), 3.78 (br.s, 4H, 2 × OCH_2_), 4.35 (br.s, 2H, OCH_2_), 6.83 (s, 1H, ArH), 7.10 (d, *J* = 8.4 Hz, 2H, 2 × ArH), 7.35 (s, 1H, ArH), 7.36 (d, *J* = 8.4 Hz, 2H, 2 × ArH), 9.37 (s, 1H, ArH), 10.26 (s, 1H, NH); ^13^C-NMR (100 MHz, CDCl_3_) δ: 153.8, 148.6, 148.1, 145.2, 144.4, 139.8, 131.1, 129.7 (2C), 129.1, 124.7 (2C), 112.9, 110.1, 106.0, 67.0, 66.8 (2C), 56.9, 55.4, 54.1 (2C); HR-ESI-MS (positive mode) *m/z*: 459.1443 [M+H]^+^ (calculated for C_22_H_24_ClN_4_O_5_, 459.1435).

#### 3.2.11. 6-Methoxy-7-(2-Morpholinoethoxy)-3-Nitro-4-(4-Hydroxylphenylamino)-Quinoline (**11k**)

Yield: 15%, brown solid. ^1^H-NMR (400 MHz, DMSO-d_6_) δ: 3.09 (br.s, 2H, NCH_2_), 3.17 (br.s, 4H, 2 × NCH_2_), 3.67 (s, 3H, OCH_3_), 3.84 (br.s, 4H, 2 × OCH_2_), 4.57 (br.s, 2H, OCH_2_), 6.76 (d, *J* = 8.0 Hz, 2H, 2 × ArH), 7.00 (d, *J* = 8.0 Hz, 2H, 2 × ArH), 7.43 (s, 1H, ArH), 7.53 (s, 1H, ArH), 8.95 (s, 1H, ArH), 9.58 (s, 1H, NH), 9.94 (s, 1H, OH); ^13^C-NMR (100 MHz, DMSO-d_6_) δ: 155.6, 152.1, 148.7, 145.4, 143.7, 132.9, 128.5, 124.4 (2C), 116.3 (2C), 115.6, 114.5, 110.7, 105.2, 64.2, 63.7 (2C), 56.1, 55.4, 52.7 (2C); HR-ESI-MS (positive mode) *m/z*: 441.1769 [M+H]^+^ (calculated for C_22_H_25_N_4_O_6_, 441.1774).

#### 3.2.12. 6-Methoxy-7-(2-Morpholinoethoxy)-3-Nitro-4-(4-Methoxyphenyamino)-Quinoline (**11l**)

Yield: 30%, yellow solid. ^1^H-NMR (400 MHz, CDCl_3_) δ: 3.13 (br.s, 4H, H-2‴ and H-6‴), 3.34 (br.s, 2H, H-2″), 3.35 (s, 3H, 6-OCH_3_), 3.84 (s, 3H, 4′-OCH_3_), 4.00 (br.s, 4H, H-3‴ and H-5‴), 4.60 (br.s, 2H, H-1″), 6.95 (s, 1H, H-5), 6.96 (d, *J* = 8.8 Hz, 2H, H-3′ and H-5′), 7.18 (d, *J* = 8.8 Hz, 2H, H-2′ and H-6′), 7.36 (s, 1H, H-8), 9.32 (s, 1H, H-2), 10.56 (s, 1H, NH); ^13^C-NMR (100 MHz, CDCl_3_) δ: 158.1, 152.9, 147.9, 147.8, 145.9, 145.6, 133.9, 127.7, 126.1 (2C), 115.0 (2C), 112.7, 110.1, 106.6, 66.6, 66.5 (2C), 56.9, 55.7, 55.2, 54.0 (2C); HR-ESI-MS (positive mode) *m/z*: 455.1944 [M+H]^+^ (calculated for C_23_H_27_N_4_O_6_, 455.1931).

#### 3.2.13. 6-Methoxy-7-(4-Morpholinobutoxy)-3-Nitro-4-Phenylamino-Quinoline (**11m**)

Yield: 37%, yellow solid. ^1^H-NMR (400 MHz, CDCl_3_) δ:1.71 (m, 2H, CH_2_), 1.96 (m, CH_2_), 2.46 (m, 6H, 3×NCH_2_), 3.33 (s, 3H, OCH_3_), 3.73 (t-like, *J* = 4.6 Hz, 4H, 2 × OCH_2_), 4.21 (t, *J* = 6.4 Hz, 2H, OCH_2_), 6.89 (s, 1H, ArH), 7.19 (d, *J* = 8.0 Hz, 2H, ArH), 7.26 (d, *J* = 8.0 Hz, 2H, 2 × ArH), 7.32 (s, 1H, ArH), 7.41 (t, *J* = 8.0 Hz, 2H, 2 × ArH), 9.37 (s, 1H, ArH), 10.44 (s, 1H, NH); ^13^C-NMR (100 MHz, CDCl_3_) δ: 153.6, 148.2, 148.0, 145.3, 145.0, 141.2, 129.7 (2C), 128.5, 126.0, 124.0 (2C), 112.6, 109.8, 106.4, 68.9, 67.0 (2C), 58.4, 55.2, 53.7 (2C), 26.6, 23.0; HR-ESI-MS (positive mode) *m/z*: 453.2155 [M+H]^+^ (calculated for C_24_H_29_N_4_O_5_, 453.2138).

#### 3.2.14. 6-Methoxy-7-(4-Morpholinobutoxy)-3-Nitro-4-(2-Chlorophenylamino)-Quinoline (**11n**)

Yield: 41%, yellow solid. ^1^H-NMR (400 MHz, CDCl_3_) δ: 2.02 (br.s, 2H, CH_2_), 2.20 (br.s, 2H, CH_2_), 2.99 (br.s, 2H, NCH_2_), 3.19 (br.s, 2H, NCH_2_), 3.37 (s, 3H, OCH_3_), 3.51 (br.s, 2H, NCH_2_), 4.00 (br.s, 2H, OCH_2_), 4.34 (br.s, 4H, 2 × OCH_2_), 6.81 (s, 1H, ArH), 7.44 (br.s, 3H, 3×ArH), 7.66 (d, *J* = 7.7 Hz, 1H, ArH), 8.10 (s, 1H, ArH), 9.44 (s, 1H, ArH), 11.06 (s, 1H, NH); ^13^C-NMR (100 MHz, CDCl_3_) δ: 155.3, 149.6, 147.6, 139.5, 136.7, 135.8, 130.3, 130.1, 129.3, 128.0, 127.1, 126.8, 112.4, 104.5, 101.7, 68.5, 63.1 (2C), 59.9, 56.4, 51.3 (2C), 26.2, 25.0; HR-ESI-MS (positive mode) *m/z*: 487.1773 [M+H]^+^ (calculated for C_24_H_28_ClN_4_O_5_, 487.1748).

#### 3.2.15. 6-Methoxy-7-(4-Morpholinobutoxy)-3-Nitro-4-(3-Chlorophenylamino)-Quinoline (**11o**)

Yield: 40%, yellow solid. ^1^H-NMR (400 MHz, CDCl_3_) δ: 1.67 (m, 2H, CH_2_), 1.92 (m, 2H, CH_2_), 2.42 (br.s, 6H, 3×NCH_2_), 3.39 (s, 3H, OCH_3_), 3.67 (t, 4H, *J* = 5.6 Hz, 2 × OCH_2_), 4.17 (t, *J* = 6.6 Hz, 2H, OCH_2_), 6.80 (s, 1H, ArH), 6.97 (d, *J* = 8.0 Hz, 1H, ArH), 7.10 (s, 1H, ArH), 7.15 (d, *J* = 8.0 Hz, 1H, ArH), 7.24 (t, *J* = 8.0 Hz, 1H, ArH), 7.30 (s, 1H, ArH), 9.30 (s, 1H, ArH), 10.16 (s, 1H, NH); ^13^C-NMR (100 MHz, CDCl_3_) δ:154.0, 148.8, 148.2, 145.0, 143.9, 142.5, 135.3, 130.5, 129.3, 125.6, 123.2, 121.2, 112.8, 109.8, 105.8, 69.0, 67.0 (2C), 58.4, 55.4, 53.7 (2C), 26.6, 23.0; HR-ESI-MS (positive mode) *m/z*: 487.1753 [M+H]^+^ (calculated for C_24_H_28_ClN_4_O_5_, 487.1748).

#### 3.2.16. 6-Methoxy-7-(4-Morpholinobutoxy)-3-Nitro-4-(4-Chlorophenylamino)-Quinoline (**11p**)

Yield: 42%, yellow solid. ^1^H-NMR (400 MHz, CDCl_3_) δ: 1.73 (br.s, 2H, CH_2_), 1.95 (m, 2H, CH_2_), 2.49 (br.s, 6H, 3×NCH_2_), 3.41 (s, 3H, OCH_3_), 3.75 (br.s, 4H, 2 × OCH_2_), 4.20 (t, *J* = 6.6 Hz, 2H, OCH_2_), 6.81 (s, 1H, ArH), 7.08 (d, *J* = 8.8 Hz, 2H, 2 × ArH), 7.32 (s, 1H, ArH), 7.34 (d, *J* = 8.8 Hz, 2H, 2 × ArH), 9.35 (s, 1H, ArH), 10.24 (s, 1H, NH); ^13^C-NMR (100 MHz, CDCl_3_) δ:153.6, 148.5, 147.9, 145.1, 144.4, 139.8, 131.2, 129.7 (2C), 129.0, 124.7 (2C), 112.8, 109.8, 106.0, 68.5, 65.2 (2C), 58.0, 55.3, 52.8 (2C), 26.3, 22.7; HR-ESI-MS (positive mode) *m/z:* 487.1754 [M+H]^+^ (calculated for C_24_H_28_ClN_4_O_5_, 487.1748).

#### 3.2.17. 6-Methoxy-7-(4-Morpholinobutoxy)-3-Nitro-4-(4-Hydroxyphenylamino)-Quinoline (**11q**)

Yield: 27%, yellow solid. ^1^H-NMR (400 MHz, CDCl_3_) δ: 1.71 (m, 2H, CH_2_), 1.94 (m, 2H, CH_2_), 2.44 (m, 6H, 3×NCH_2_), 3.40 (s, 3H, OCH_3_), 3.73 (t, *J* = 4.5 Hz, 4H, 2 × OCH_2_), 4.19 (t, *J* = 6.4 Hz, 2H, OCH_2_), 6.89 (d, *J* = 8.5 Hz, 2H, 2 × ArH), 6.95 (s, 1H, ArH),7.12 (d, *J* = 8.5 Hz, 2H, 2 × ArH), 7.28 (s, 1H, ArH), 9.35 (s, 1H, ArH), 10.53 (s, 1H, NH); ^13^C-NMR (100 MHz, CDCl_3_) δ:154.8, 153.4, 147.9, 147.7, 146.1, 145.5, 133.6, 128.4, 126.4(2C), 116.6(2C), 112.4, 109.6, 106.5, 68.8, 66.9 (2C), 58.5, 55.2, 53.7 (2C), 26.6, 22.9; HR-ESI-MS (positive mode) *m/z*: 469.2083 [M+H]^+^ (calculated for C_24_H_29_N_4_O_6_, 469.2087).

#### 3.2.18. 6-Methoxy-7-(4-Morpholinobutoxy)-3-Nitro-4-(4-Methoxyphenylamino)-Quinoline (**11r**)

Yield: 38%, yellow solid. ^1^H-NMR (400 MHz, CDCl_3_) δ: 1.72 (m, 2H, H-3″), 1.95 (m, 2H, H-2″), 2.45 (t, *J* = 7.6 Hz, 2H, H-4″), 2.49 (br.s, 4H, H-2‴ and H-6‴), 3.37 (s, 3H, 6-OCH_3_), 3.73 (t, *J* = 4.6 Hz, 4H, H-3‴ and H-5‴), 3.84 (s, 3H, 4′-OCH_3_), 4.19 (t, *J* = 6.6 Hz, 2H, H-1″), 6.93 (s, 1H, H-5), 6.94 (d, *J* = 8.8 Hz, 2H, H-3′ and H-5′), 7.16 (d, *J* = 8.8 Hz, 2H, H-2′ and H-6′), 7.30 (s, 1H, H-8), 9.34 (s, 1H, H-2), 10.52 (s, 1H, NH); ^13^C-NMR (100 MHz, CDCl_3_) δ:158.0, 153.4, 147.94, 147.92, 145.9, 145.5, 133.9, 127.6, 126.1 (2C), 114.9 (2C), 112.4, 109.8, 106.4, 68.8, 66.9 (2C), 58.4, 55.7, 55.2, 53.7 (2C), 26.6, 22.9; HR-ESI-MS (positive mode) *m/z*: 483.2259 [M+H]^+^ (calculated for C_25_H_31_N_4_O_6_, 483.2244).

### 3.3. Synthesis of Compounds ***12a**–**12d***

To a stirred solution of compounds **11a**–**11d** (0.33 mmol) in ethanol (30 mL) was added saturated aqueous ammonium chloride (4.1 mmol) and iron powder (3.6 mmol). The mixture was refluxed for 1.5 h. Then the hot solution was filtered, and the filtrate was evaporated to dryness. The crude material was purified by reversed-phase C_18_ preparative MPLC eluted with MeOH/H_2_O (1:1) to give **12a**–**12d**.

#### 3.3.1. 6-Methoxy-7-(3-Morpholinopropoxy)-3-Amino-4-Phenylamino-Quinoline (**12a**)

Yield: 30%, yellow solid. ^1^H-NMR (400 MHz, CD_3_OD) δ: 2.43 (m, 2H, CH_2_**CH_2_**CH_2_), 3.43 (br.s, 4H, 2 × NCH_2_), 3.45 (t, *J* = 6.8 Hz, 2H, CH_2_N), 3.69 (s, 3H, OCH_3_), 3.99 (br.s, 4H, 2 × OCH_2_), 4.36 (t, *J* = 5.2 Hz, 2H, OCH_2_), 7.06 (d, *J* = 7.6 Hz, 2H, 2 × ArH), 7.15 (t, *J* = 7.6 Hz, 1H, H-4′), 7.19 (s, 1H, ArH), 7.33 (s, 1H, ArH), 7.39 (t, *J* = 7.6 Hz, 2H, 2 × ArH), 8.29 (s, 1H, ArH); ^13^C-NMR (100 MHz, CD_3_OD) δ: 153.2, 151.1, 142.0, 139.3, 132.7, 130.4 (3C), 128.7, 125.0, 122.1 (2C), 117.4, 103.7, 101.2, 68.0, 65.1 (2C), 56.5, 56.4, 53.4 (2C), 24.5; HR-ESI-MS (positive mode) *m/z*: 409.2239 [M+H]^+^ (calculated for C_23_H_29_N_4_O_3_, 409.2240).

#### 3.3.2. 6-Methoxy-7-(3-Morpholinopropoxy)-3-Amino-4-(2-Chlorophenylamino)-Quinoline (**12b**)

Yield: 19%, yellow solid. ^1^H-NMR (400 MHz, CD_3_OD) δ: 2.20 (br.s, 2H, CH_2_**CH_2_**CH_2_), 2.88 (br.s, 4H, 2 × NCH_2_), 2.93 (m, 2H, CH_2_N), 3.73 (s, 3H, OCH_3_), 3.82 (br.s, 4H, 2 × OCH_2_), 4.18 (br.s, 2H, OCH_2_), 6.27 (d, *J* = 7.6 Hz, 1H, ArH), 6.77 (t, *J* = 7.6 Hz, 1H, ArH), 6.93 (s, 1H, ArH), 7.01 (t, *J* = 7.6 Hz, 1H, ArH), 7.25 (s, 1H, ArH), 7.39 (d, 1H, *J* = 7.6 Hz, ArH), 8.40 (s, 1H, ArH); ^13^C-NMR (100 MHz, CD_3_OD) δ: 151.9, 150.3, 142.5, 140.6, 139.5, 138.2, 130.7, 128.8, 127.5, 123.6, 121.8, 121.1, 116.4, 108.9, 101.6, 67.9, 66.6 (2C), 56.7, 56.1, 54.2 (2C), 26.0; HR-ESI-MS (positive mode) *m/z*: 443.1873 [M+H]^+^ (calculated for C_23_H_28_ClN_4_O_3_, 443.1850).

#### 3.3.3. 6-Methoxy-7-(3-Morpholinopropoxy)-3-Amino-4-(3-Chlorophenylamino)-Quinoline (**12c**)

Yield: 30%, yellow solid. ^1^H-NMR (400 MHz, CD_3_OD) δ: 2.45 (br.s, 2H, CH_2_**CH_2_**CH_2_), 3.48 (t, *J* = 6.2 Hz, 2H, CH_2_N), 3.66 (br.s, 4H, 2 × NCH_2_), 3.80 (s, 3H, OCH_3_), 4.10 (br.s, 4H, 2 × OCH_2_), 4.36 (t, br.s, 2H, OCH_2_), 6.90 (d, *J* = 6.4 Hz, 1H, ArH), 6.97 (s, 1H, ArH), 7.07 (d, *J* = 7.8 Hz, 1H, ArH), 7.22 (s, 1H, ArH), 7.32 (br.s, 2H, 2 × ArH), 8.33 (s, 1H, ArH); ^13^C-NMR (100 MHz, CD_3_OD) δ: 153.4, 152.0, 143.5, 137.3, 135.8, 134.5, 132.2, 131.5, 129.4, 123.8, 120.3, 119.3, 118.8, 103.1, 101.2, 68.1, 65.2 (2C), 56.7, 56.6, 53.5 (2C), 24.5; HR-ESI-MS (positive mode) *m/z*: 443.1866 [M+H]^+^ (calculated for C_23_H_28_ClN_4_O_3_, 443.1850).

#### 3.3.4. 6-Methoxy-7-(3-Morpholinopropoxy)-3-Amino-4-(4-Chlorophenylamino)-Quinoline (**12d**)

Yield: 28%, yellow solid. ^1^H-NMR (400 MHz, CD_3_OD) δ: 2.42 (m, 2H, CH_2_**CH_2_**CH_2_), 3.36 (br.s, 4H, 2 × NCH_2_), 3.38 (t, *J* = 7.2 Hz, 2H, CH_2_N), 3.77 (s, 3H, OCH_3_), 4.00 (br.s, 4H, 2 × OCH_2_), 4.33 (t, *J* = 5.2 Hz, 2H, OCH_2_), 6.92 (d, *J* = 8.4 Hz, 2H, ArH), 7.18 (s, 1H, ArH), 7.29 (d, *J* = 8.4 Hz, 2H, ArH), 7.31 (s, 1H, ArH), 8.28 (s, 1H, ArH); ^13^C-NMR (100 MHz, CD_3_OD) δ: 152.6, 151.3, 140.6, 136.7, 133.4, 133.0, 130.0, 129.9 (2C), 128.7, 121.6 (2C), 118.7, 102.9, 101.9, 67.6, 64.9 (2C), 56.5, 56.2, 53.2 (2C), 24.4; HR-ESI-MS (positive mode) *m/z*: 443.1865 [M+H]^+^ (calculated for C_23_H_28_ClN_4_O_3_, 443.1850).

### 3.4. Biological Evaluation

#### 3.4.1. AChE and BChE Inhibition Assay

AChE and BChE inhibitory activities of compounds were determined by using a modified Ellman’s method [16,22]. AChE (EC 3.1.1.7, from electric eel), BChE (EC 3.1.1.8, from horse serum), 5,5′-dithiobis(2nitrobenzoic acid) (DTNB), acetylthiocholine iodide (ATCI) and butyrylthiocholine iodide (BTCI) were purchased from Sigma-Aldrich. The reaction mixture contained 140 μL of 100 mM sodium phosphate buffer (pH 8.0), the enzyme solution (either 0.05 U/mL of AChE, 20 μL; or 0.05 U/mL of BChE, 20 μL), and test compound (20 μL). Assayed solutions of test compounds were pre-incubated with corresponding ChE at 25 °C for 15 min. The reaction was initiated by the addition of 10 μL of 10 mM DTNB and 10 μL of 7.5 mM substrate (ATCI or BTCI). The activity was determined by measuring the increase in absorbance at 412 nm at 37 °C in 10 min intervals using a microplate reader. The percentage of inhibition was calculated from the measured data as follows: (Ac − Ai)/Ac × 100%, where Ai and Ac represent the change in the absorbance in the presence of inhibitor and without inhibitor, respectively.

#### 3.4.2. Enzyme Kinetic Analysis against AChE

In order to investigate the reversibility of compounds **11a** and **11g** to AChE, different concentrations of compounds (0−10 μM) and five different concentrations of AChE (0.025−0.10 U/mL) were measured at 412 nm after a 2 min incubation at 37 °C. Values were measured in triplicate for the velocity and enzyme concentration profiles. Velocity (V) was calculated from the measured data as follows: V = (Ac−Ai)/2, where Ai and Ac represent the change in absorbance in the presence and absence of AChE, respectively. All data were analyzed by OriginPro 2021.

For the estimation of the inhibition model and the inhibition constant *K*_i_, the rate of enzymatic reaction was obtained using different concentrations of the compound (0.0–10 μM) and at least five different concentrations of ATCI (3.75–15.00 mM). For each experiment, the reaction was started by adding substrate and the progression curve was recorded at 412 nm after two minutes of incubation at 37°C. Next, a double inverse plot (1/v vs. 1/[s]) was plotted using the slope of the progression curve to obtain the type of inhibition. The slopes of these inverse plots were then plotted against the compound concentrations in the correlation analysis, and *K*_i_ was determined as the intercept on the negative *x*-axis. All rate measurements were performed in triplicate and data analysis was performed using OriginPro 2021.

#### 3.4.3. ABTS Radical-Scavenging Activity

ABTS radical-scavenging activity was measured by using the modified method [30,31]. The test compound was dissolved in ethanol as 1.0 mg/mL, 0.5 mg/mL, 0.25 mg/mL, 0.125 mg/mL, 0.0625 mg/mL, and 0.03125 mg/mL. Similarly, the ethanol solution of Trolox with the same concentration gradient was prepared as a positive control. ABTS solution at a concentration of 7 mM and potassium persulfate solution at a concentration of 2.45 mM with distilled water were also prepared. The ABTS solution and the potassium persulfate solution were mixed in equal amounts, and then placed in the dark and protected from light for 12 h. The ABTS standard solution was diluted with ethanol until the absorbance value at 734 nm was in the range of 0.7 ± 0.05. Then, 3900 µL of ABTS cation solution was mixed with 100 µL of test compound at different concentrations. The samples were kept in the dark for 10 min at room temperature and absorption was measured at 734 nm on a spectrophotometer. The percentage of ABTS radical-scavenging activity was calculated as follows: (Ac−Ai)/Ac × 100%, where Ai and Ac represented the change in absorbance in the presence and absence of the test sample, respectively.

### 3.5. Docking Study

#### 3.5.1. Molecular Docking Studies on AChE

The binding modes were generated by using the Discovery Studio CDOCKER software (Accelrys, San Diego, CA, USA). The crystal structure of human AChE (hAChE) in complex with donepezil (PDB code 4EY7) was taken from Protein Data Bank. The simulation was performed as our described previously [32].

#### 3.5.2. Molecular Docking Studies on BChE

Flexible docking was conducted using Discovery Studio 2017 R2 (Accelrys, San Diego, USA). The crystal structure of BChE from Homo sapiens (Code ID: 4BDS) was extracted from the Protein Data Bank. The detailed procedure could reference our previous work [16].

### 3.6. Solubility Measurement

Solubility was determined by using the shake-flask method [33].The desired amount of the compound was placed in a test tube, to which an appropriate amount of double distilled water was introduced. The suspension was agitated on a plate shaker at 37 °C for 24 h. After centrifuging, HPLC analyses of supernatant was performed. The solubility of the compound was obtained from its HPLC result.

### 3.7. Lipophilicity Determination

The lipophilicities of test compounds were determined using the traditional shake-flask method [34]. A solid test compound (2 mg) was placed into a 10 mL pear-shaped flask, and measured volumes of octanol and water were added for log*P* determination. The resulting biphasic mixture was stirred for 24 h and then left without stirring for 12 h to allow phase separation. The test compound in each phase was sampled and quantitated using HPLC. The areas under the peaks were divided and the log taken.

## 4. Conclusions

In summary, a novel series of morpholine-bearing quinoline derivatives were designed, synthesized, and evaluated as multifunctional agents for the treatment of AD. Among them, compounds **11a**, **11g**, **11h**, **11j**, **11l,** and **12a** showed considerable inhibitory activity against AChE. Especially, compound **11g** demonstrated the most potent inhibition to AChE and BChE with IC_50_ values of 1.94 μM and 28.37 μM, respectively. The kinetic analysis demonstrated both compounds **11a** and **11g** acted as mixed-type AChE inhibitors. A further docking comparison between the **11a**- and **12a**-AChE complexes agreed with the different inhibitory potencies we observed in experiments. Besides, compounds **11e**, **11f**, **11g**, **11k**, **11l**, **11q,** and **11r** had distinct ABTS radical-scavenging activities. More importantly, both the compounds **11f** and **11l** exhibited higher free radical-scavenging activities than Trolox. As for as the ability to cross the BBB, compounds **11a**, **11g**, **12a**, and **12c** exhibited a suitable solubility and lipophilicity to facilitate brain penetration. Overall, comprehensively considering drug-like properties and ChE inhibition, we conclude that the most balanced compounds are **11a**, **11g**, **12a**, and **12c**. In the light of our current study, we are planning to continue the synthesis of more modified 3-aminoquinoline molecules as a novel class of AChE and BChE inhibitors.

## Figures and Tables

**Figure 2:**
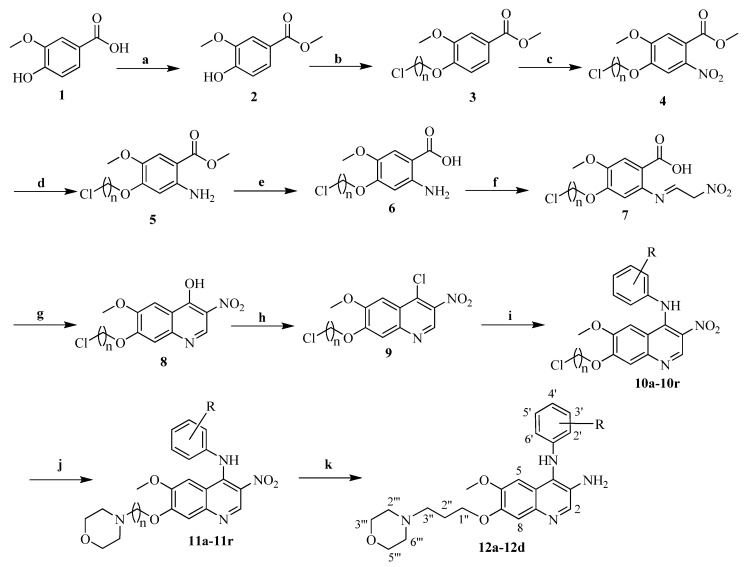
Synthesis of compounds **11a**–**11r** and **12a**–**12d**. Reagents and conditions: (a) MeOH, HCl, 70 °C, 10 h; (b) Br(CH_2_)_n_Cl (n = 2, 3, 4), K_2_CO_3_, acetone, 70 °C, 10 h; (c) fuming HNO_3_, CH_2_Cl_2_, room temperature, 6 h; (d) Fe, NH_4_Cl, EtOH, reflux; (e) 5% NaOH, EtOH, 50 °C, 10 h; (f) HCl, HON=CHCH_2_NO_2_, room temperature, 18 h; (g) KOAc, Ac_2_O, 15 min, reflux; (h) POCl_3_, 70 °C, 10 h; (i) corresponding aniline, isopropanol, 90 °C, 12 h; (j) morpholine, NaI, K_2_CO_3_, CH_3_CN, 87 °C, 24 h, reflux; (k) Fe, NH_4_Cl, EtOH, reflux.

**Table 1:** Inhibitory activities of cholinesterase along with physicochemical properties of compounds.

Compd.	n	R	AChE IC_50_ (μM) ^a^	BChE IC_50_ (μM) ^a^	SI ^b^	Log*P*	TPSA (Å^2^)	MW
**11a**	3	H	4.94 ± 0.15	80.91 ± 2.31	16.37	3.41	107.13	438.19
**11b**	3	2-Cl	12.51 ± 0.21	130.38 ± 4.62	10.42	4.03	107.13	472.15
**11c**	3	3-Cl	32.40 ± 0.16	114.12 ± 5.36	3.55	4.03	107.13	472.15
**11d**	3	4-Cl	88.68 ± 1.10	82.39 ± 3.06	0.92	4.03	107.13	472.15
**11e**	3	4-OH	34.47 ± 0.09	>150	>4.35	3.02	127.36	454.19
**11f**	3	4-OCH_3_	12.80 ± 0.55	96.60 ± 4.75	7.54	3.54	116.36	468.20
**11g**	2	H	1.94 ± 0.13	28.37 ± 1.85	16.30	2.96	107.13	424.17
**11h**	2	2-Cl	6.46 ± 0.77	81.08 ± 4.03	12.55	3.58	107.13	458.14
**11i**	2	3-Cl	10.01 ± 0.52	110.96 ± 5.59	11.08	3.58	107.13	458.14
**11j**	2	4-Cl	8.63 ± 0.40	76.81 ± 3.76	8.90	3.58	107.13	458.14
**11k**	2	4-OH	18.49 ± 1.74	>150	>8.11	2.57	127.36	440.17
**11l**	2	4-OCH_3_	9.08 ± 0.53	63.92 ± 2.87	7.03	3.09	116.36	454.19
**11m**	4	H	13.34 ± 0.07	>150	>11.24	3.87	107.13	452.21
**11n**	4	2-Cl	85.57 ± 0.04	>150	>1.75	4.49	107.13	486.17
**11o**	4	3-Cl	>150	116.25 ± 5.36	<0.77	4.49	107.13	486.17
**11p**	4	4-Cl	90.46 ± 0.06	98.10 ± 0.02	1.08	4.49	107.13	486.17
**11q**	4	4-OH	36.81 ± 0.45	>150	>4.07	3.48	127.36	468.20
**11r**	4	4-OCH_3_	18.92 ± 0.51	106.49 ± 1.16	5.65	5.86	116.36	482.22
**12a**	3	H	9.13 ± 0.42	30.53 ± 2.48	3.34	2.23	81.34	408.22
**12b**	3	2-Cl	43.28 ± 0.68	130.10 ± 3.76	3.01	2.79	81.34	442.18
**12c**	3	3-Cl	22.06 ± 0.33	27.84 ± 2.16	1.26	2.79	81.34	442.18
**12d**	3	4-Cl	21.84 ± 0.20	145.00 ± 4.25	6.63	2.79	81.34	442.18
**A**			0.92 ± 0.05 ^c^	14.20 ± 0.96 ^c^	15.43 ^c^	−3.25 ^d^	100.56	501.36
**B**			0.86 ± 0.03 ^e^	2.65 ± 0.52 ^e^	3.08 ^e^	4.58	97.90	506.98
**C**			1.20 ± 0.18 ^f^	18.52 ± 1.2 ^f^	15.43 ^f^	5.20	107.13	480.56
Galanthamine			1.28 ± 0.01	24.41 ± 2.01	19.07	1.41	41.93	287.15

^a^ IC_50_ values are at least from three independents and are expressed as the means ± SD. ^b^ SI for AChE = IC_50_ BChE/IC_50_ AChE. ^c^ From Ref. [18]. ^d^ Log*P* value was estimated in http://www.swissadme.ch according to Ref. [23] on 30 July 2022. ^e^ From Ref. [17]. ^f^ From Ref. [16].

**Table 2:** Antioxidant activities of bioactive derivatives in ABTS radical-scavenging assay.

Compd.	IC_50_ (μg/mL)	IC_50_ (μM)
**11e**	12.01 ± 1.49	26.43 ± 3.28
**11f**	4.25 ± 0.63	9.07 ± 1.34
**11g**	17.50 ± 2.71	41.23 ± 6.38
**11k**	5.75 ± 1.37	13.55 ± 3.23
**11l**	2.75 ± 0.53	6.05 ± 1.17
**11q**	26.12 ± 2.13	55.75 ± 4.55
**11r**	11.25 ± 1.27	23.33 ± 2.63
Trolox	2.76 ± 0.19	11.03 ± 0.76

## Data Availability

Data are contained within the article and supplementary material.

## Supplementary Materials

The following supporting information can be downloaded at: https://www.mdpi.com/article/10.3390/ijms231911231/s1.
