# Supplementary material for: Novel Morpholine-Bearing Quinoline Derivatives as Potential Cholinesterase Inhibitors: The Influence of Amine, Carbon Linkers and Phenylamino Groups
Source: Int J Mol Sci. 2022 Sep 23;23(19):11231. doi: 10.3390/ijms231911231 (PMC9570490; doi:10.3390/ijms231911231)

Novel Morpholine-Bearing Quinoline Derivatives as Potential Cholinesterase Inhibitors: The Influence of Amine, Carbon Linkers and Phenylamino Groups

Cheng Liu [1], Li-Ning Wang [2] and Yu-Ming Liu[1,3,*]

1 Department of Pharmacy Engineering, Tianjin University of Technology, Tianjin 300384, China

2 College of Traditional Chinese Medicine, Tianjin University of Traditional Chinese Medicine, Tianjin 300193, China

3 Tianjin Key Laboratory of Drug Targeting and Bioimaging, Tianjin University of Technology, Tianjin 300384, China

*** Corresponding author:**

Yu-Ming Liu,

Department of Pharmacy Engineering, Tianjin University of Technology, Tianjin, 300384, PR China.

E-mail address: coumarin1968@hotmail.com (Y.-M. Liu).

Table of Contents

Figure S1. Spectroscopic data of compounds **10a–10e** 1
Figure S2. HR-ESI-MS, ^{1}H NMR, and ^{13}C NMR spectra of compound **10a** 2
Figure S3. HR-ESI-MS, ^{1}H NMR, and ^{13}C NMR spectra of compound **10b** 3
Figure S4. HR-ESI-MS, ^{1}H NMR, and ^{13}C NMR spectra of compound **10c** 4
Figure S5. HR-ESI-MS, ^{1}H NMR, and ^{13}C NMR spectra of compound **10d** 5
Figure S6. HR-ESI-MS, ^{1}H NMR, and ^{13}C NMR spectra of compound **10e** 6
Figure S7. HR-ESI-MS, ^{1}H NMR, and ^{13}C NMR spectra of compound **11a** 7
Figure S8. HR-ESI-MS, ^{1}H NMR, and ^{13}C NMR spectra of compound **11b** 8
Figure S9. HR-ESI-MS, ^{1}H NMR, and ^{13}C NMR spectra of compound **11c** 9
Figure S10. HR-ESI-MS, ^{1}H NMR, and ^{13}C NMR spectra of compound **11d** 10
Figure S11. HR-ESI-MS, ^{1}H NMR, and ^{13}C NMR spectra of compound **11e** 11
Figure S12. HR-ESI-MS, ^{1}H NMR, ^{13}C NMR and NOESY spectra of compound **11f** 12-13
Figure S13. HR-ESI-MS, ^{1}H NMR, and ^{13}C NMR spectra of compound **11g** 14
Figure S14. HR-ESI-MS, ^{1}H NMR, and ^{13}C NMR spectra of compound **11h** 15
Figure S15. HR-ESI-MS, ^{1}H NMR, and ^{13}C NMR spectra of compound **11i** 16
Figure S16. HR-ESI-MS, ^{1}H NMR, and ^{13}C NMR spectra of compound **11j** 17
Figure S17. HR-ESI-MS, ^{1}H NMR, and ^{13}C NMR spectra of compound **11k** 18
Figure S18. HR-ESI-MS, ^{1}H NMR, ^{13}C NMR and NOESY spectra of compound **11l** 19-20
Figure S19. HR-ESI-MS, ^{1}H NMR, and ^{13}C NMR spectra of compound **11m** 21
Figure S20. HR-ESI-MS, ^{1}H NMR, and ^{13}C NMR spectra of compound **11n** 22
Figure S21. HR-ESI-MS, ^{1}H NMR, and ^{13}C NMR spectra of compound **11o** 23
Figure S22. HR-ESI-MS, ^{1}H NMR, and ^{13}C NMR spectra of compound **11p** 24
Figure S23. HR-ESI-MS, ^{1}H NMR, and ^{13}C NMR spectra of compound **11q** 25
Figure S24. HR-ESI-MS, ^{1}H NMR, ^{13}C NMR and NOESY spectra of compound **11r** 26-27
Figure S25. HR-ESI-MS, ^{1}H NMR, and ^{13}C NMR spectra of compound **12a** 28
Figure S26. HR-ESI-MS, ^{1}H NMR, and ^{13}C NMR spectra of compound **12b** 29
Figure S27. HR-ESI-MS, ^{1}H NMR, and ^{13}C NMR spectra of compound **12c** 30
Figure S28. HR-ESI-MS, ^{1}H NMR, and ^{13}C NMR spectra of compound **12d** 31

Spectroscopic data of compounds **10a–10e**

*7-(3-chloropropoxy)-6-methoxy-3-nitro-4-phenylamino-quinoline (**10a**).*

Yellow solid. ^{1}H-NMR (400 MHz, DMSO-d_6) δ: 2.26 (m, 2H, CH_2**CH_2**CH_2), 3.69 (s, 3H, OCH_3), 3.81 (t, *J* = 6.5 Hz, 2H, CH_2Cl), 4.28 (t, *J* = 6.0 Hz, 2H, OCH_2), 7.09 (d, *J* = 7.6 Hz, 2H, H-2′ and H-6′), 7.12 (t, *J* = 7.6 Hz, 1H, H-4′), 7.34 (t, *J* = 7.6 Hz, 2H, H-3′ and H-5′), 7.39 (s, 1H, H-5), 7.49 (s, 1H, H-8), 8.96 (s, 1H, H-2), 9.82 (s, 1H, NH); ^{13}C-NMR (100 MHz, DMSO-d_6) δ: 153.2, 149.4, 146.9, 145.0, 142.0, 141.9, 129.9, 129.7 (2C), 124.8, 121.1 (2C), 115.0, 109.9, 104.5, 65.8, 56.1, 42.3, 31.9; HR-ESI-MS (positive mode) *m*/*z*: 388.1087 $[M+H]^+$ (calculated for $C_{19}H_{19}ClN_3O_4$, 388.1064).

*7-(3-chloropropoxy)-6-methoxy-3-nitro-4-(2-chlorophenylamino)-quinoline (**10b**).*

Yellow solid. ^{1}H-NMR (400 MHz, DMSO-d_6) δ: 2.26 (m, 2H, CH_2**CH_2**CH_2), 3.64 (s, 3H, OCH_3), 3.81 (t, *J* = 6.4 Hz, 2H, CH_2Cl), 4.30 (t, *J* = 6.0 Hz, 2H, OCH_2), 7.19 (d, *J* = 7.7 Hz, 1H, ArH), 7.24 (t, *J* = 7.7 Hz, 1H, ArH), 7.31 (t, *J* = 7.7 Hz, 1H, ArH), 7.38 (s, 1H, H-5), 7.42 (s, 1H, H-8), 7.59 (d, *J* = 7.7 Hz, 1H, ArH), 8.99 (s, 1H, H-2), 9.71 (s, 1H, NH); ^{13}C-NMR (100 MHz, DMSO-d_6) δ: 155.5, 151.7, 149.3, 147.1, 145.2, 141.0, 132.9, 132.1, 130.8, 130.4, 129.5, 127.5, 116.7, 112.4, 106.5, 68.1, 58.3, 44.5, 34.1; HR-ESI-MS (positive mode) *m*/*z*: 422.0662 $[M+H]^+$ (calculated for $C_{19}H_{18}Cl_2N_3O_4$, 422.0674).

*7-(3-chloropropoxy)-6-methoxy-3-nitro-4-(3-chlorophenylamino)-quinoline (**10c**).*

Yellow solid. ^{1}H-NMR (400 MHz, DMSO-d_6) δ: 2.27 (m, 2H, CH_2**CH_2**CH_2), 3.80 (s, 3H, OCH_3), 3.82 (t, *J* = 6.5 Hz, 2H, CH_2Cl), 4.30 (t, *J* = 6.0 Hz, 2H, OCH_2), 6.97 (dd, *J* = 7.9, 1.1 Hz, 1H, ArH), 7.12 (d, *J* = 7.9 Hz, 1H, ArH), 7.13 (d, *J* = 1.1 Hz, 1H, H-2′), 7.31 (t, *J* = 7.9 Hz, 1H, H-5′), 7.42 (s, 1H, H-5), 7.58 (s, 1H, H-8), 8.96 (s, 1H, H-2), 9.80 (s, 1H, NH); ^{13}C-NMR (100 MHz, DMSO-d_6) δ: 154.4, 150.4, 143.5, 142.6, 142.3, 140.5, 134.1, 131.3, 129.6, 125.5, 120.9, 119.4, 115.7, 105.3, 104.8, 66.3, 57.1, 42.2, 31.7; HR-ESI-MS (positive mode) *m*/*z*: 422.0679 $[M+H]^+$ (calculated for $C_{19}H_{18}Cl_2N_3O_4$, 422.0674).

*7-(3-chloropropoxy)-6-methoxy-3-nitro-4-(4-chlorophenylamino)-quinoline (**10d**).*

Yellow solid. ^{1}H-NMR (400 MHz, DMSO-d_6) δ: 2.26 (m, 2H, CH_2**CH_2**CH_2), 3.78 (s, 3H, OCH_3), 3.81 (t, *J* = 6.4 Hz, 2H, CH_2Cl), 4.28 (t, *J* = 5.8 Hz, 2H, OCH_2), 7.07 (d, *J* = 8.6 Hz, 2H, H-2′ and H-6′), 7.35 (d, *J* = 8.6 Hz, 2H, H-3′ and H-5′), 7.39 (s, 1H, H-5), 7.56 (s, 1H, H-8), 8.94 (s, 1H, H-2), 9.80 (s, 1H, NH); ^{13}C-NMR (100 MHz, DMSO-d_6) δ: 153.3, 149.7, 146.9, 144.9, 141.2, 141.0, 130.3, 129.5(2C), 128.0, 122.0(2C), 115.4, 109.9, 104.1, 65.8, 56.3, 42.3, 31.9; HR-ESI-MS (positive mode) *m*/*z*: 422.0691 $[M+H]^+$ (calculated for $C_{19}H_{18}Cl_2N_3O_4$, 422.0674).

*7-(3-chloropropoxy)-6-methoxy-3-nitro-4-(4-hydroxylphenylamino)-quinoline (**10e**).*

Yellow solid. ^{1}H-NMR (400 MHz, DMSO-d_6) δ: 2.24 (m, 2H, CH_2**CH_2**CH_2), 3.59 (s, 3H, OCH_3), 3.80 (t, *J* = 6.4 Hz, 2H, CH_2Cl), 4.24 (t, *J* = 5.9 Hz, 2H, OCH_2), 6.78 (d, *J* = 8.6 Hz, 2H, ArH), 7.01 (d, *J* = 8.6 Hz, 2H, ArH), 7.28 (s, 1H, H-5), 7.35 (s, 1H, H-8), 8.92 (s, 1H, H-2), 9.94 (s, 1H, NH); ^{13}C-NMR (100 MHz, DMSO-d_6) δ: 155.7, 152.8, 148.6, 146.6, 145.1, 144.0, 132.8, 128.2, 124.7(2C), 116.3(2C), 113.8, 109.8, 105.2, 65.7, 55.8, 42.3, 31.9; HR-ESI-MS (positive mode) *m*/*z*: 404.1016 $[M+H]^+$ (calculated for $C_{19}H_{19}ClN_3O_5$, 404.1013).

Compound 10a

HR-ESI-MS $[M+H]^+$

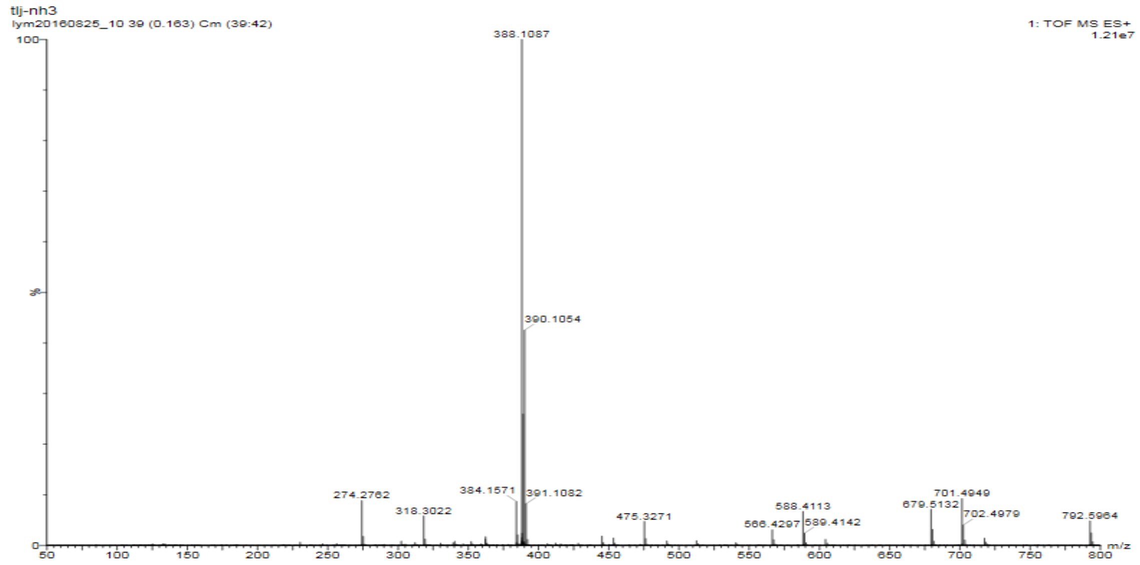

^{1}H-NMR

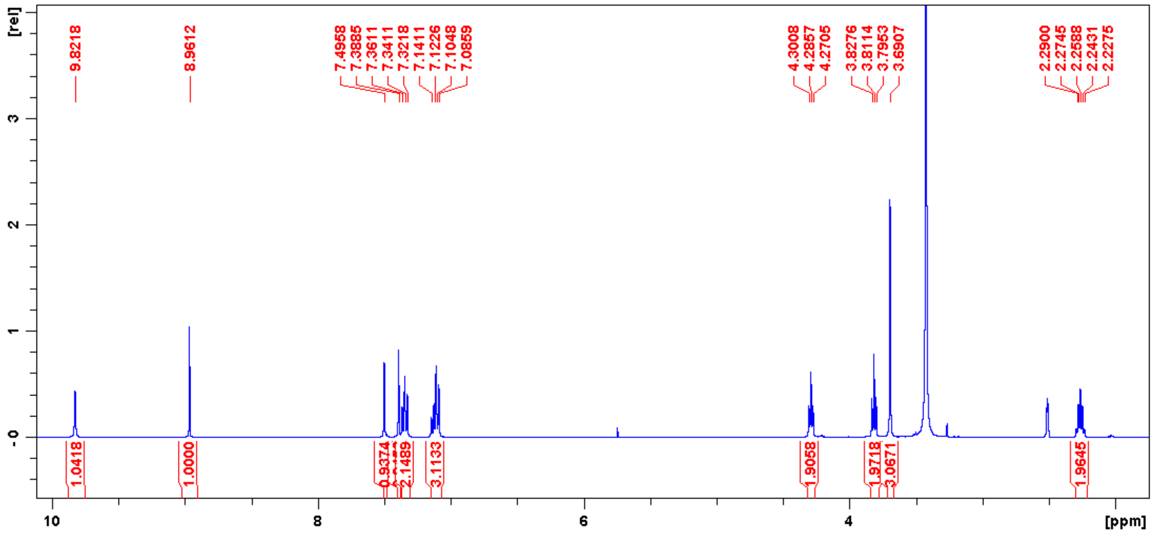

^{13}C-NMR

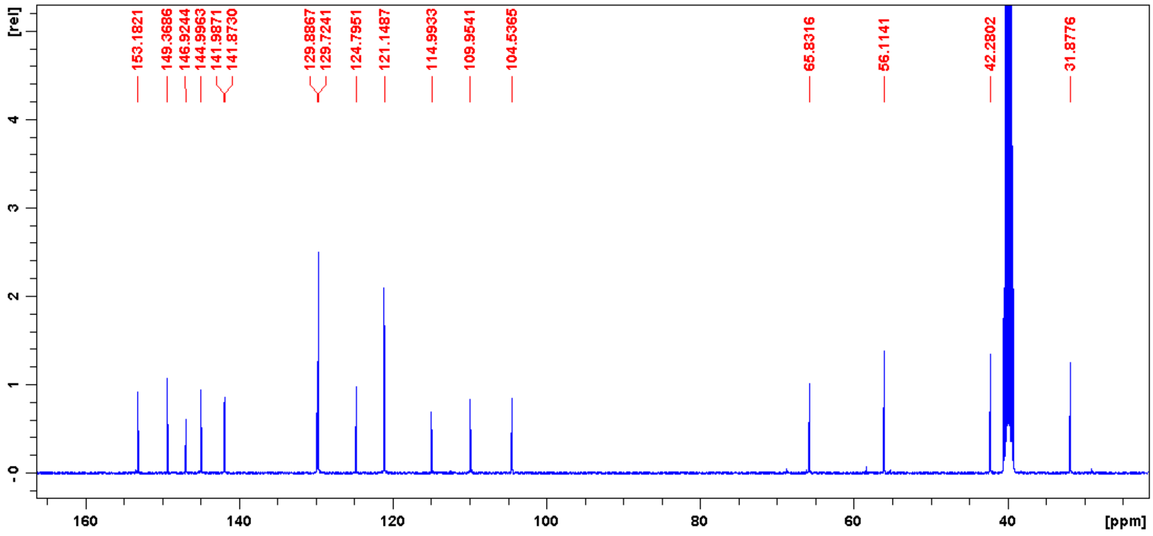

Compound 10b

HR-ESI-MS $[M+H]^+$

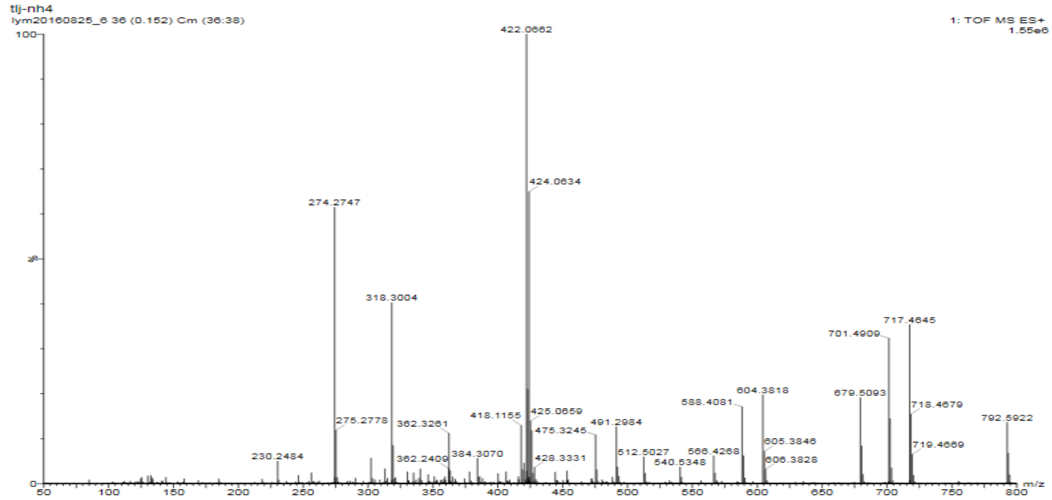

^{1}H-NMR

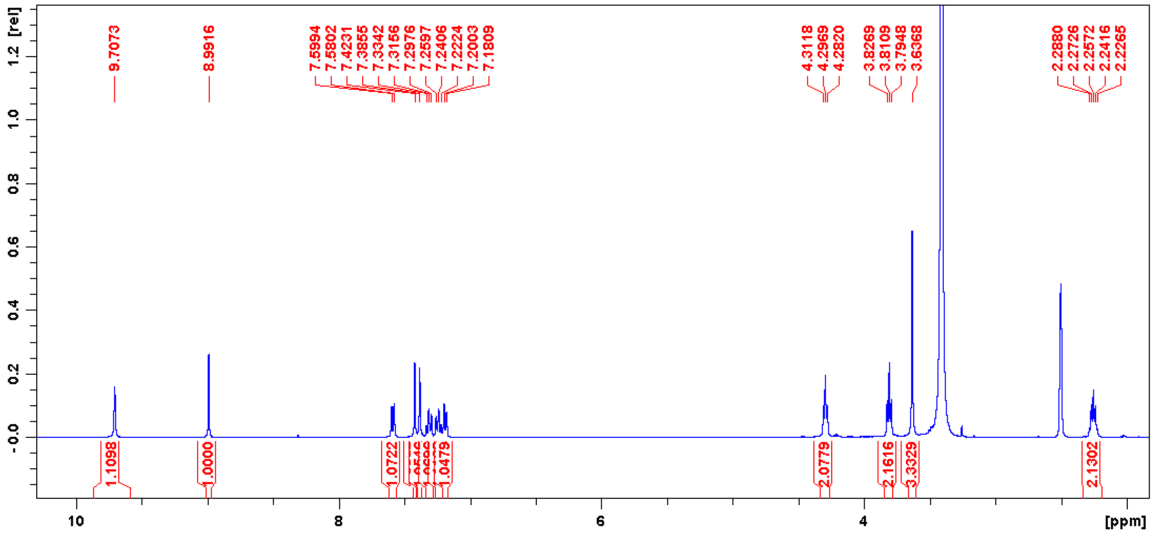

^{13}C-NMR

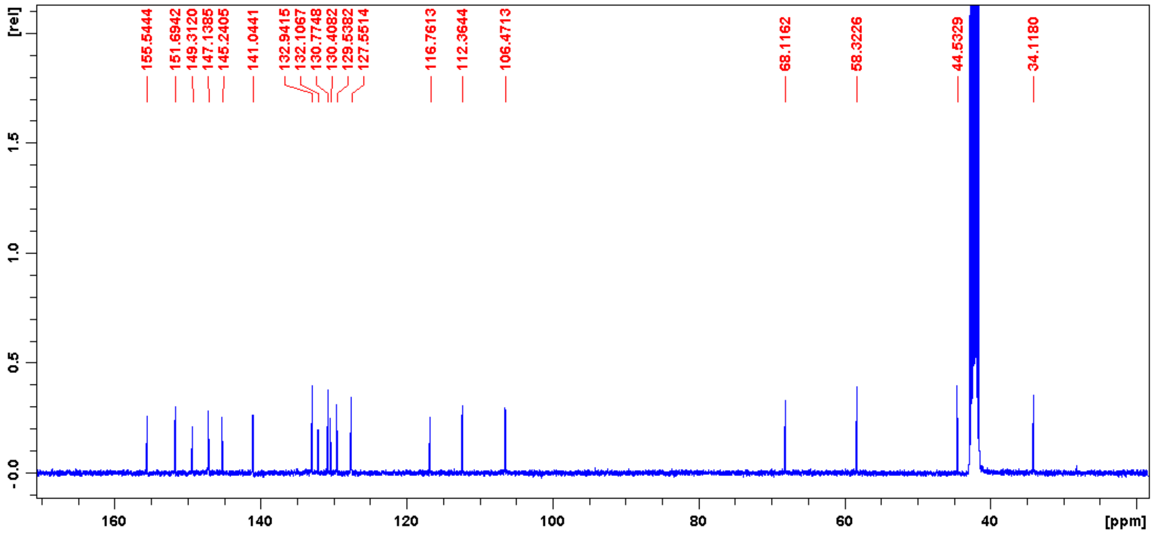

Compound 10c

HR-ESI-MS [M+H]$^+$

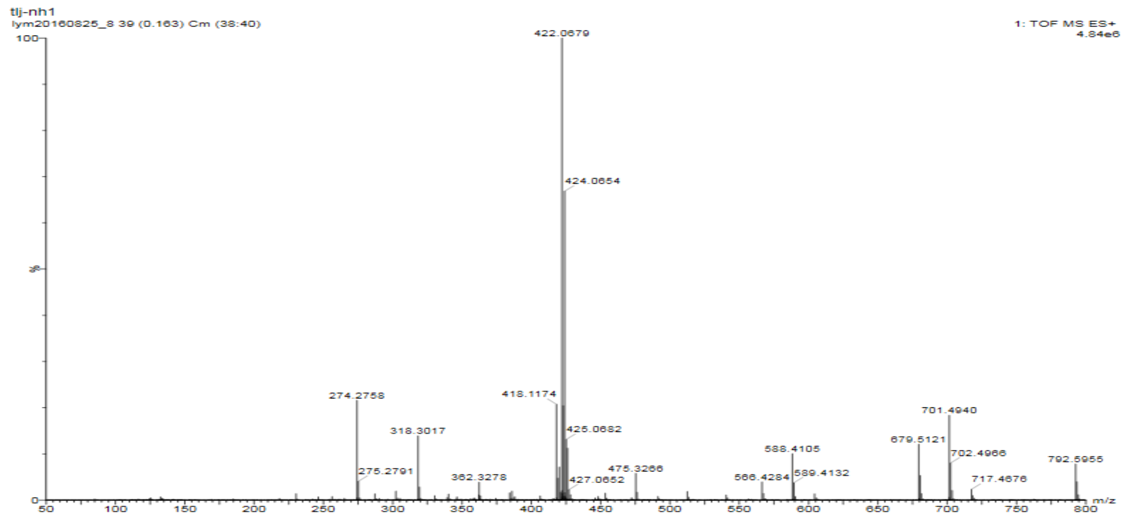

^{1}H-NMR

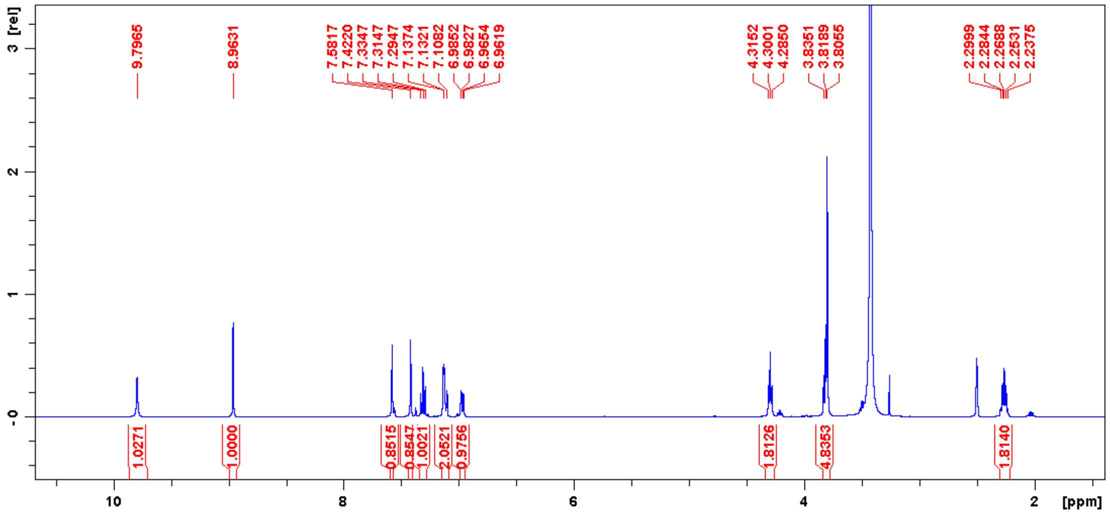

^{13}C-NMR

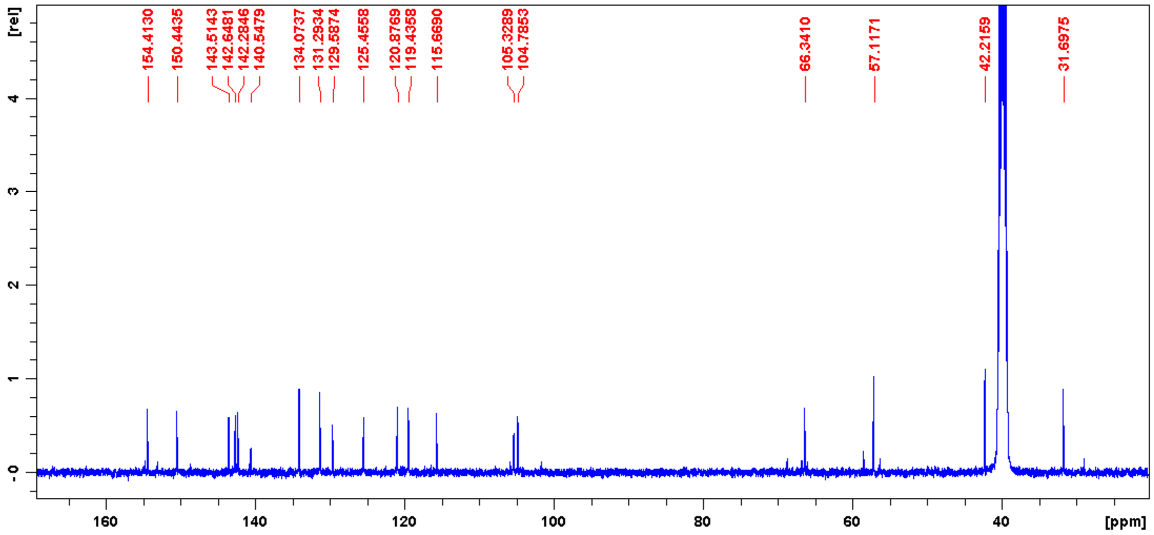

Compound 10d

HR-ESI-MS [M+H]$^+$

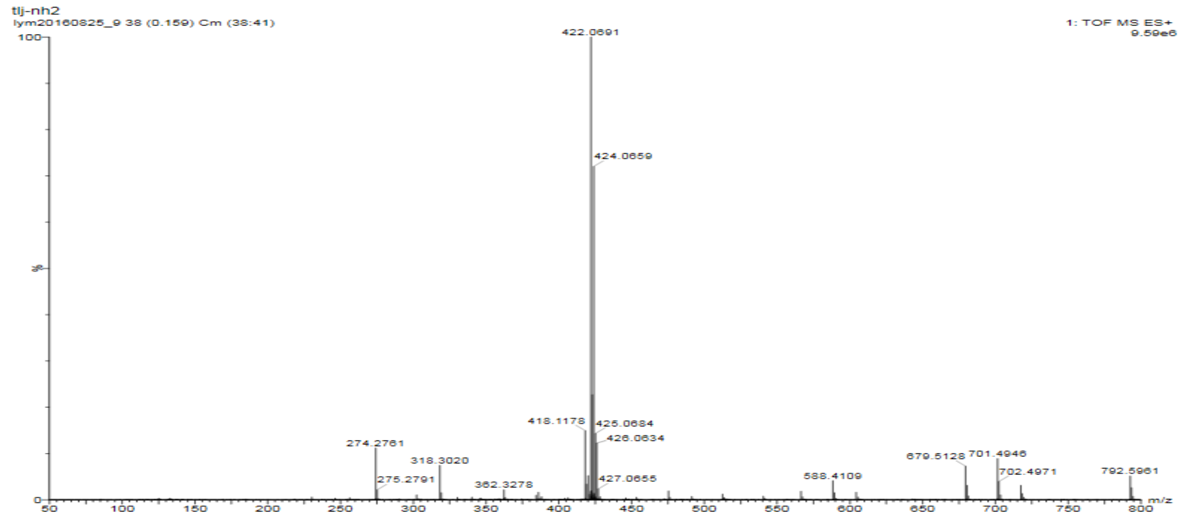

^{1}H-NMR

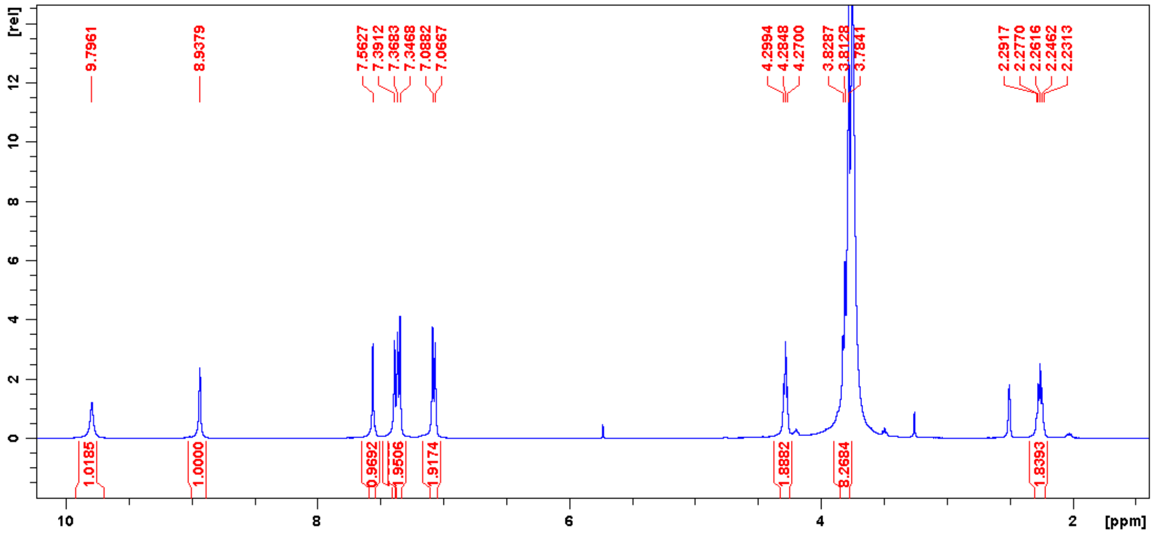

^{13}C-NMR

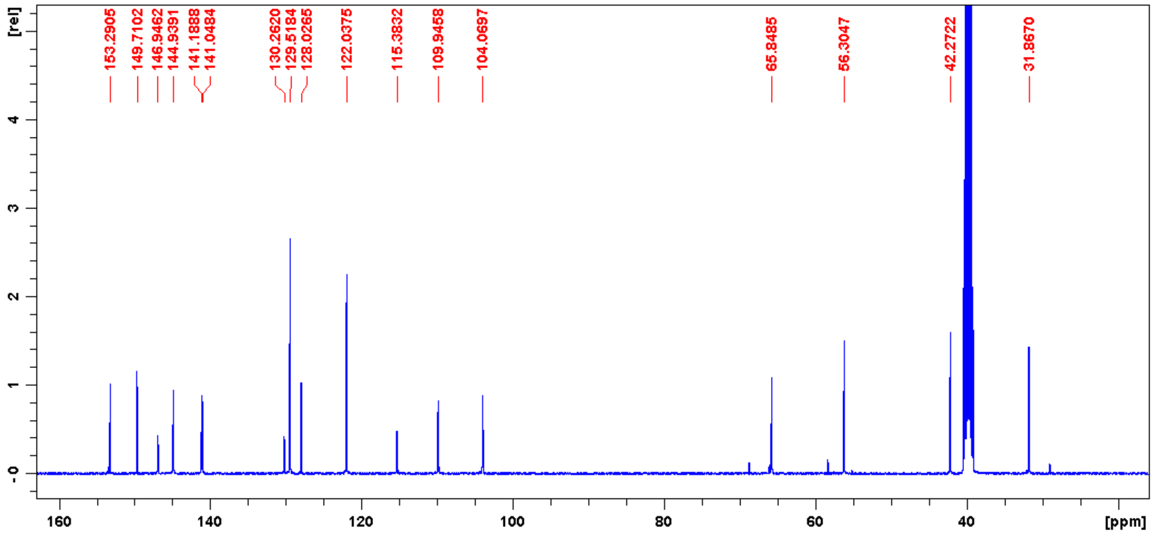

Compound 10e

HR-ESI-MS $[M+H]^+$

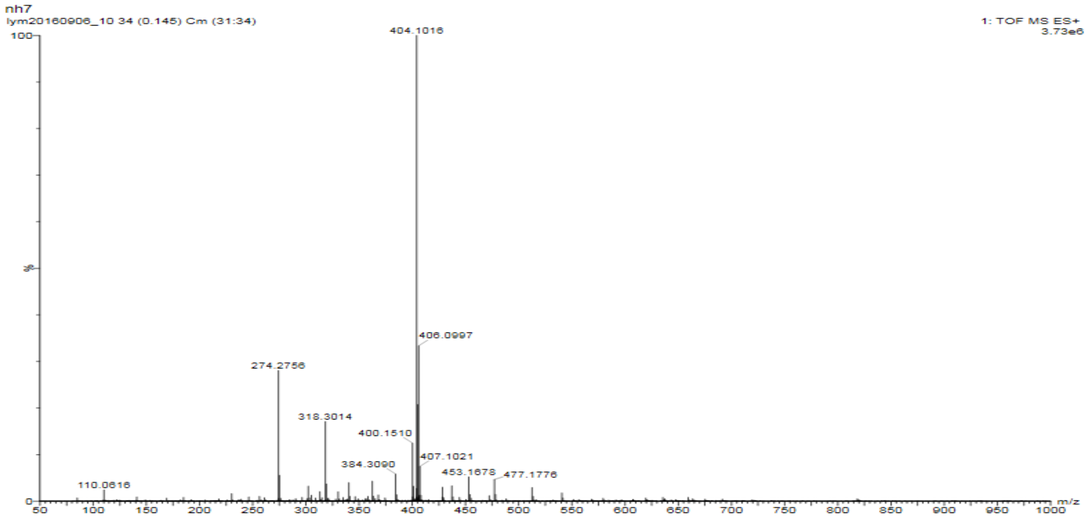

^{1}H-NMR

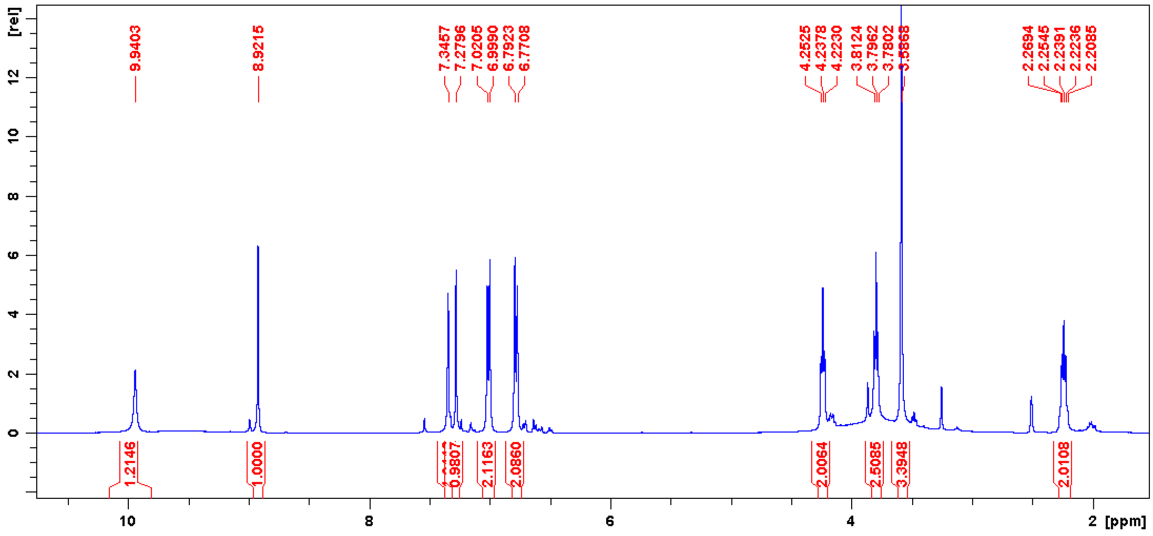

^{13}C-NMR

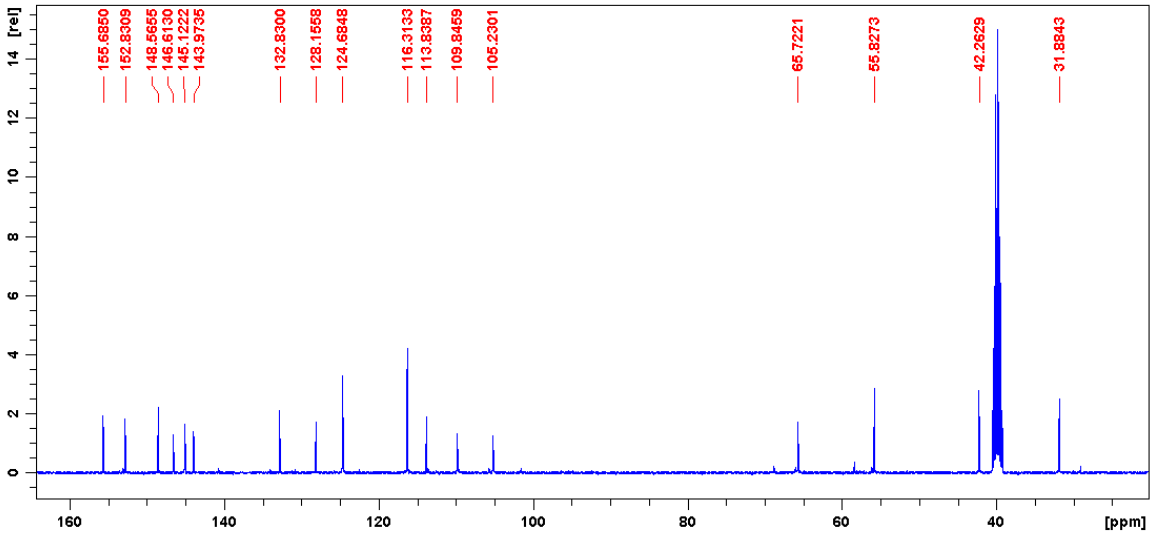

Compound 11a

HR-ESI-MS $[M+H]^+$:

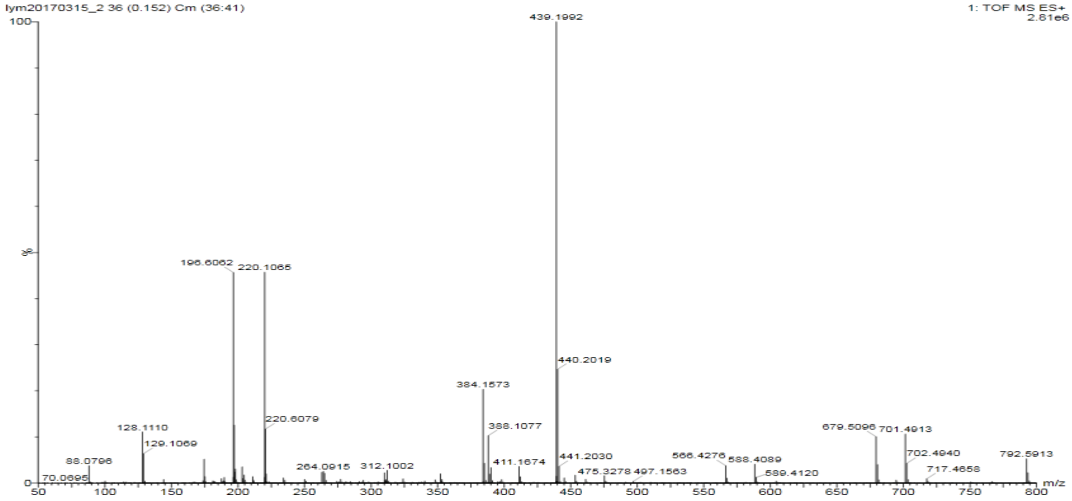

^{1}H-NMR:

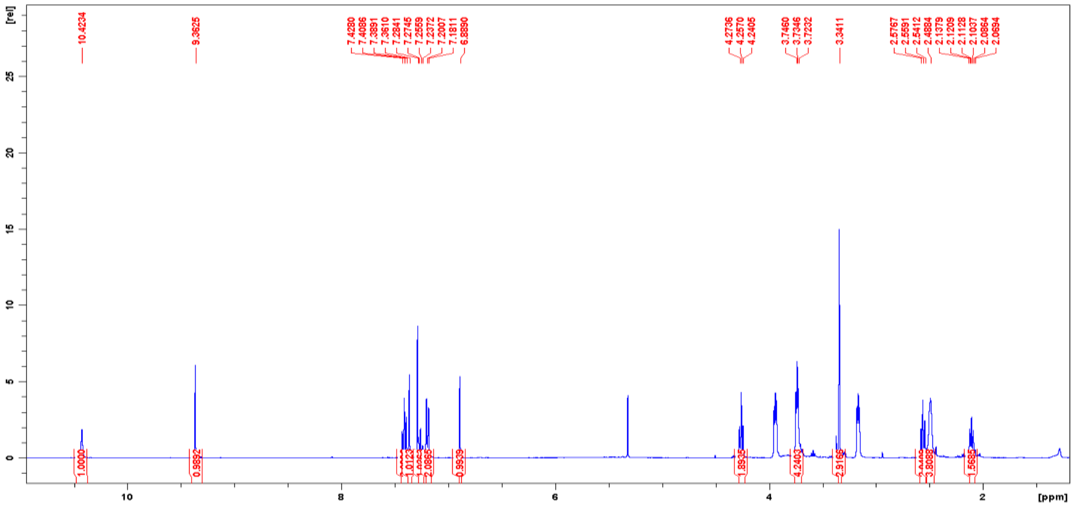

^{13}C-NMR:

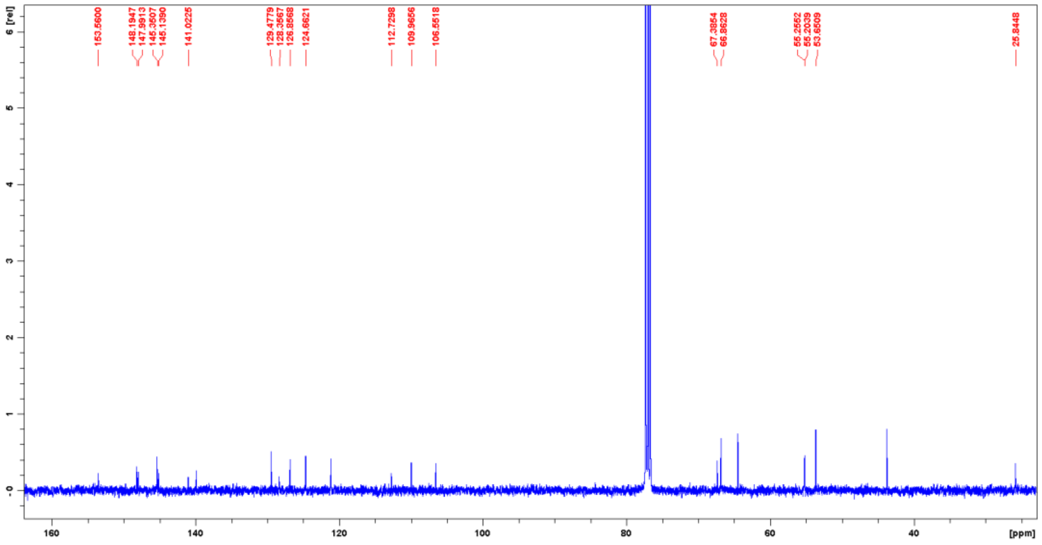

Compound 11b

HR-ESI-MS [M+H]$^{+}$:

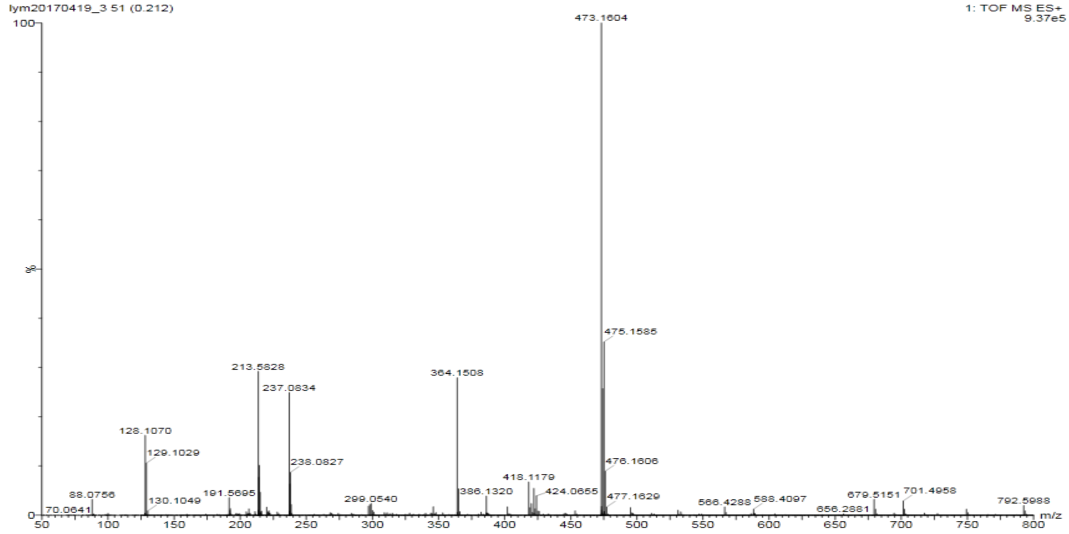

^{1}H-NMR:

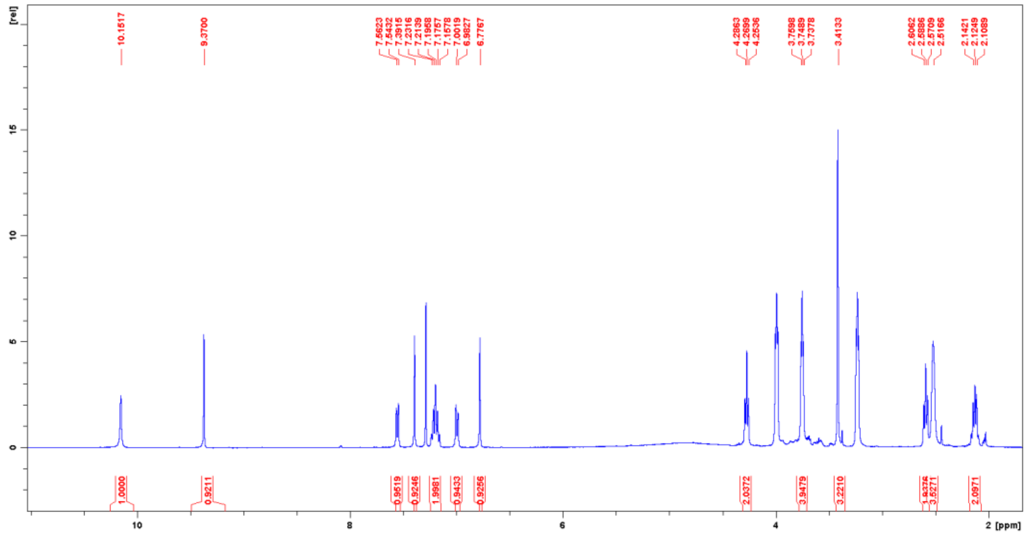

^{13}C-NMR:

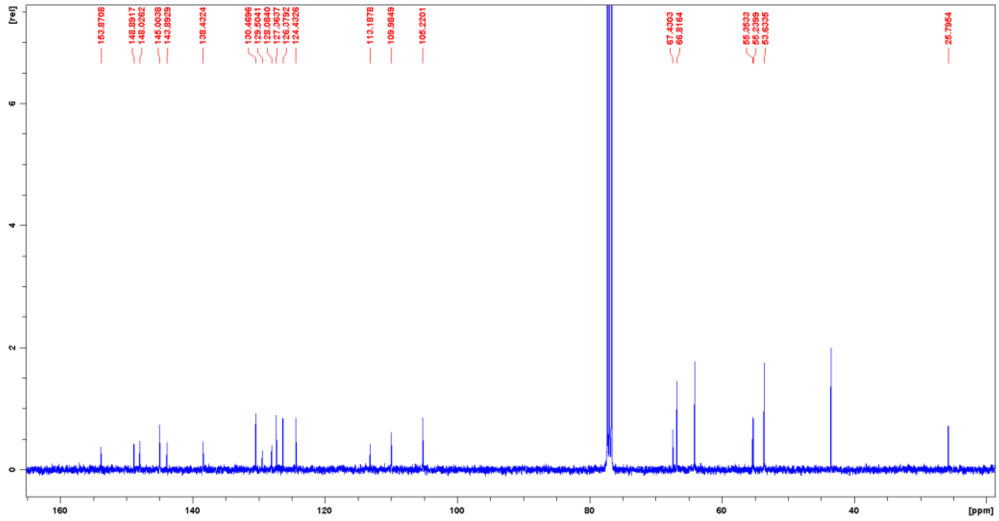

Compound 11c

HR-ESI-MS [M+H]$^+$:

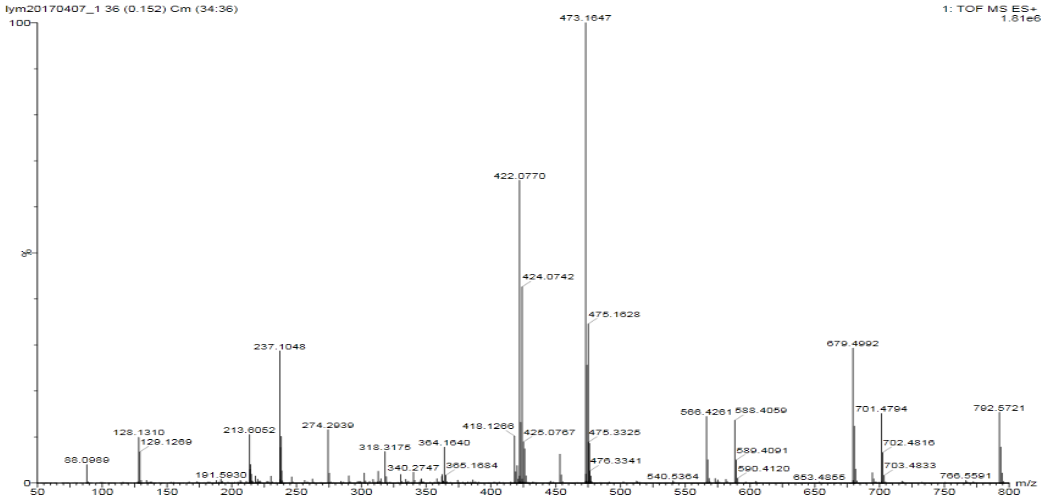

^{1}H-NMR:

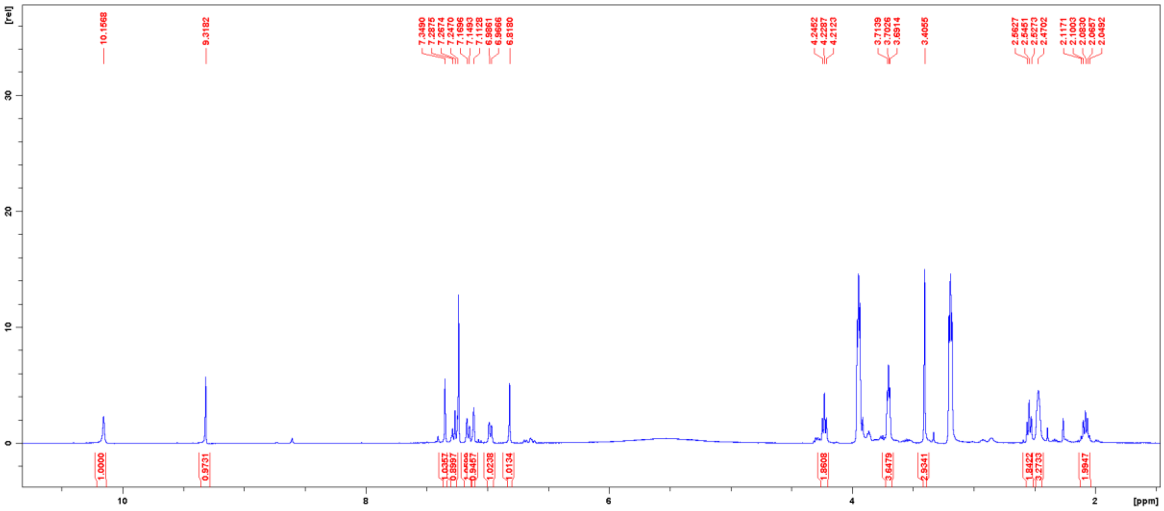

^{13}C-NMR:

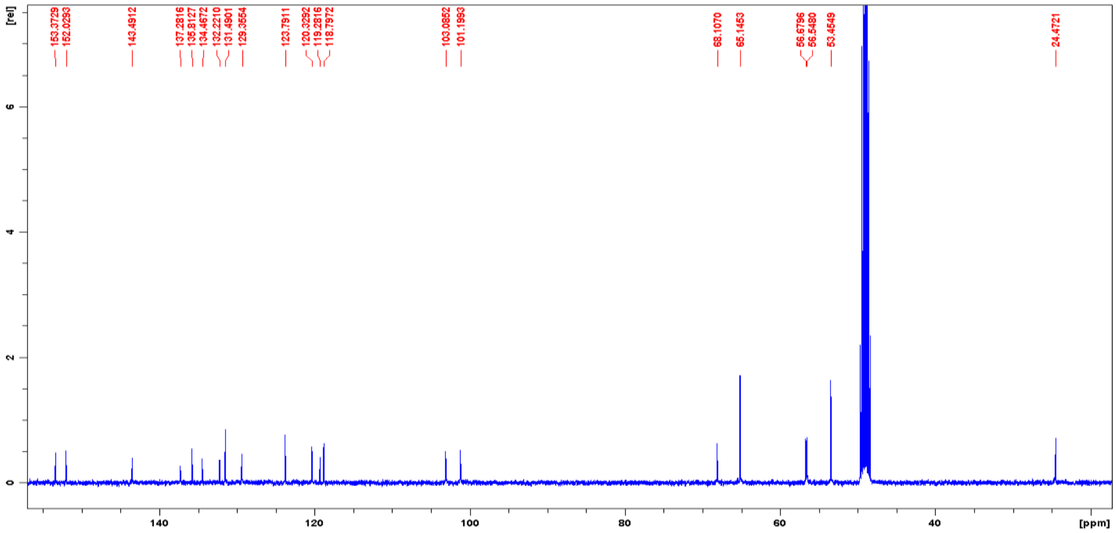

Compound 11d

HR-ESI-MS $[M+H]^+$:

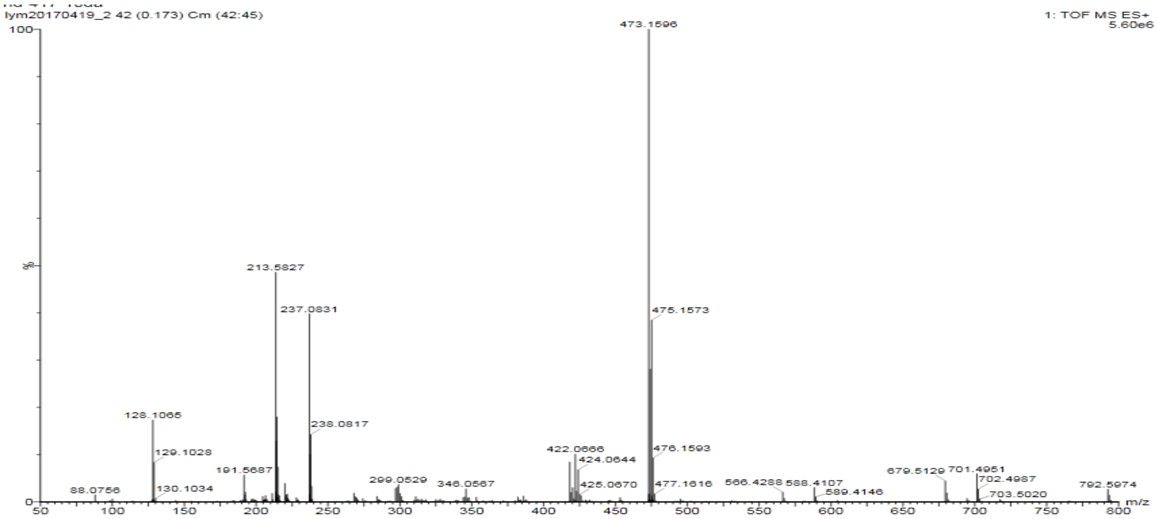

^{1}H-NMR:

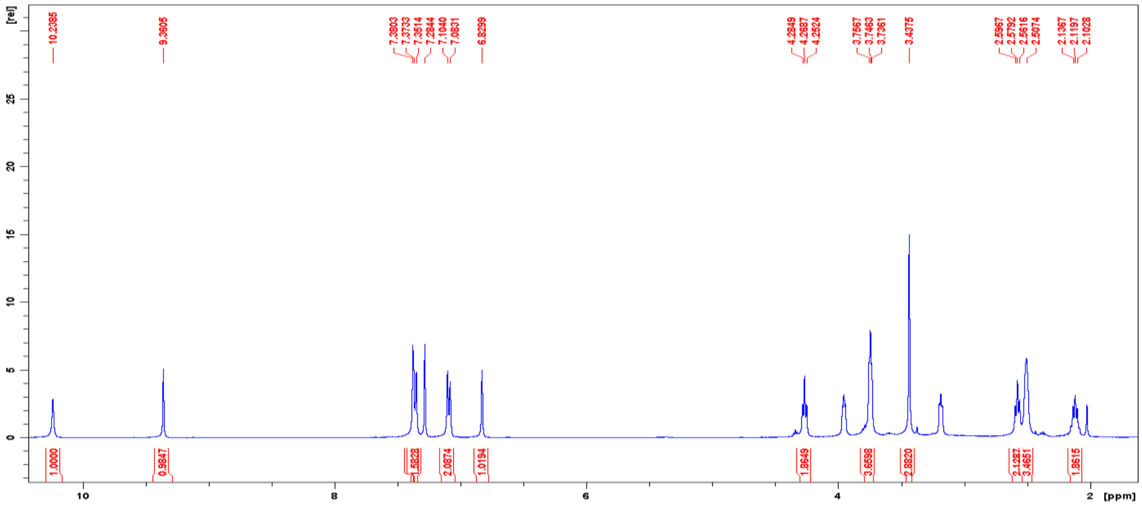

^{13}C-NMR:

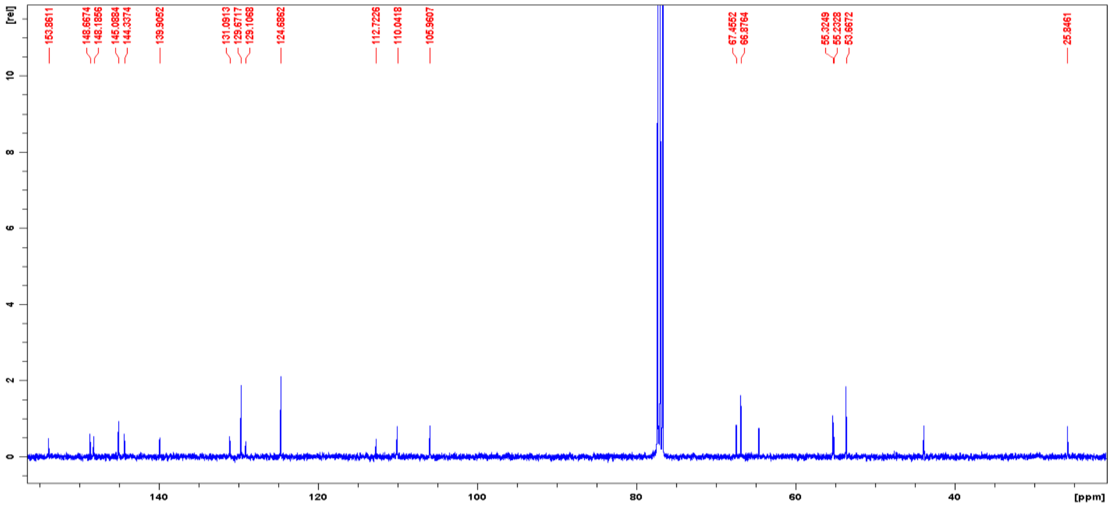

Compound 11e

HR-ESI-MS [M+H]$^{+}$:

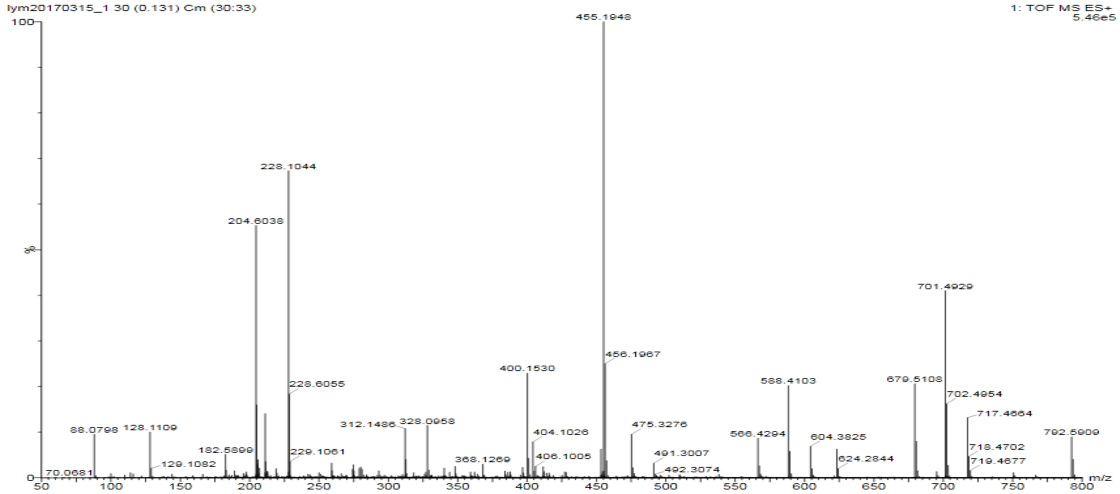

^{1}H-NMR:

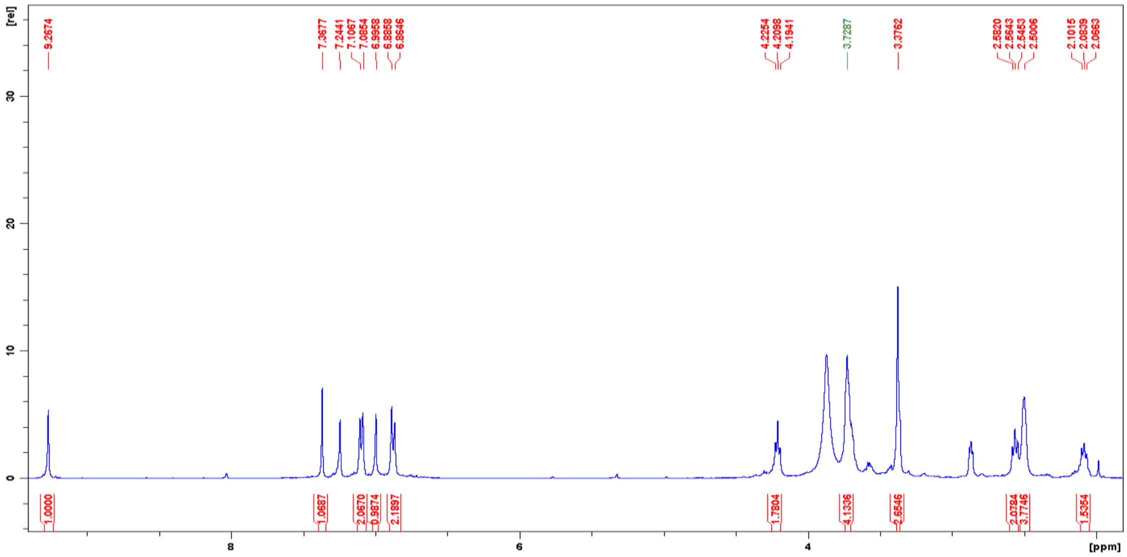

^{13}C-NMR:

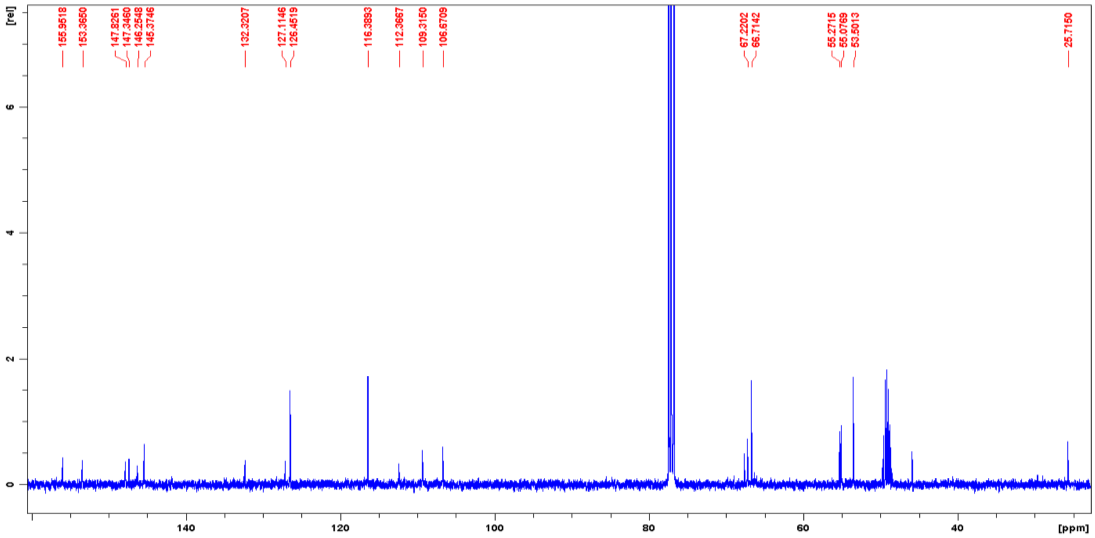

Compound 11f

HR- ESI -MS [M+H]$^{+}$

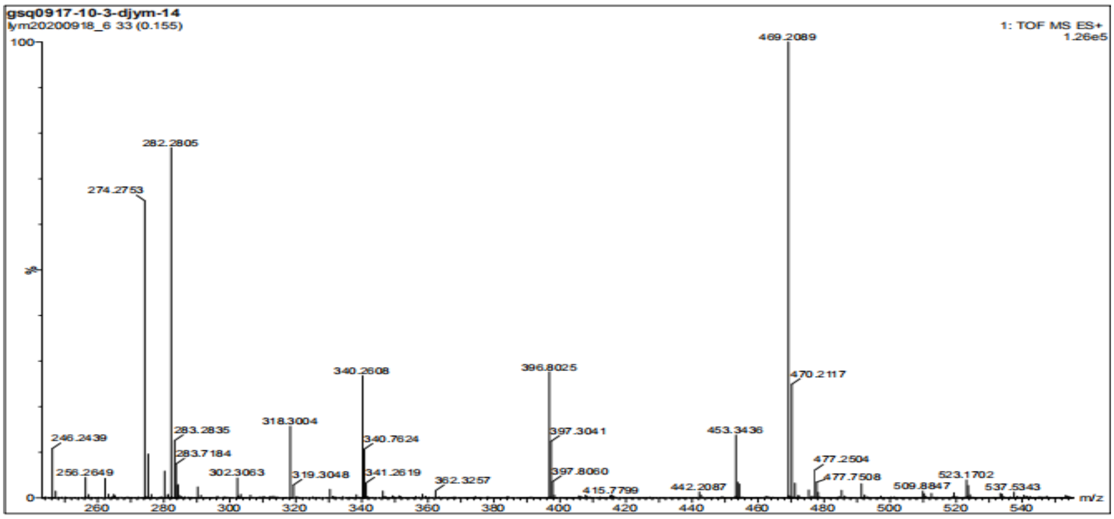

^{1}H-NMR

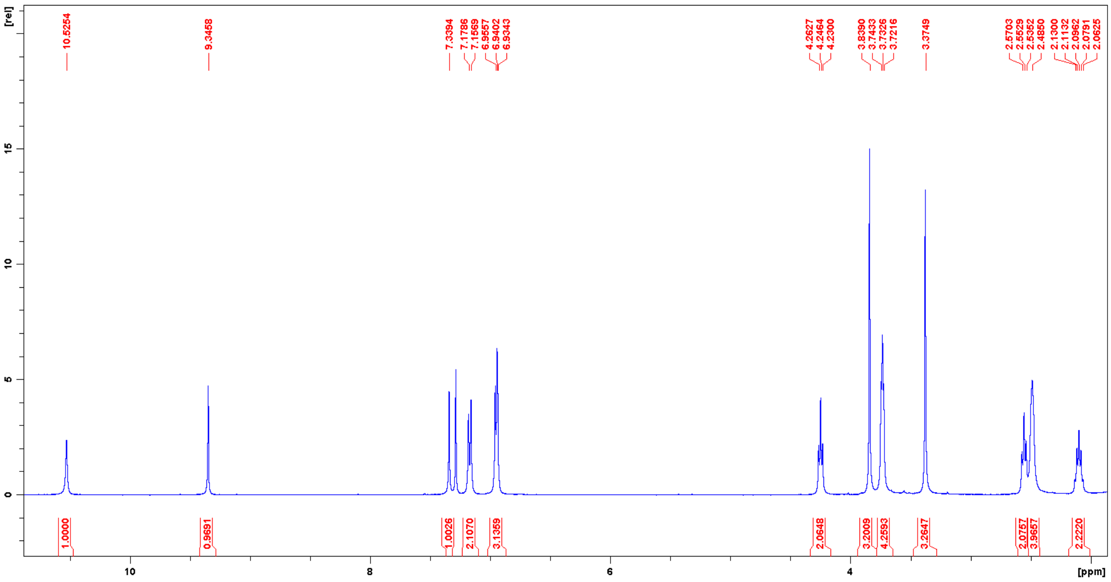

^{13}C-NMR

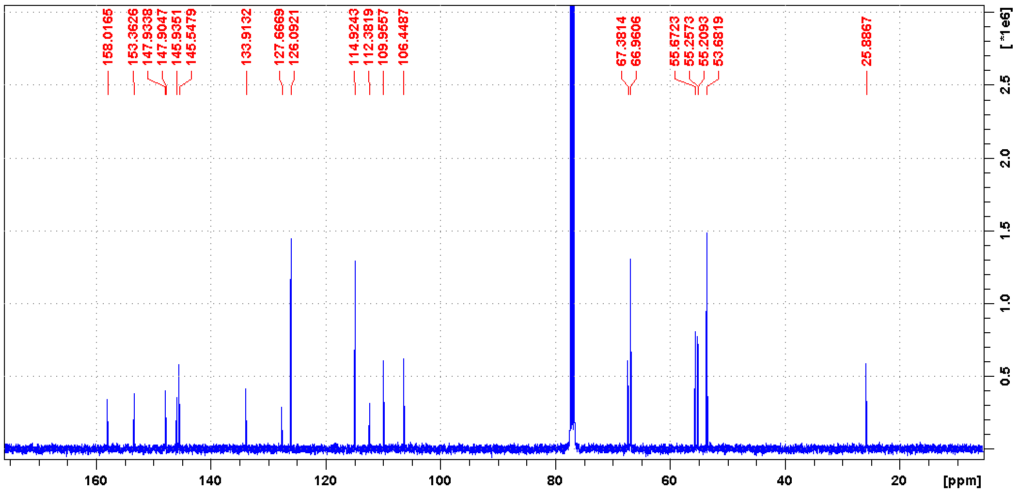

NOESY

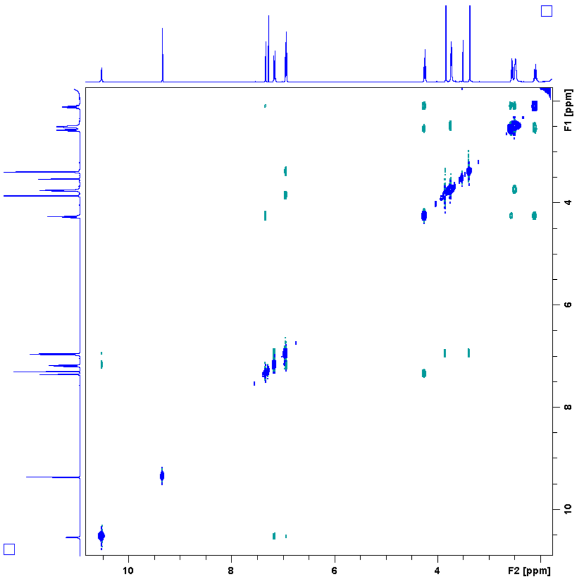

Compound 11g

HR- ESI -MS $[M+H]^+$

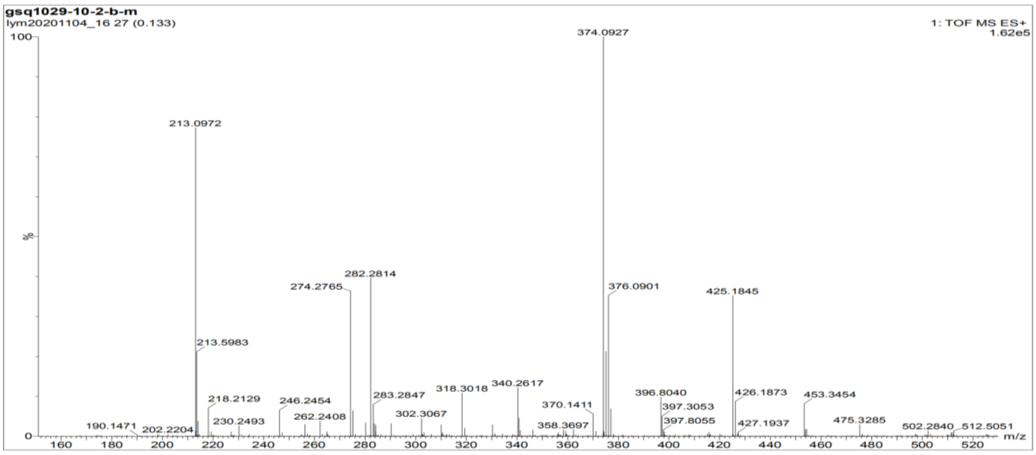

^{1}H-NMR

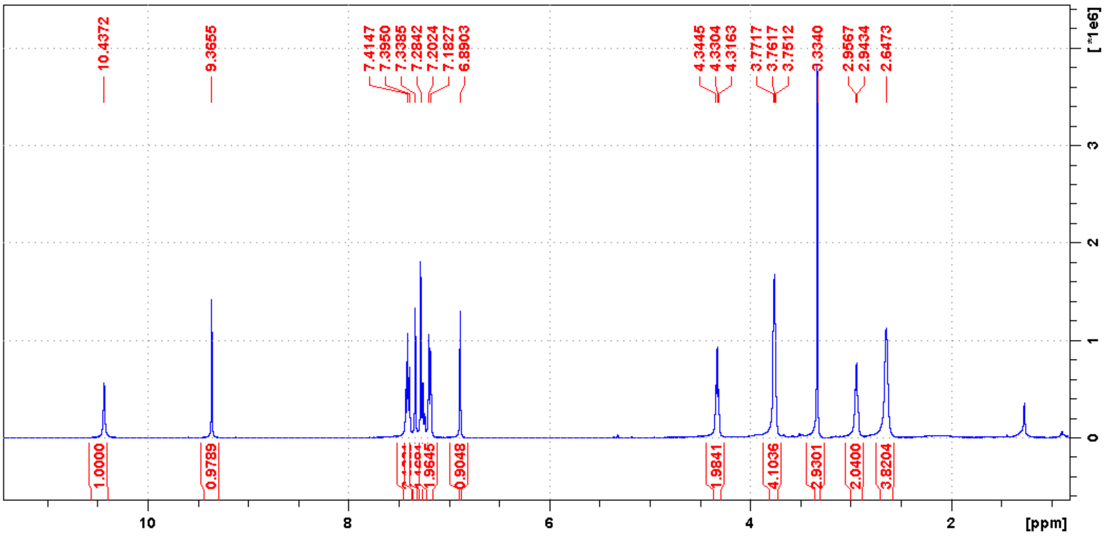

^{13}C-NMR

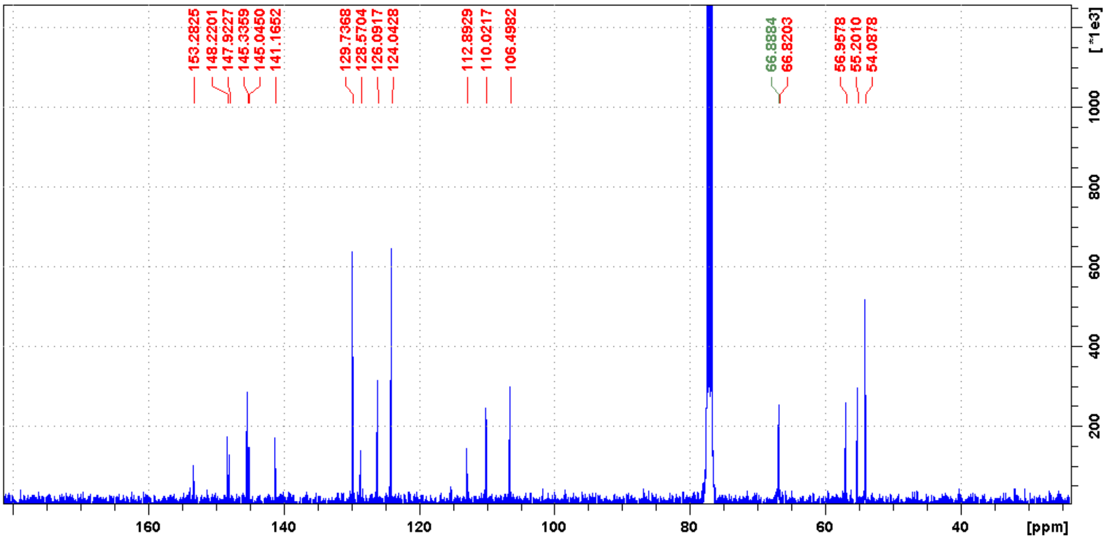

Compound 11h

HR- ESI -MS [M+H]$^+$

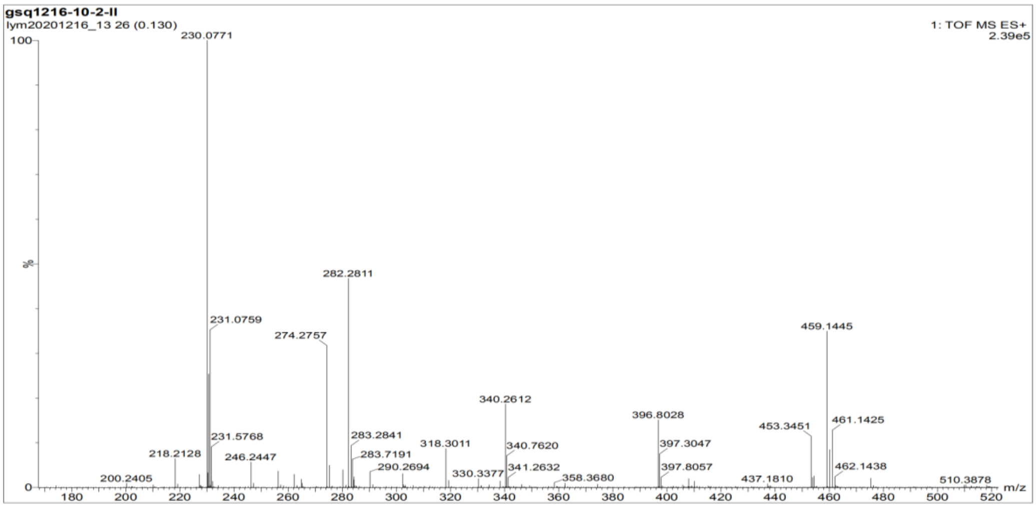

^{1}H-NMR

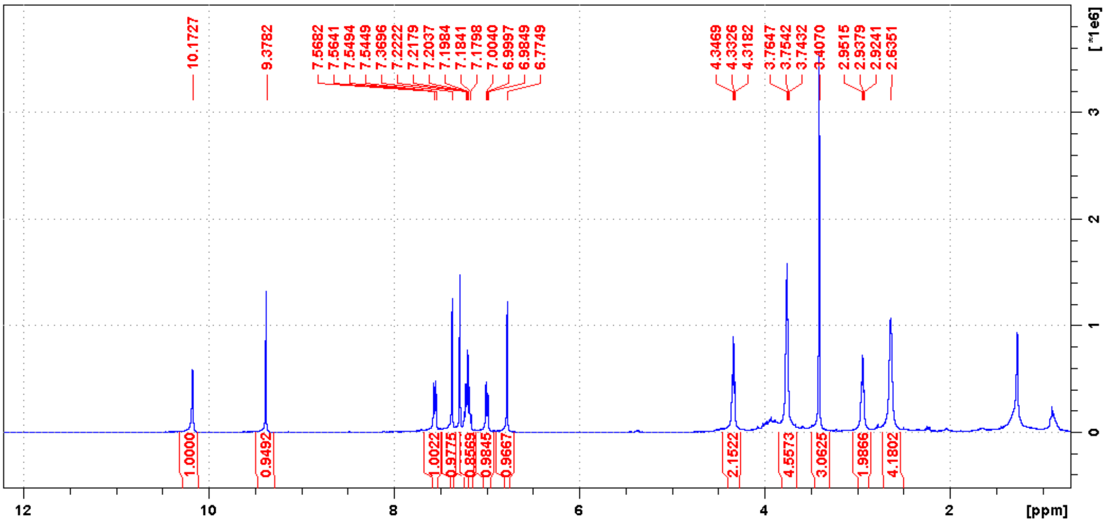

^{13}C-NMR

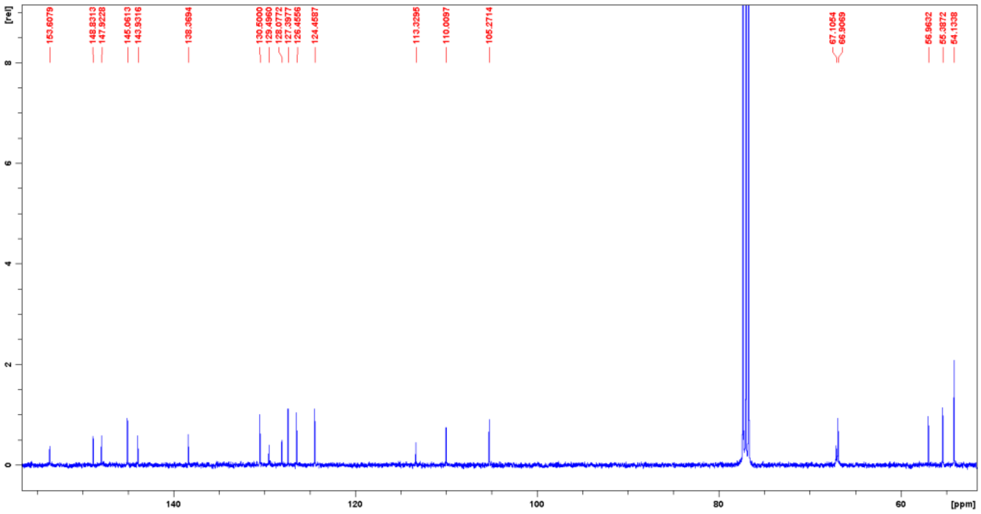

Compound 11i

HR- ESI -MS [M+H]$^+$

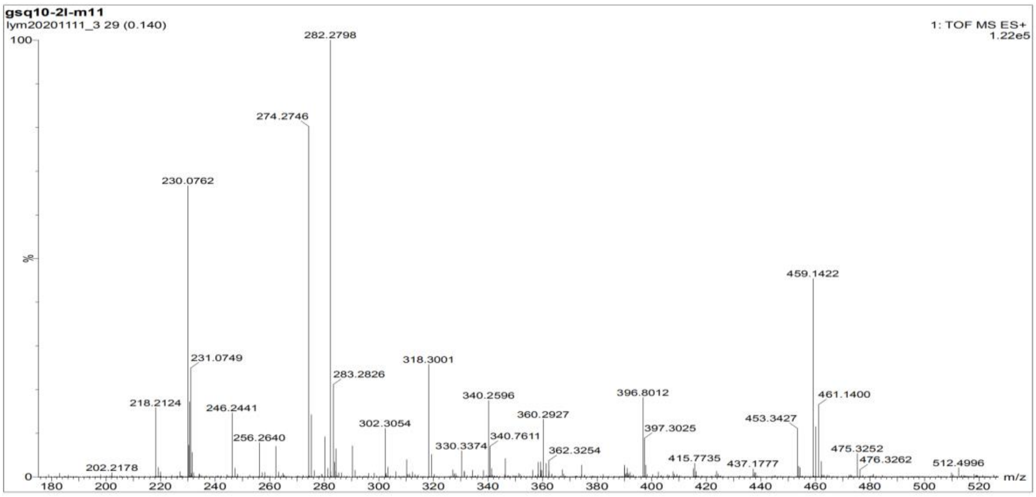

^{1}H-NMR

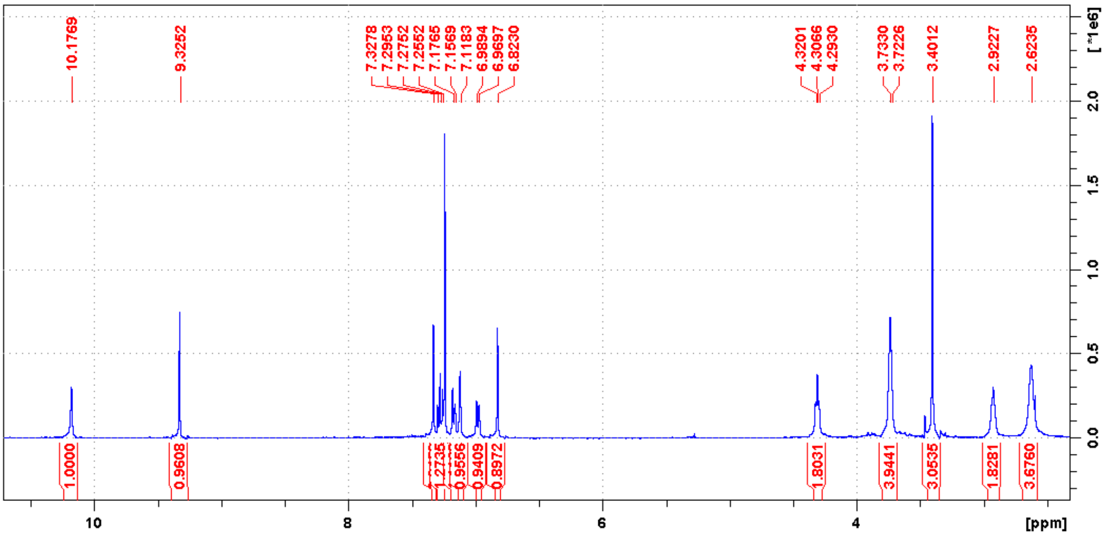

^{13}C-NMR

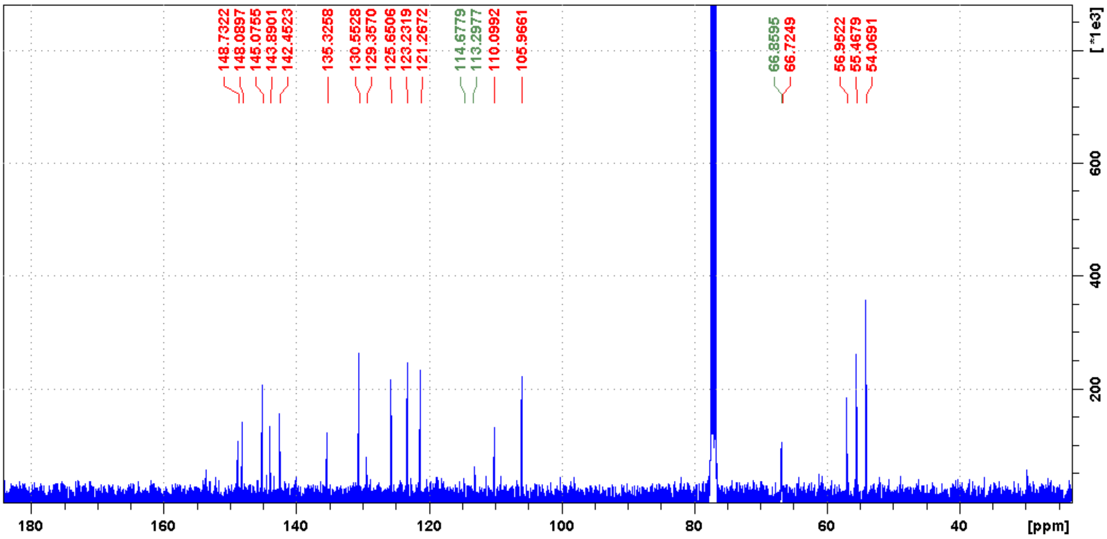

Compound 11j

HR- ESI -MS $[M+H]^+$

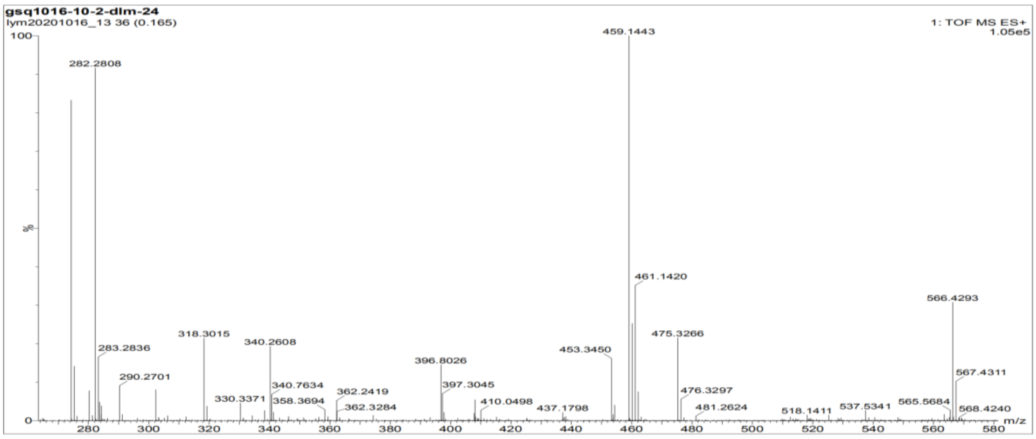

^{1}H-NMR

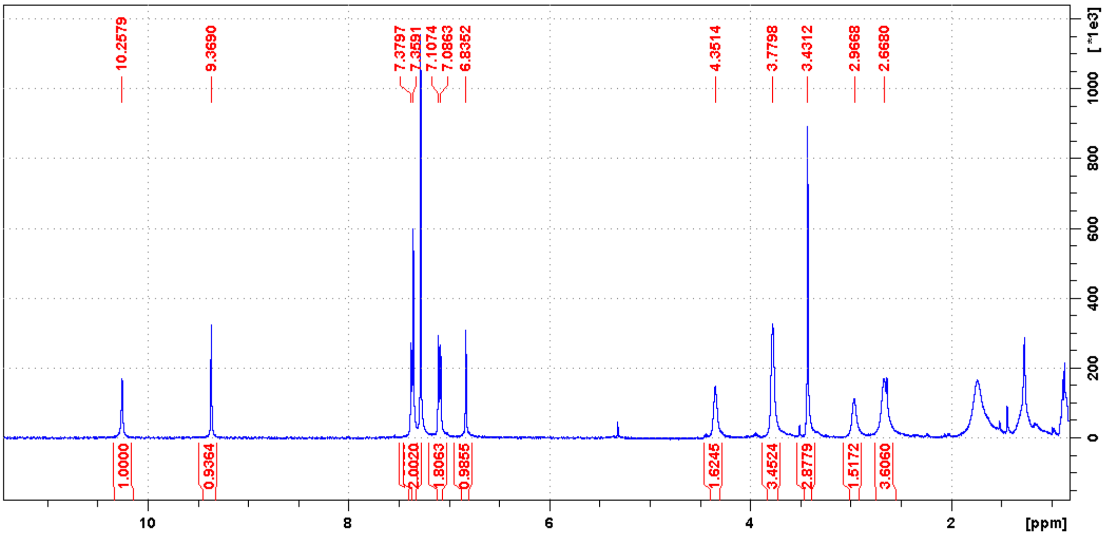

^{13}C-NMR

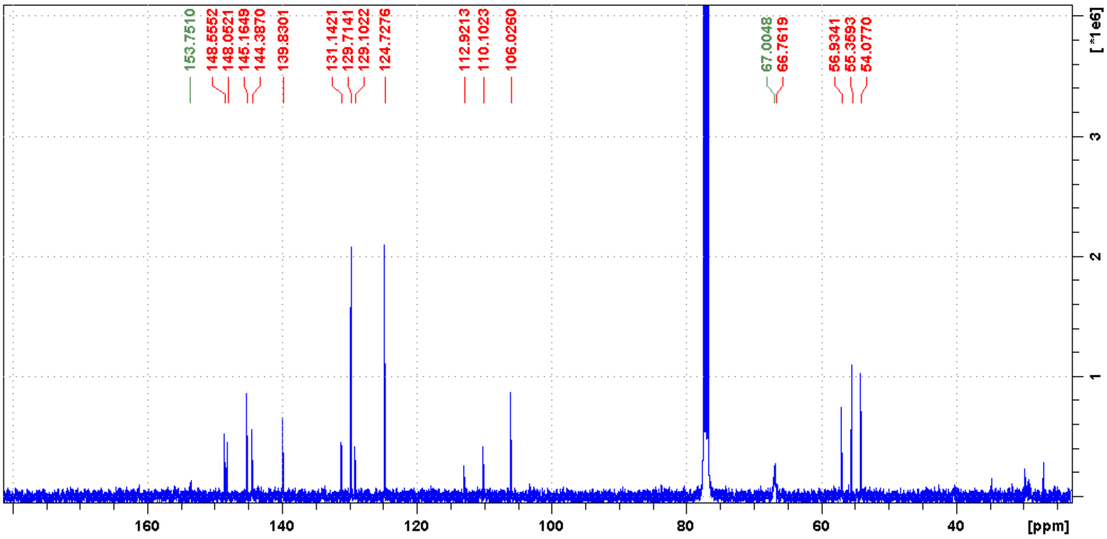

Compound 11k

HR- ESI -MS $[M+H]^+$

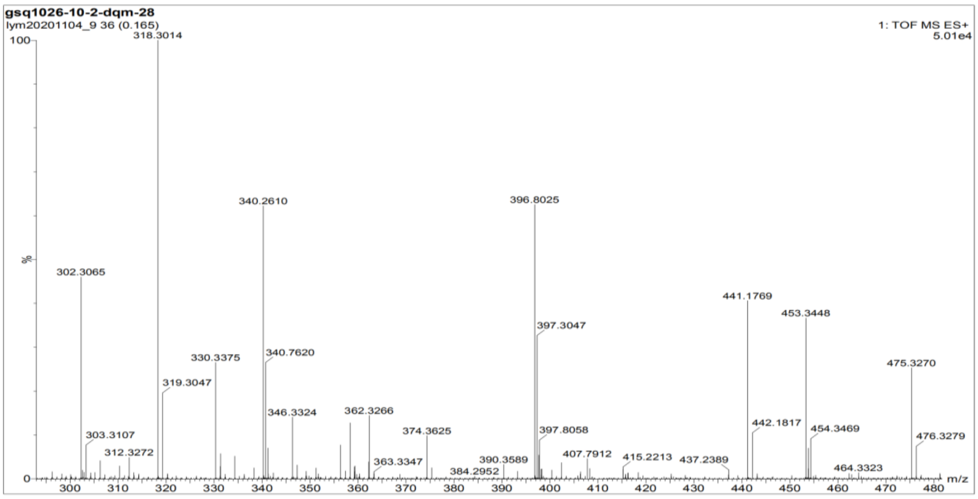

^{1}H-NMR

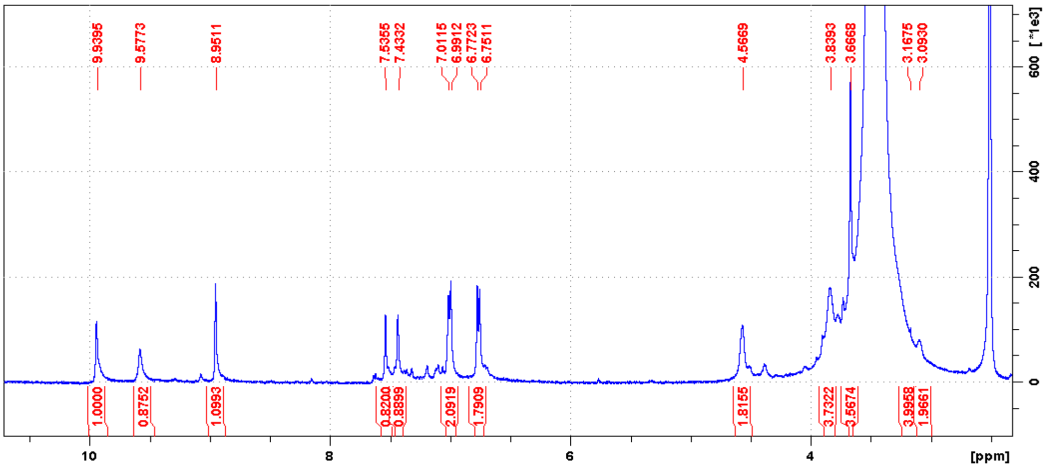

^{13}C-NMR

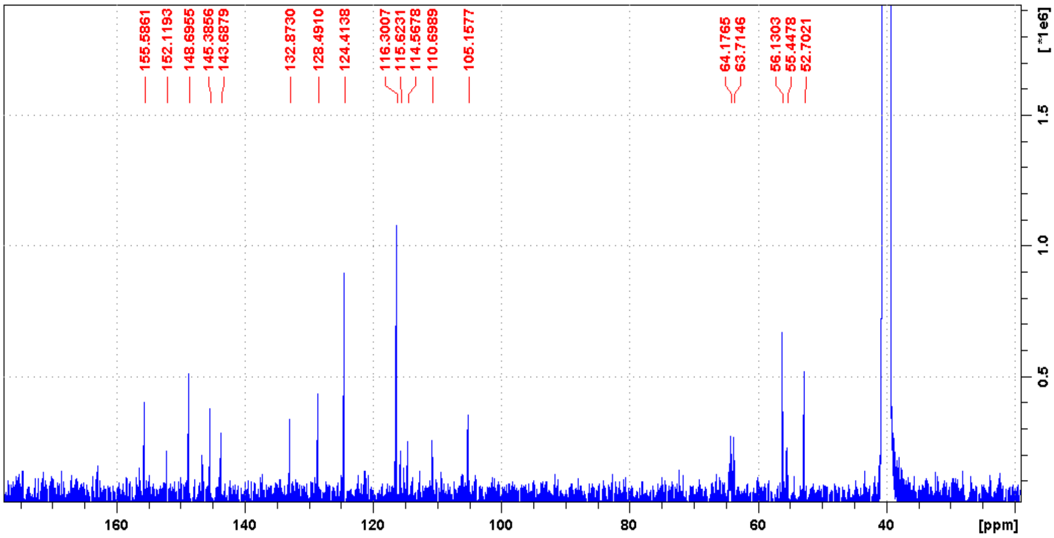

Compound 11l

HR- ESI -MS [M+H]$^+$

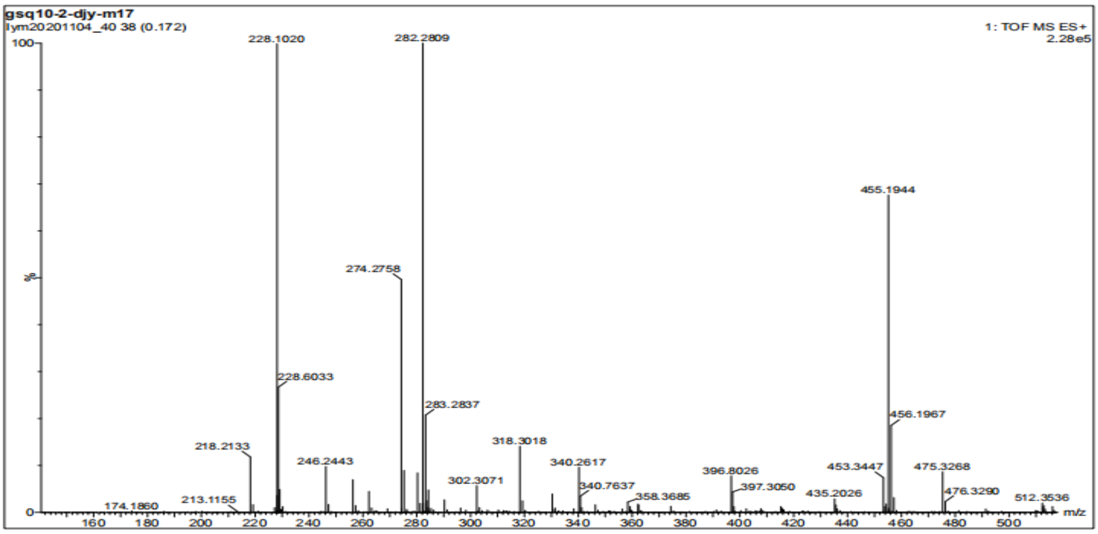

^{1}H-NMR

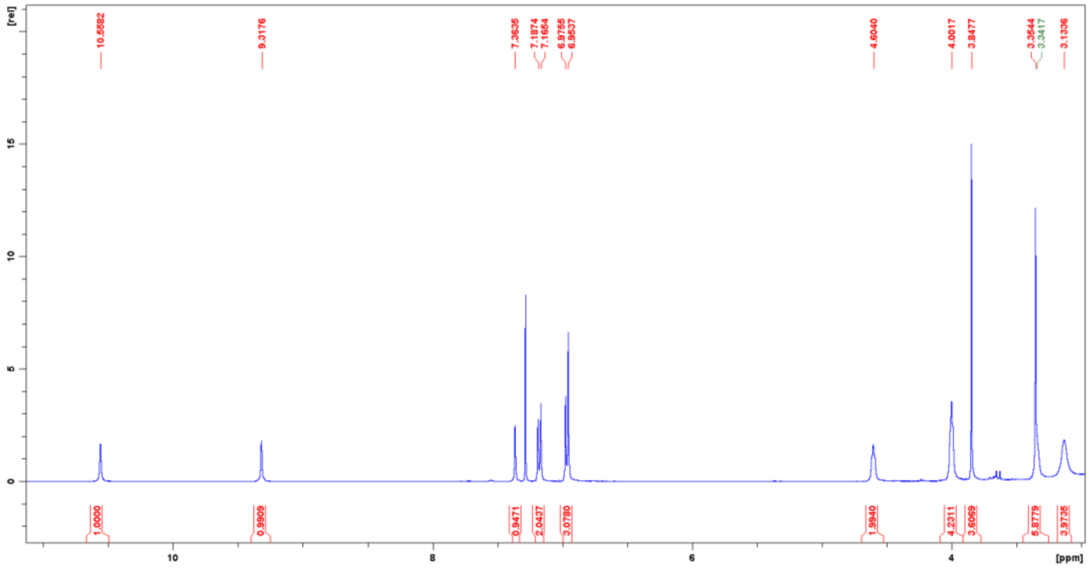

^{13}C-NMR

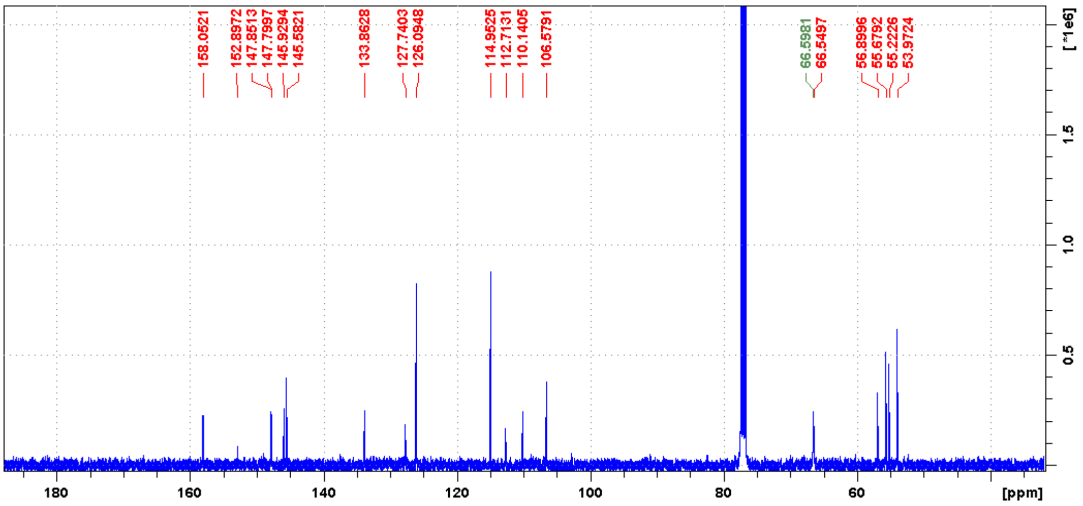

NOESY

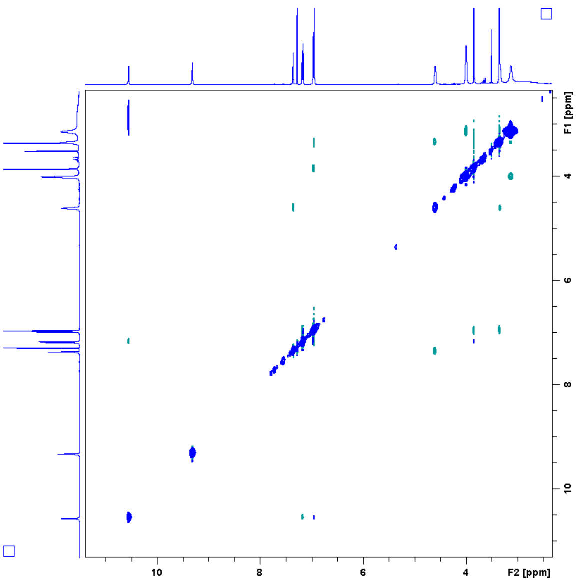

Compound 11m

HR- ESI -MS [M+H]+

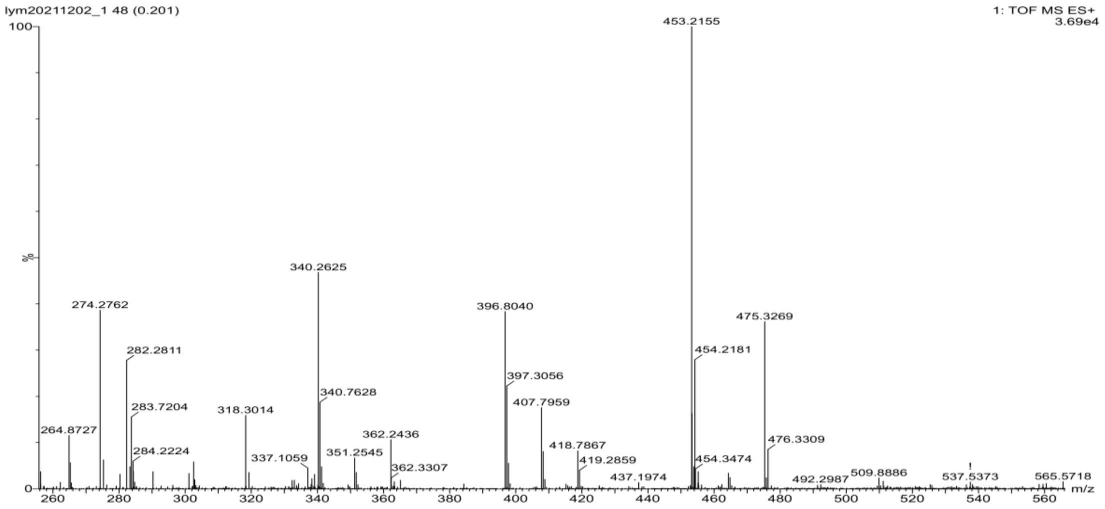

^{1}H-NMR

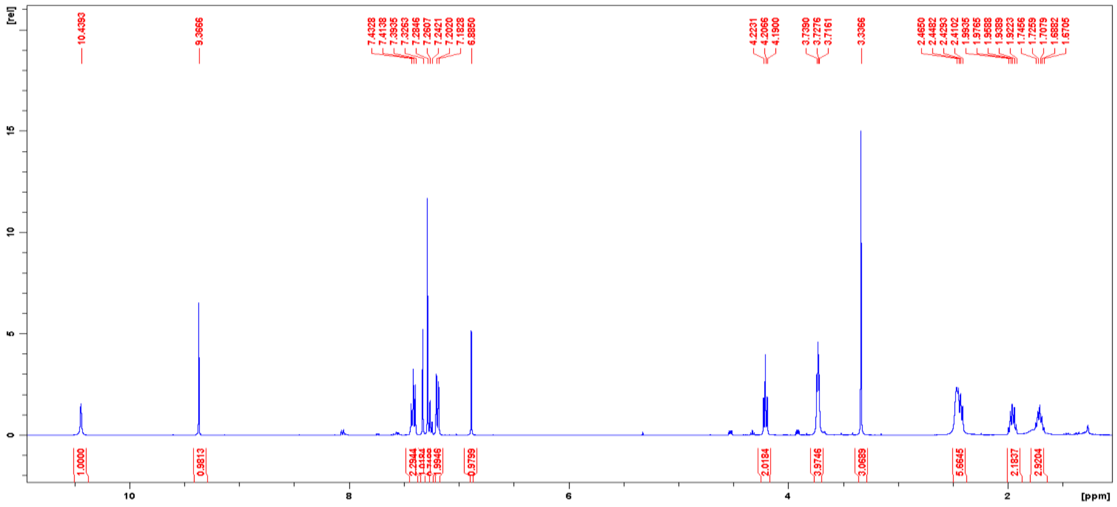

^{13}C-NMR

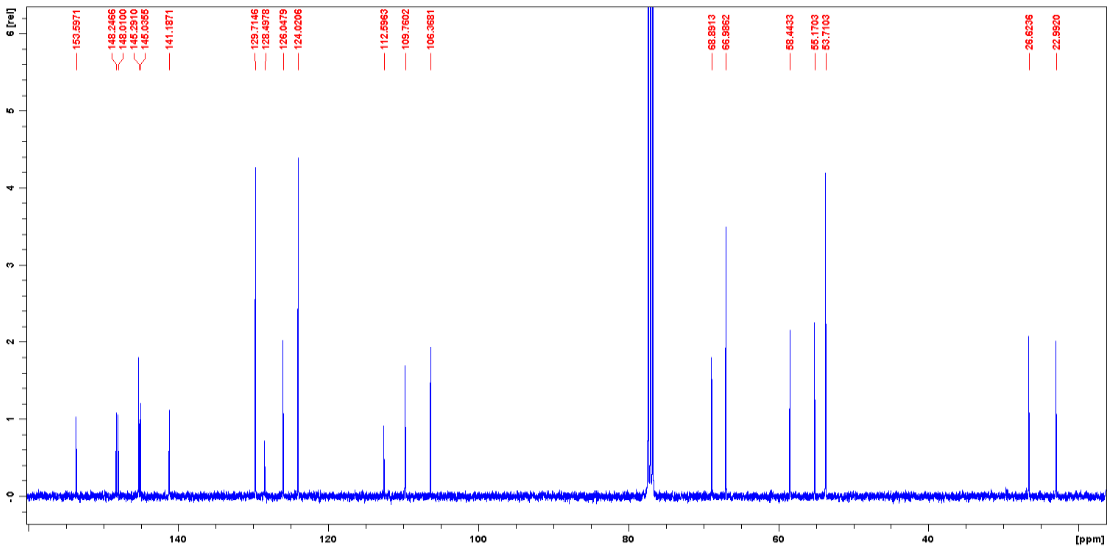

Compound 11n

HR- ESI -MS $[M+H]^+$

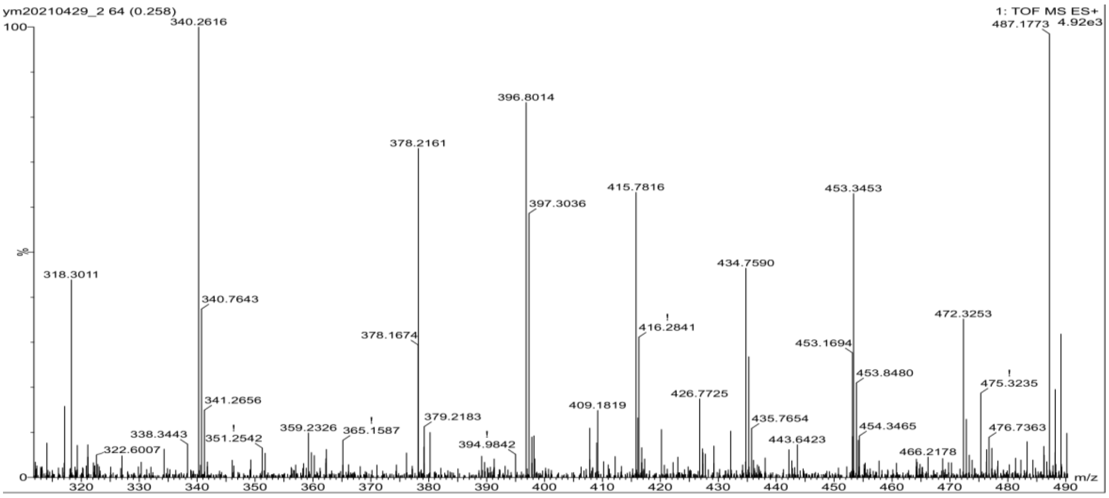

^{1}H-NMR

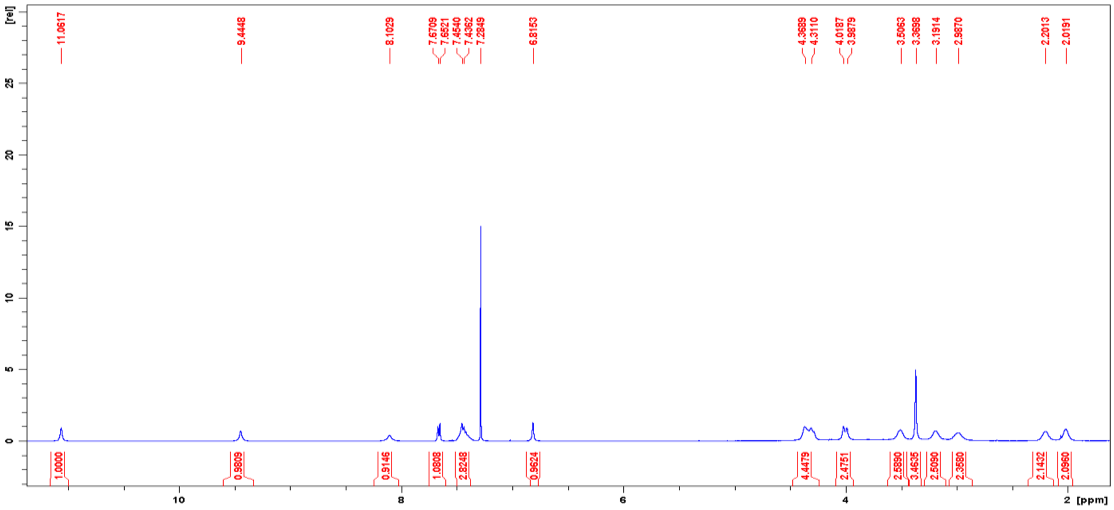

^{13}C-NMR

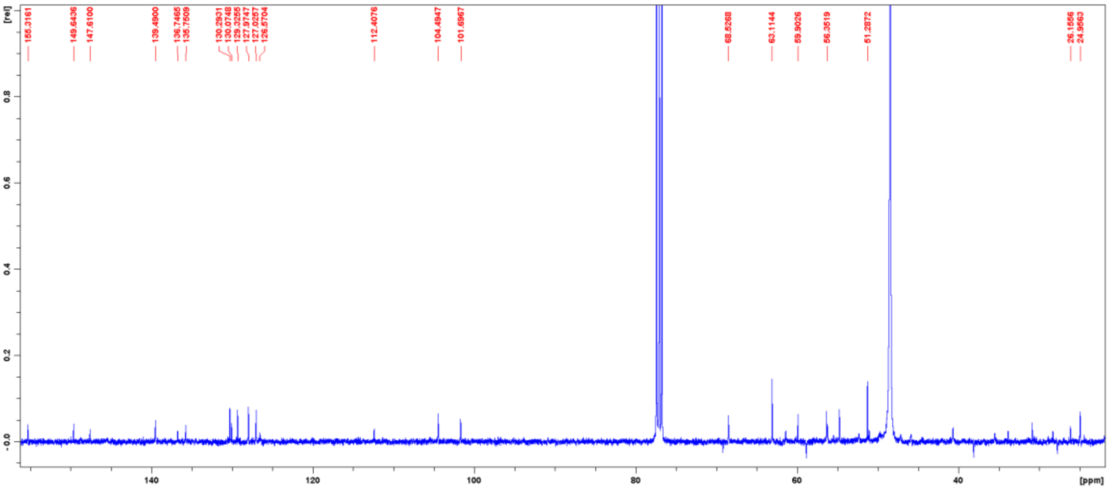

Compound 11o

HR- ESI -MS [M+H]$^+$

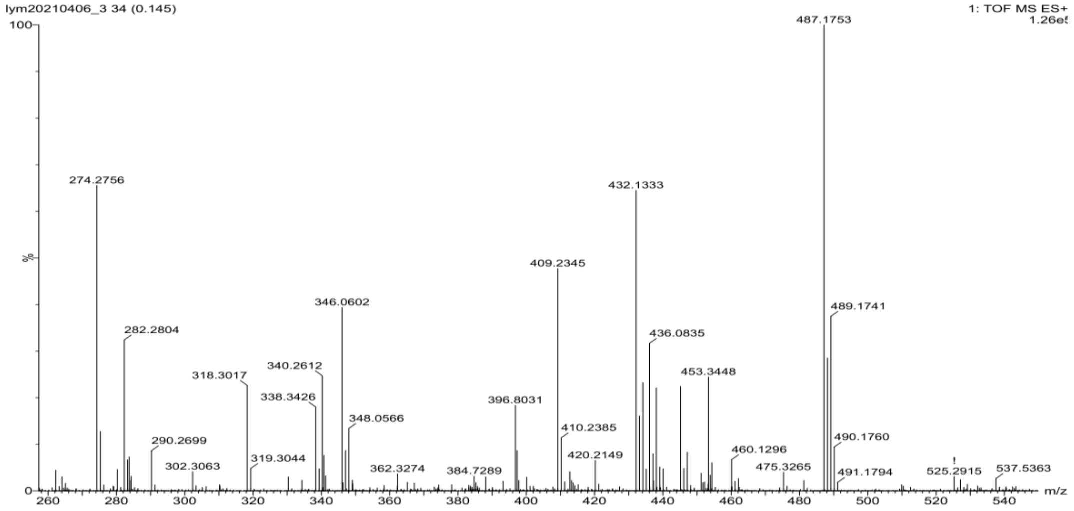

^{1}H-NMR

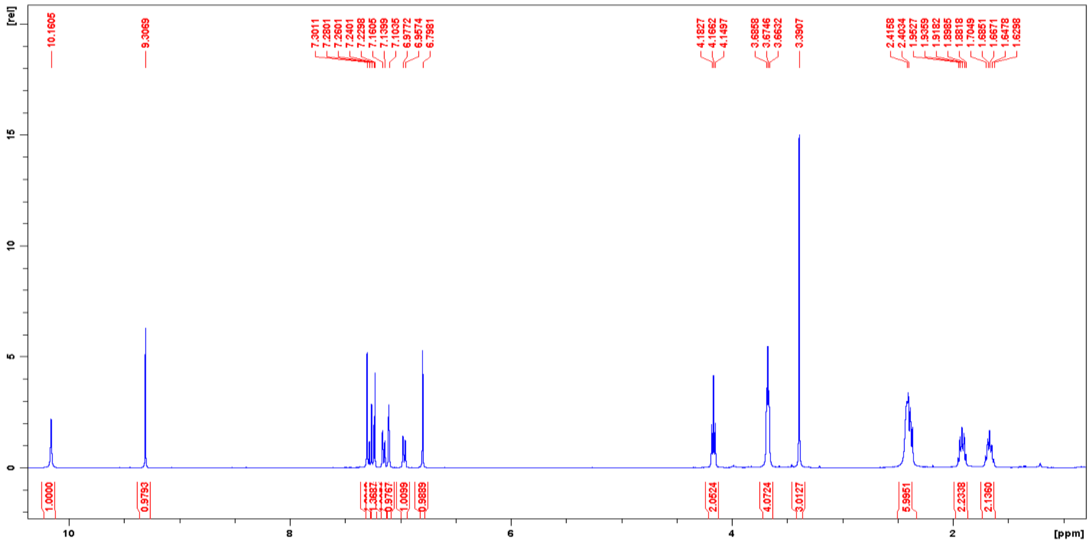

^{13}C-NMR

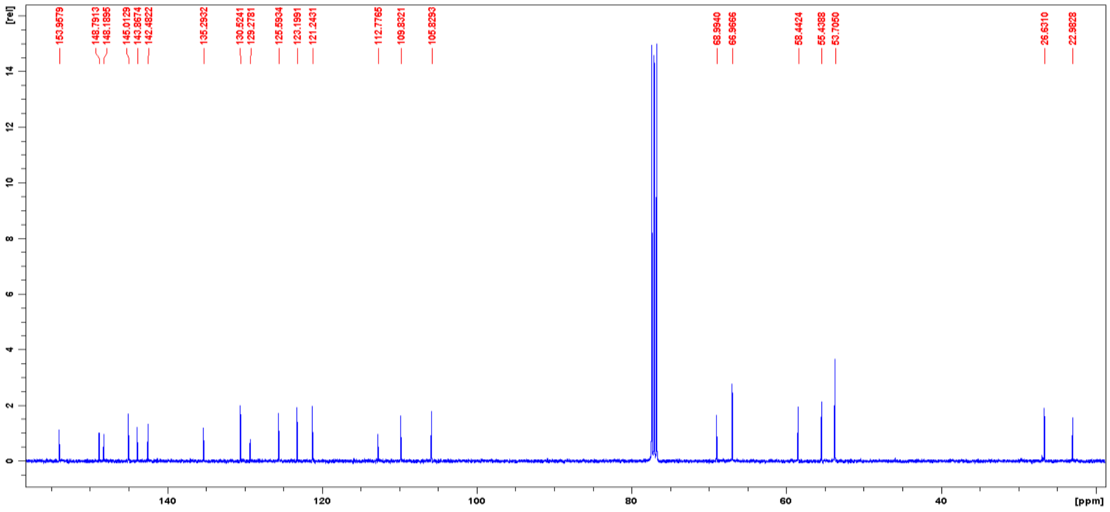

Compound 11p

HR- ESI -MS $[M+H]^+$

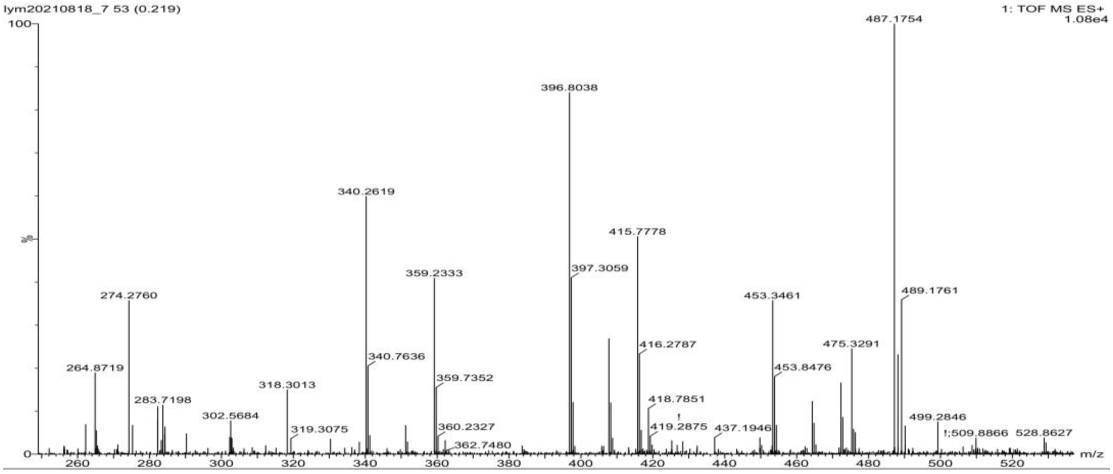

^{1}H-NMR

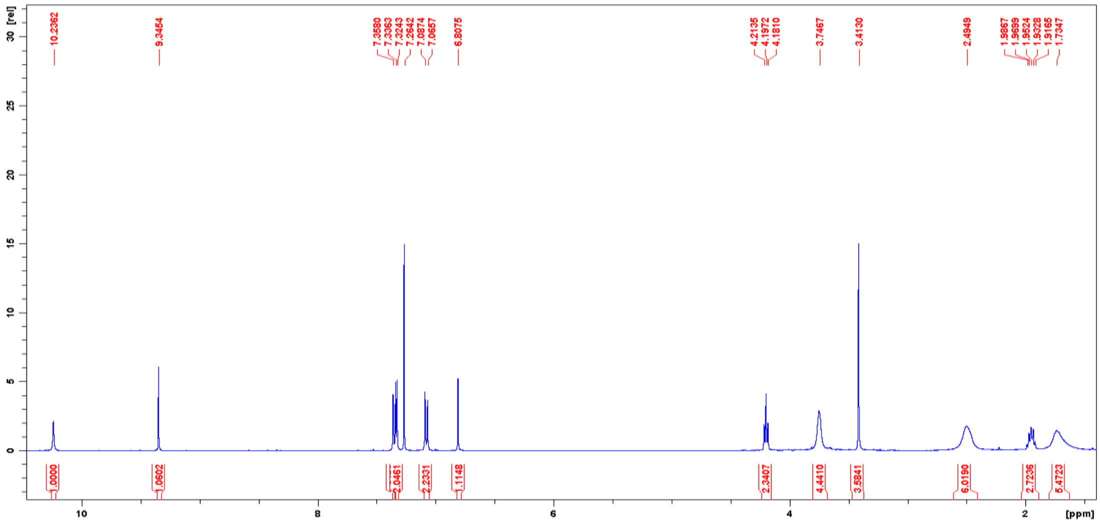

^{13}C-NMR

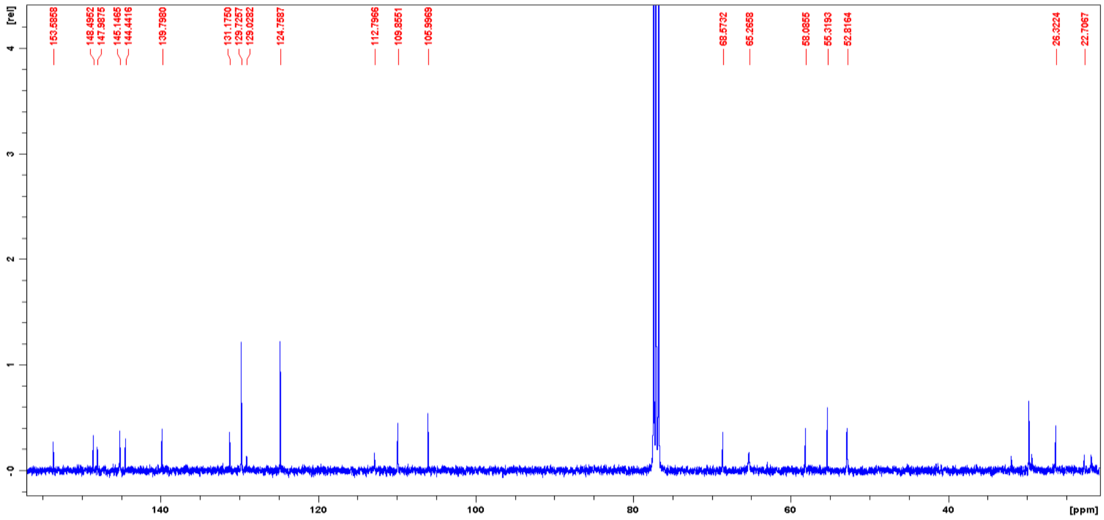

Compound 11q

HR- ESI -MS $[M+H]^+$

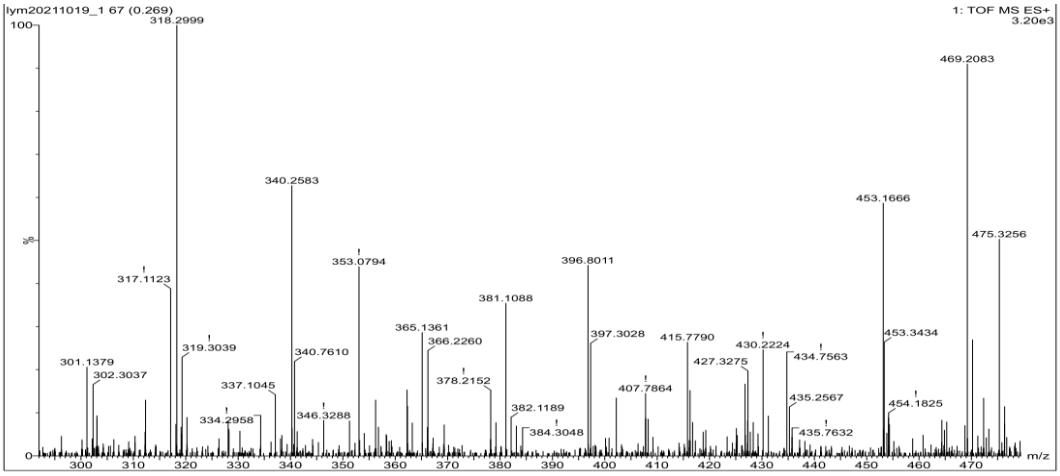

^{1}H-NMR

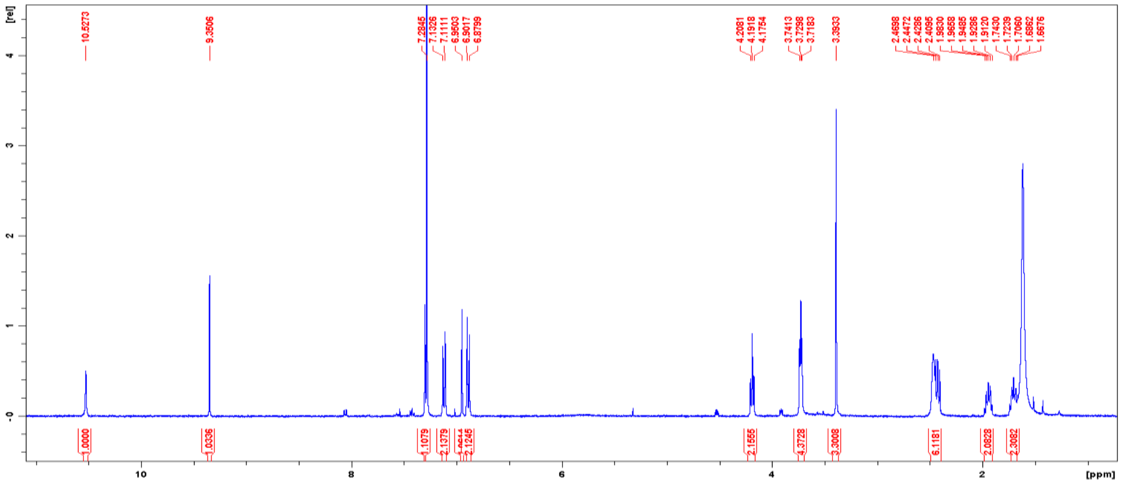

^{13}C-NMR

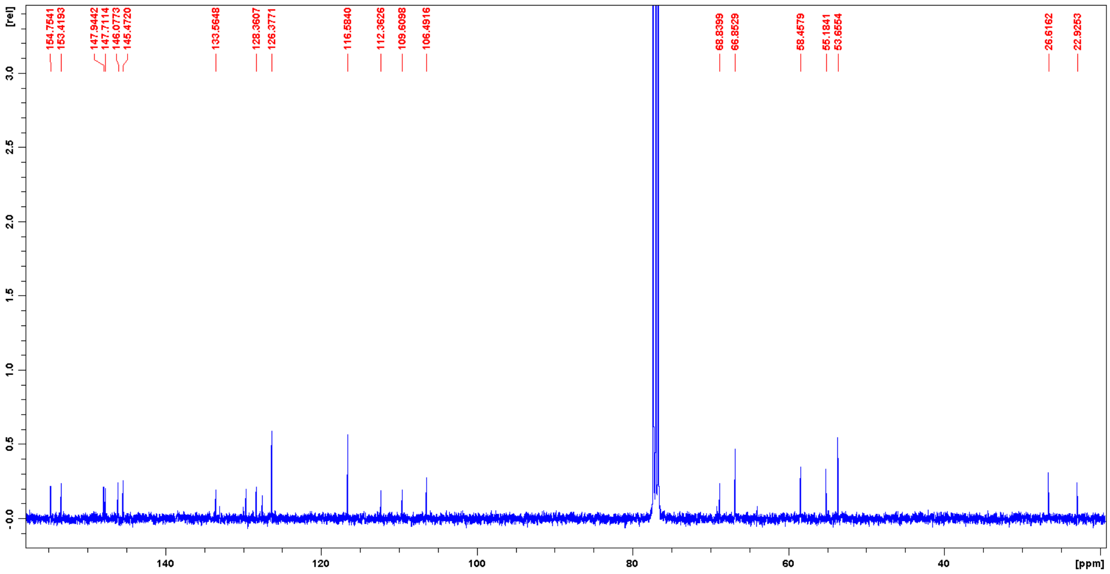

Compound 11r

HR- ESI -MS $[M+H]^+$

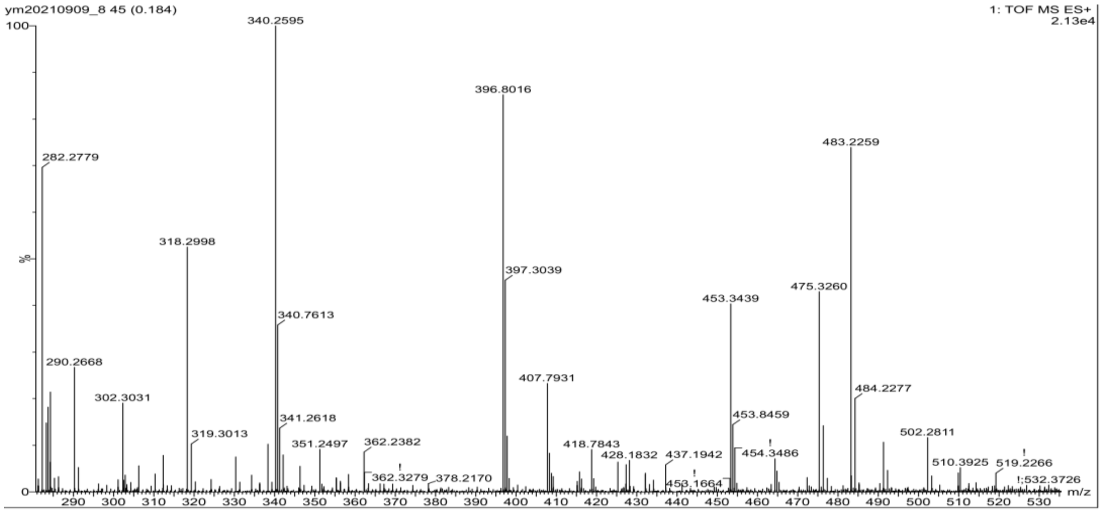

^{1}H-NMR

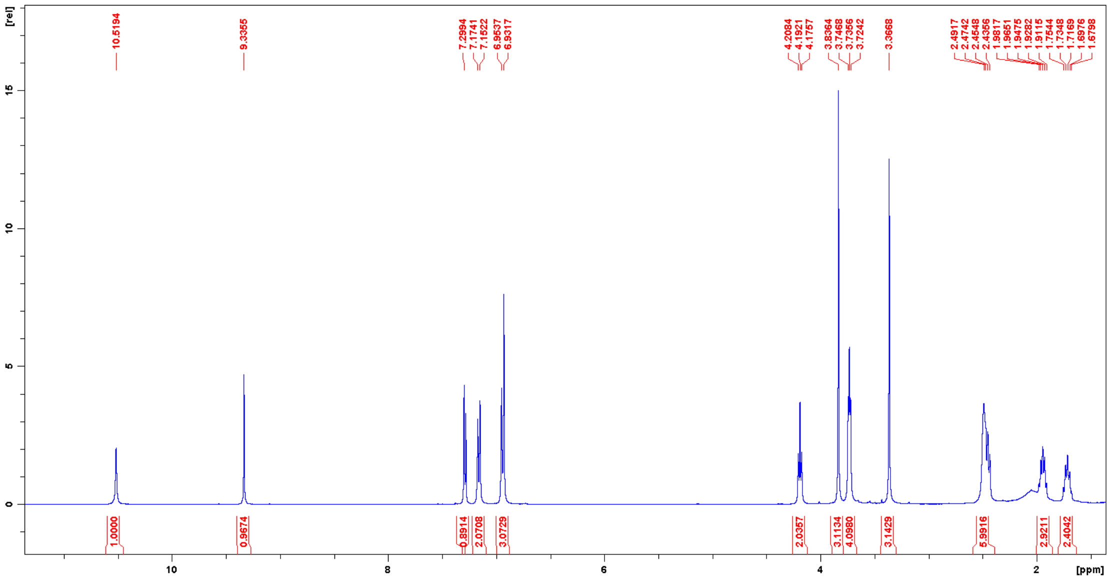

^{13}C-NMR

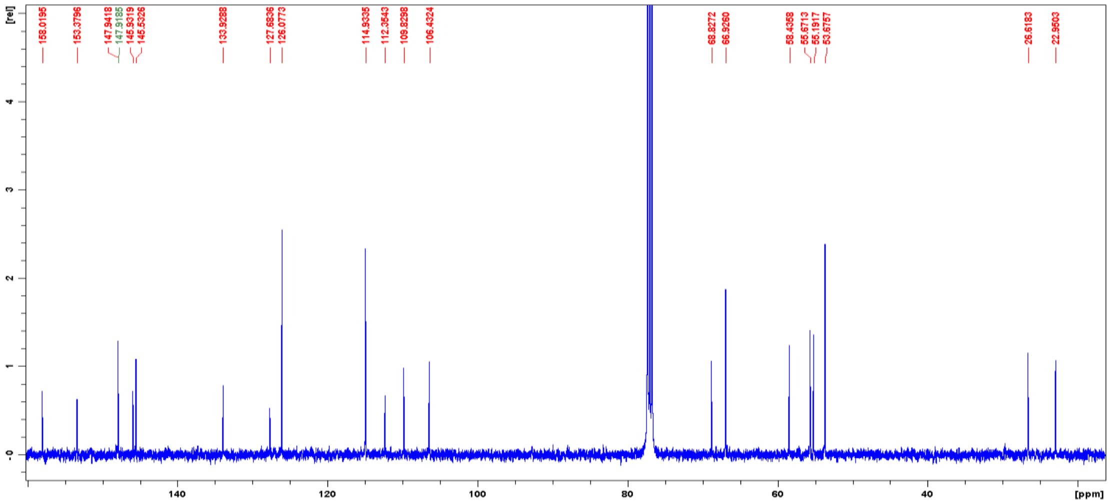

NOESY

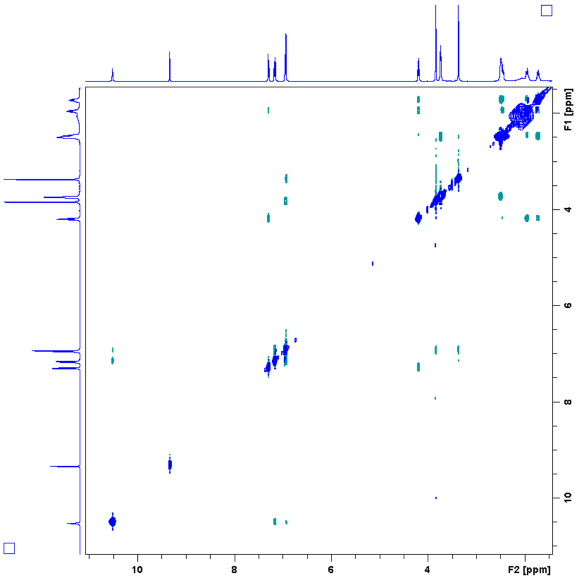

Compound 12a

HR-ESI-MS [M+H]+

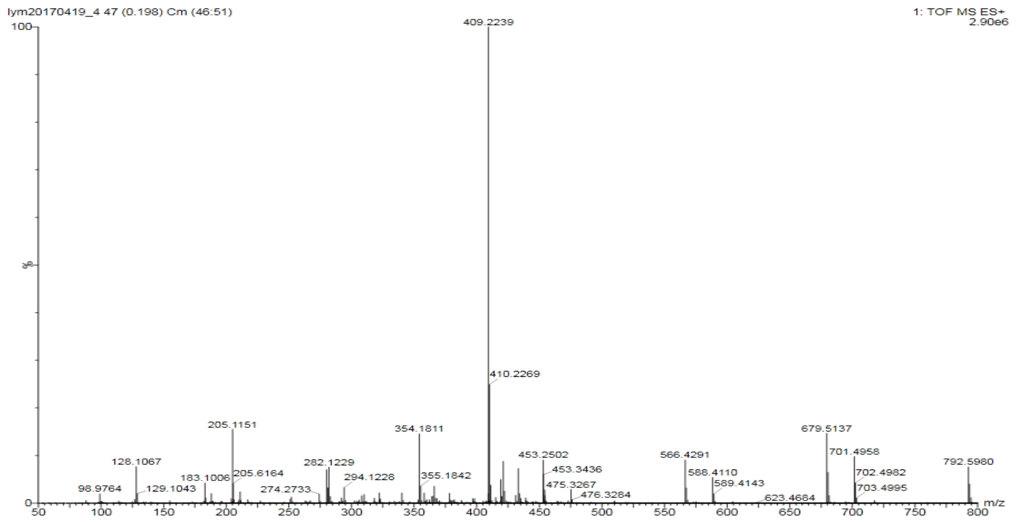

^{1}H-NMR

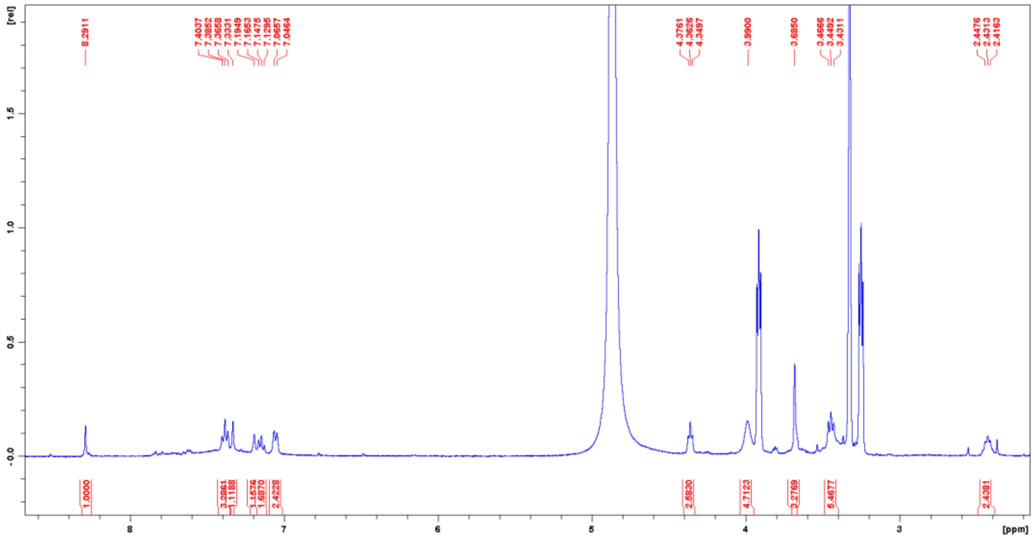

^{13}C-NMR

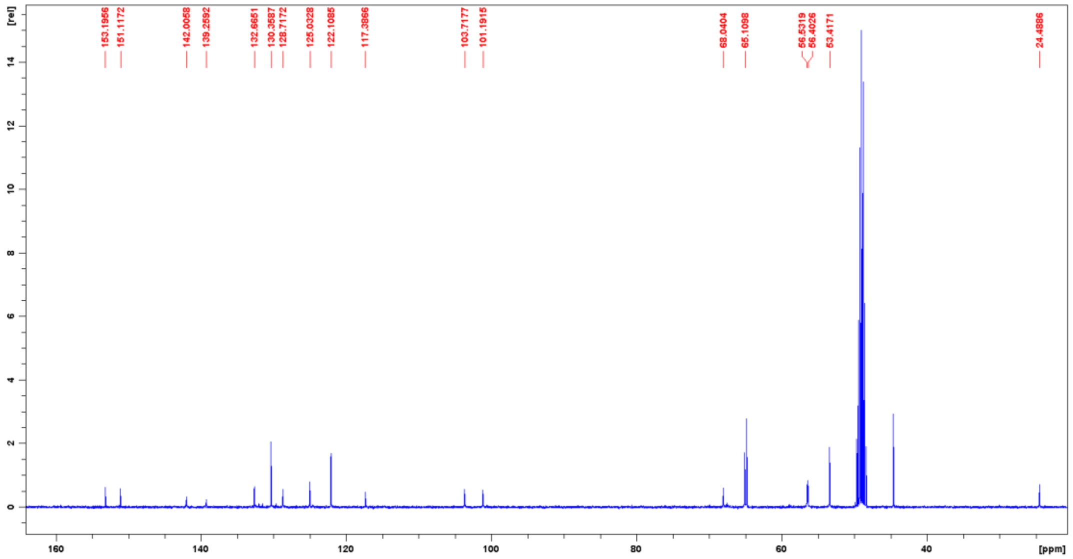

Compound 12b

HR-ESI-MS [M+H]+

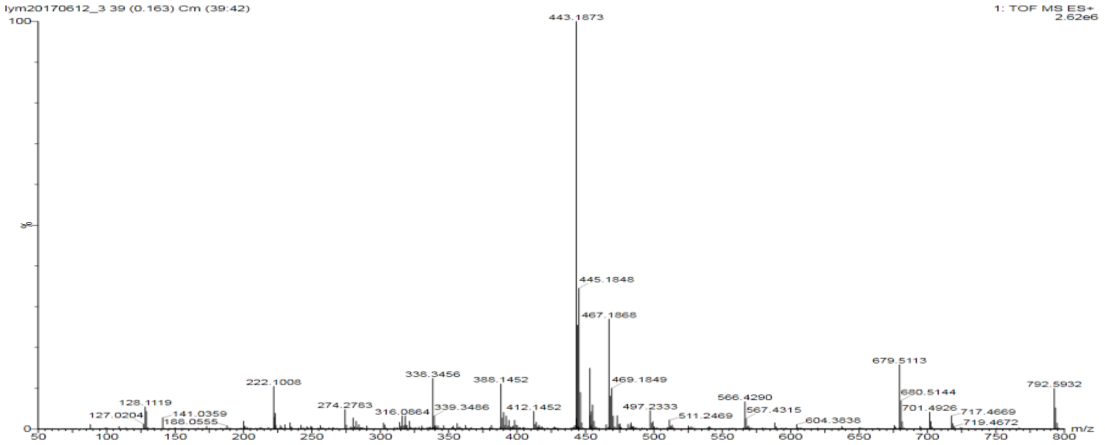

^{1}H-NMR

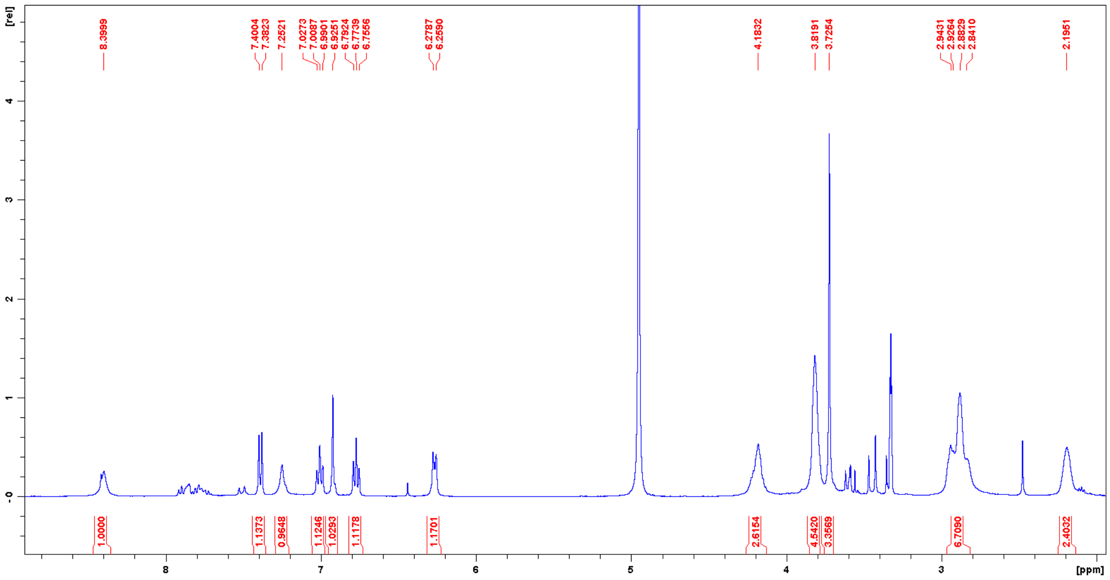

^{13}C-NMR

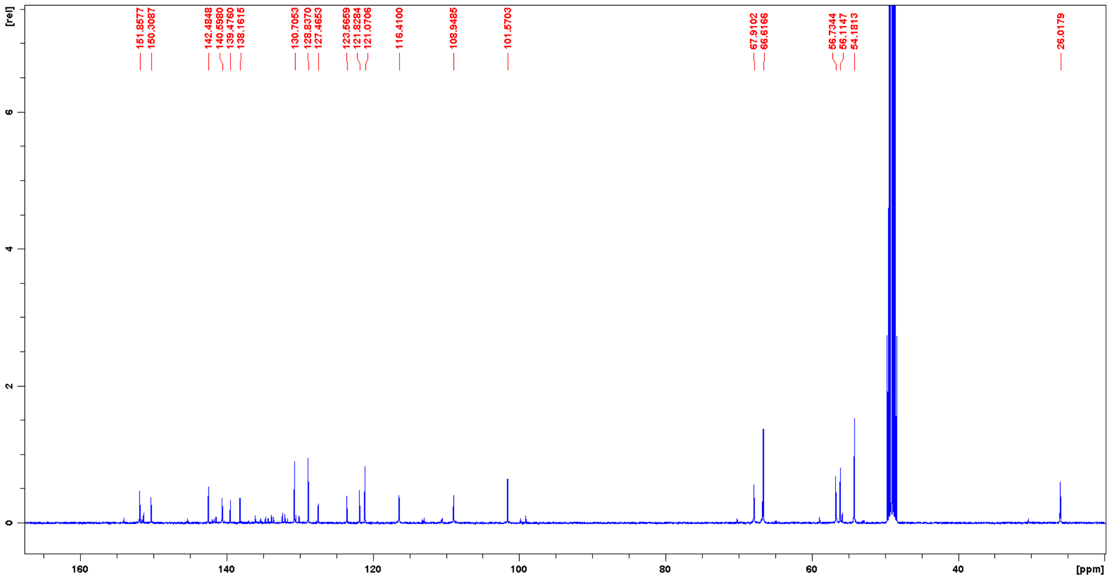

Compound 12c

HR-ESI-MS $[M+H]^+$

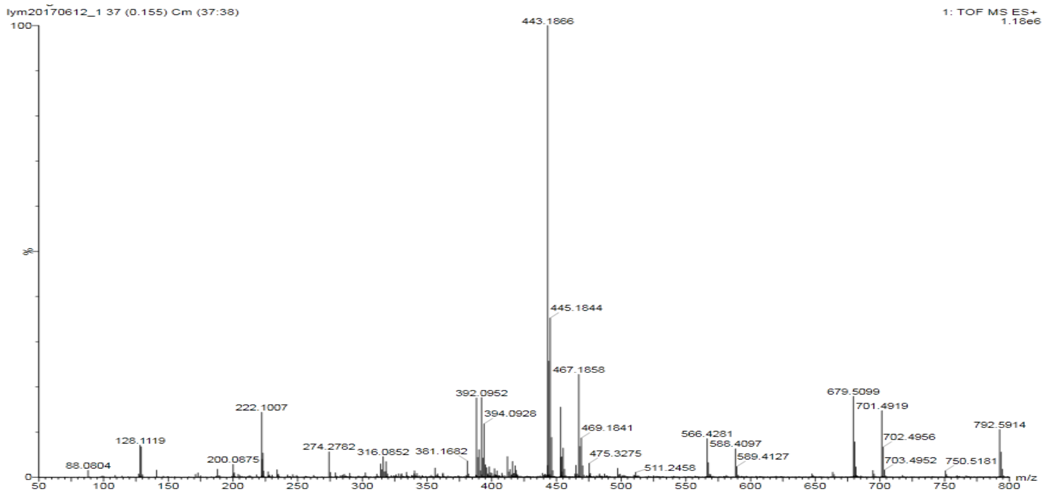

^{1}H-NMR

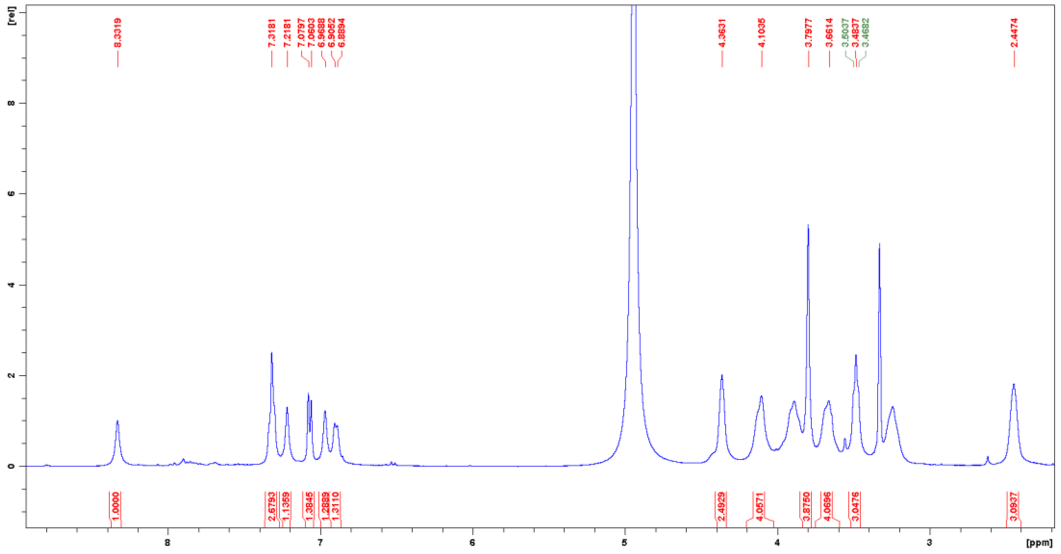

^{13}C-NMR

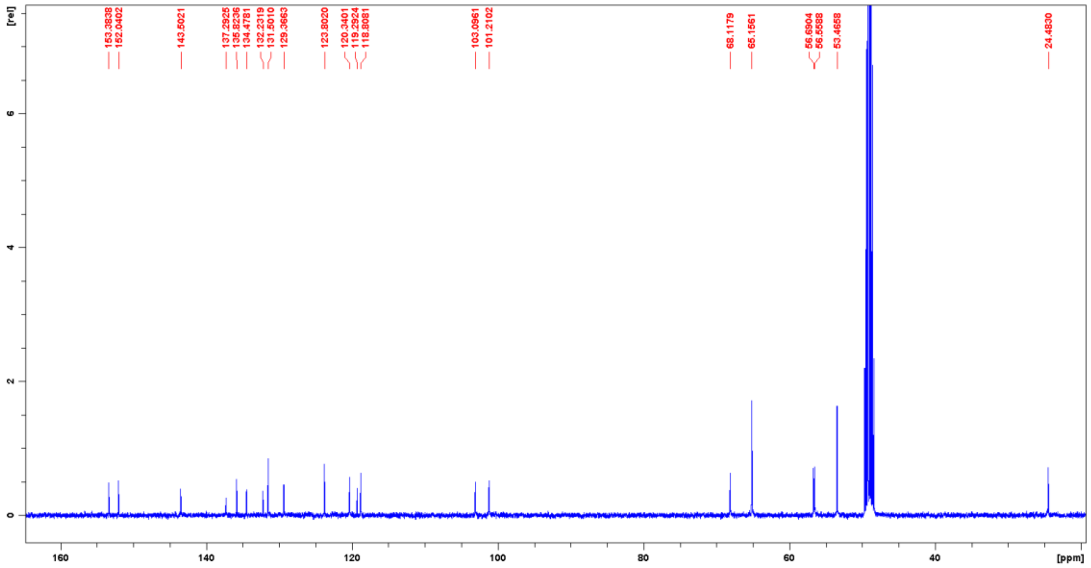

Compound 12d

HR-ESI-MS $[M+H]^+$

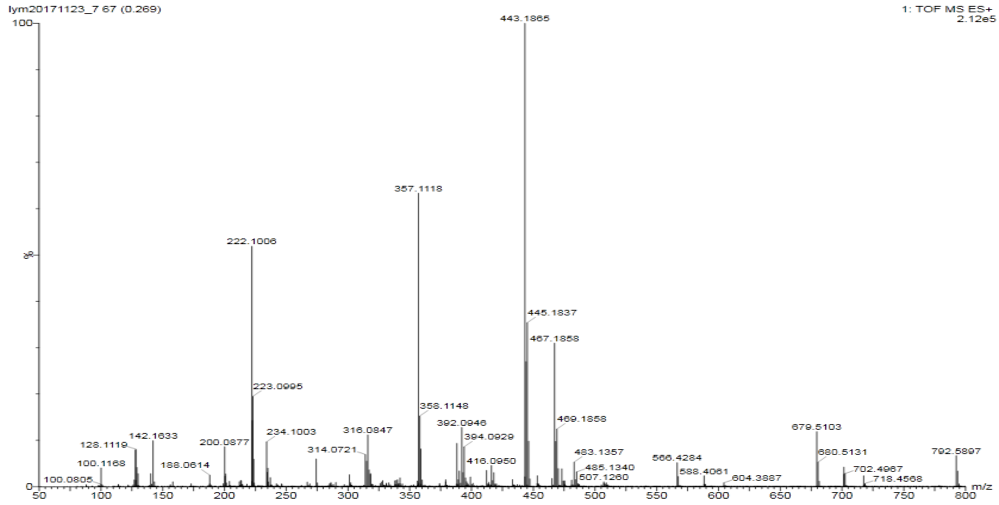

^{1}H-NMR

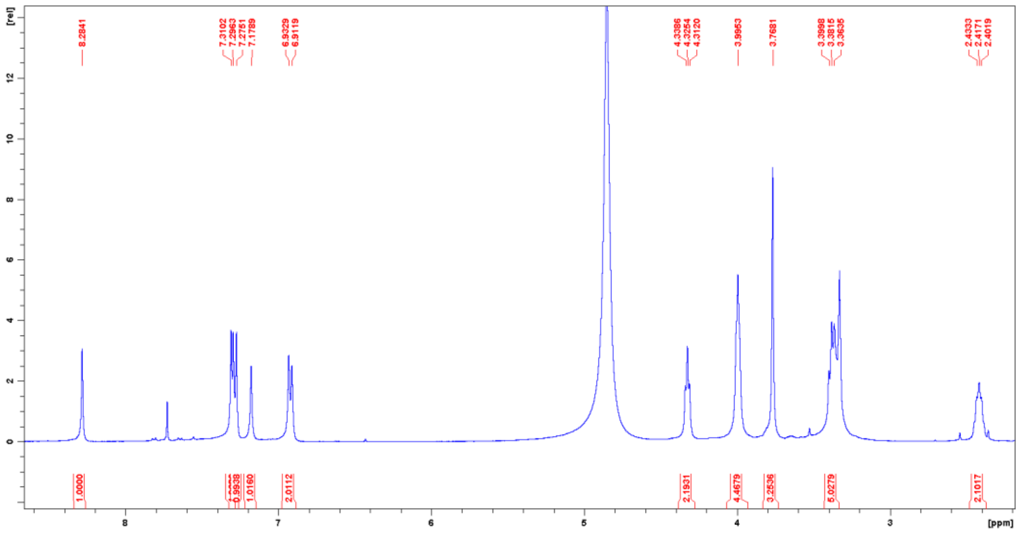

^{13}C-NMR

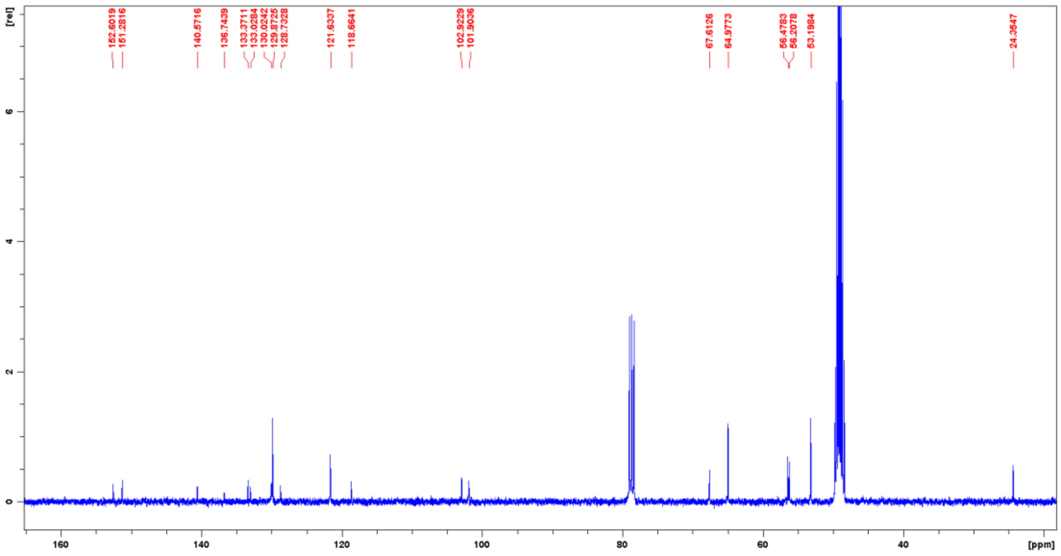

Supplement: Supplementary file 1 [file ijms-23-11231-s001.zip › ijms-1869272-supplementary.pdf]
